# The chemistry and biology of mycolactones

**DOI:** 10.3762/bjoc.13.159

**Published:** 2017-08-11

**Authors:** Matthias Gehringer, Karl-Heinz Altmann

**Affiliations:** 1 Department of Chemistry and Applied Biosciences, Institute of Pharmaceutical Sciences, ETH Zürich, Vladimir-Prelog-Weg 4, 8093 Zürich, Switzerland

**Keywords:** Buruli ulcer, mode of action, mycolactones, structure–activity relationships, target elucidation, total synthesis

## Abstract

Mycolactones are a group of macrolides excreted by the human pathogen *Mycobacterium ulcerans*, which exhibit cytotoxic, immunosuppressive and analgesic properties. As the virulence factor of *M. ulcerans*, mycolactones are central to the pathogenesis of the neglected disease Buruli ulcer, a chronic and debilitating medical condition characterized by necrotic skin ulcers. Due to their complex structure and fascinating biology, mycolactones have inspired various total synthesis endeavors and structure–activity relationship studies. Although this review intends to cover all synthesis efforts in the field, special emphasis is given to the comparison of conceptually different approaches and to the discussion of more recent contributions. Furthermore, a detailed discussion of molecular targets and structure–activity relationships is provided.

## Review

### I. Mycolactones and Buruli ulcer

Buruli ulcer is a chronic and debilitating disease characterized by skin ulcers and necrotic cutaneous lesions. Ulcers typically occur at the limbs and can extend to 15% of the skin surface if untreated. The disease is caused by the pathogen *Mycobacterium ulcerans* and represents the third most common mycobacterial infection after tuberculosis and leprosy [1–3].

At its outset Buruli ulcer usually occurs as painless subcutaneous swellings in the form of nodules, papules, plagues or diffuse edema [4]. Due to their inconspicuous appearance, early disease stages might be confused with insect bites, boils, lipomas or diverse subcutaneous infections [3]. The ulcerative stage is characterized by massive necrotic tissue destruction. Lesions are mainly located in the upper and lower limbs (35% and 55%, respectively) while only 10% occur at other parts of the body [3]. Generally, large numbers of extracellular mycobacteria are observed in all, the early, the pre-ulcerative and the ulcerative disease stage without being accompanied by granuloma formation [5]. The ulcers expand over time and can spread over the entire extremities. However, ulceration is normally not accompanied by pain and fever although those symptoms might be present in severe forms. In 5–10% of all cases, *M. ulcerans* also invades the bone and causes osteomyelitis leading to serious disabilities and severe deformities [4,6]. Although Buruli ulcer itself is rarely life threatening, untreated disease generally results in severe functional and aesthetic squeal [7] and increases the risk for dangerous secondary infections [8].

Buruli ulcer is one of currently 18 neglected tropical diseases (NTDs) according to the WHOs classification and primarily affects children under the age of fifteen. *M. ulcerans* infections have been reported from at least 33 countries, typically in tropical and subtropical regions, and represent a substantial societal and economic burden [9]. Although the vast majority of Buruli ulcer cases has been reported for Western sub-Saharan Africa (especially from Ivory Coast, Benin, Ghana and the Democratic Republic of the Congo), the pathogen is also endemic in South America, the Western Pacific region (incl. Australia) and Asia (e.g., China and Japan). In certain highly endemic regions like the Zou department in southern Benin, the prevalence of Buruli ulcer can even exceed that of tuberculosis or leprosy [10]. Since 2010, the number of reported Buruli ulcer infections worldwide has declined from almost 5000 annual cases to approximately 2000 in 2015 [11–12], but the reasons for this decrease are unknown [3]. However, these numbers have to be treated with care and numerous cases have to be assumed to remain unreported, since only 15 countries regularly report data to the WHO [3]. In addition, infections might often remain unrecognized, due to poor healthcare standards in most of the affected countries [12].

The first suspected case of a *M. ulcerans* infection was reported in the early 1860s by Captain James August Grant in his accounts of his journey with John Hanning Speke on their quest for the source of the White Nile. In his book *A walk across Africa or domestic scenes from my Nile journal* [13] a detailed description of his condition is given that reflects the symptoms of the edematous form of Buruli ulcer as it is occurring in Central and Western Africa [14]. The first clinical description of Buruli ulcer was provided in 1897 by the medical missionary Sir Ruskin Albert Cook in Kampala (Uganda) [15]. More than 50 years later, a seminal report by MacCallumn and co-workers from Bairnsdale hospital (Victoria, Australia) described six patients from rural riverine areas suffering from an unknown ulcerative infection [16]. A “mycobacterium hitherto unrecorded and pathogenic to man” was found in the patients’ lesions. Biopsy and microscopic analysis revealed a unique histopathological pattern in all patients that distinctly differed from tuberculosis. However, the germ gave the typical acid-fast stain common to all mycobacteria. As reported by Fenner et al., MacCallum later suggested the name *Mycobacterium ulcerans* [17]. Initial attempts to cultivate the bacterium failed until it was realized that, in contrast to *M. tuberculosis* that can be grown at 37 °C, *M. ulcerans* requires temperatures above 25 °C, but below 37 °C (ideally 32–33 °C) for growth [16]. This might be one of the reasons why *M. ulcerans* infections in humans are primarily limited to cutaneous tissue. Furthermore, low oxygen concentrations were later shown to be beneficial for cultivating this very slow growing mycobacterium [18]. A few years after their initial characterization, infections with *M. ulcerans* were also observed in today’s Democratic Republic of Congo [19] and in Uganda [20–22]. The name Buruli ulcer was suggested in relation to case reports from Buruli County in Mengo district (today Nakasongola district) in Uganda [20]. Although Bairnsdale ulcer would be the historically more correct denomination, the WHO approved the name Buruli ulcer.

Despite the long known association of Buruli ulcer with riverine areas and wetlands [22–23], the natural reservoir of *M. ulcerans* is still elusive and due to its obscure route of transmission [12], Buruli ulcer is sometimes referred to as the “mysterious disease” [24]. While *M. ulcerans* is believed to be an environmental pathogen [25], there is putative evidence that it can also be hosted and transmitted by living organisms such as aquatic insects [26], mosquitoes [27], fish and amphibians [28].

**Figure 1:**
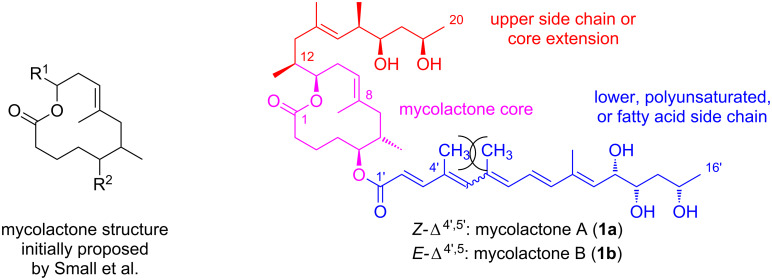
Initial proposal for the core macrolactone structure (left) and the established complete structure of mycolactones A (**1a**) and B (**1b**) (right).

Upon infection*, M. ulcerans* is usually concentrated in a small focus surrounded by a larger necrotic area that contains few bacteria. Based on this observation, Connor and Lunn speculated already in 1966 that *M. ulcerans* might excrete a diffusible toxin [21]. In 1974, two reports by Connor and co-workers corroborated this hypothesis by demonstrating that the injection of culture filtrates from different *M. ulcerans* strains into mouse footpads and guinea pig skin caused similar effects as the inoculation with the living organism [29–30]. These studies also suggested that the toxin had a molecular mass of around 100,000 Da and was moderately heat stable. Four years later, in 1978, Krieg and co-workers proposed the toxin to be a phospholipoprotein–polysaccharide complex, based on studies investigating the stability of *M. ulcerans* extracts towards different chemicals and enzymes [31]. The true nature of the toxin, however, remained elusive until 1998, when Small and co-workers identified a polyketide isolated from acetone-soluble *M. ulcerans* lipid extracts as the key virulence factor [32–33]. The initial characterization of the toxin relied on the separation of extract components by thin layer chromatography (TLC) and the biological characterization of the individual bands, a process that revealed a light yellow UV-active component to possess the highest cytopathogenic activity. Further purification of this material by reversed-phase HPLC and subsequent characterization by high-resolution mass spectrometry and two-dimensional NMR spectroscopy unveiled a 12-membered macrolactone substituted with two polyketide-derived side chains (Figure 1). Based on its mycobacterial origin and its chemical structure, this compound was named mycolactone. It is worth noting that mycolactone represented the first polyketide macrolide isolated from a mycobacterial species and was also the first example of a polyketide acting as the virulence factor of a human pathogen [34].

The purified toxin possessed similar in vitro cytopathogenicity as culture filtrates from *M. ulcerans* and caused essentially the same gross pathological and histopathological changes as a *M. ulcerans* infection. In contrast, a mycolactone-deficient *M. ulcerans* strain was not able to induce those phenotypes [32]. However, chemical complementation with mycolactone restored the typical *M. ulcerans* pathology for mycolactone-deficient strains [35]. Some chemical modifications were performed on the purified extracts showing that peracetylation or exhaustive double bond saturation by hydrogenation resulted in a total loss of cytopathogenicity. Interestingly, washing cells after mycolactone treatment restored cell growth, thus indicating at least a partial reversibility of the toxic effects.

The structural proposal for mycolactone that was offered by Small and co-workers in the context of their original report on the isolation of the toxin was only cursory. A complete two-dimensional structure was reported shortly thereafter, although both the absolute and relative stereochemistry of the molecule remained unassigned at the time [36]. Importantly, an NMR spectroscopic analysis showed that the isolated “mycolactone” in fact consisted of a 3:2 mixture of two isomeric compounds that were distinct by the configuration of the C4’–C5’ double bond in the C5 (“lower”) side chain. These isomers were consequently named mycolactone A (*Z*-isomer, **1a**) and B (*E*-isomer, **1b,** Figure 1). Although separable by reversed-phase HPLC, neither of the isomers could be isolated in pure form, presumably due to rapid (re)equilibration during or after separation. Indeed, this presumption was later proven to be true by the (attempted) targeted total synthesis of each isomer; as part of this work, mycolactones A and B were shown to rapidly equilibrate under standard laboratory conditions [37]. The prevalence of the *Z*-Δ^4’,5’^ isomer at equilibrium can be rationalized by the allylic strain [38] induced by the methyl groups attached to C4’ and C6’, respectively. The relative and absolute stereochemistry of mycolactone was then established in 2001 by Kishi and co-workers [39–40], using a combination of model compound synthesis and exploitation of an NMR database [41–42]. The correctness of the assignment was subsequently verified by total synthesis (vide infra) [43].

**Figure 2:**
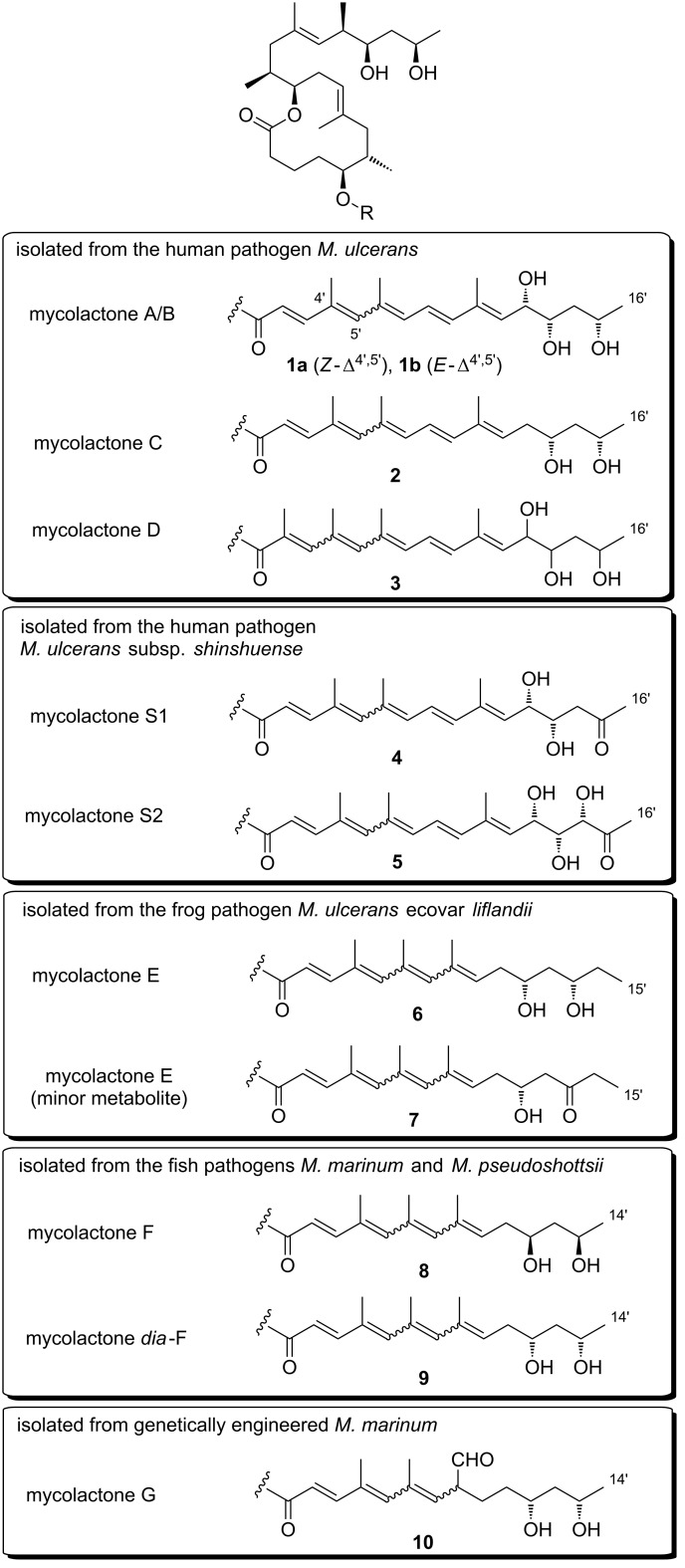
Mycolactone congeners and their origins.

After the discovery of mycolactones A/B (**1a,b**), eight congeners (mycolactones C (**2**), D (**3**), E (**6**) and its minor oxo-metabolite (**7**), F (**8**) and *dia*-F (**9**), S1 (**4**) and S2 (**5**)) (Figure 2) were discovered in extracts from different *M. ulcerans* strains and closely related mycobacteria. Given their close genetic relationship [44], it has been suggested that all currently known mycolactone-producing bacteria should be reclassified as *M. ulcerans* [45]. Within this review, however, the originally proposed species names (*M. marinum*, *M. ulcerans* ecovar *liflandii*, *M. pseudoshottsii*, *M. ulcerans* subsp. *shinshuense*) will be used.

It should also be noted at this point that the nomenclature used for mycolactones is not consistent throughout the literature. In this review, we will use the term “mycolactone A/B” to refer to the equilibrated mixture of mycolactone A and mycolactone B; in contrast, and following common literature practice, all other mycolactones (vide infra) are denoted by appending a single letter to the name mycolactone, although preparations of these different variants that are obtained either by isolation or by total synthesis are mixtures of double bond isomers and used as such in biological experiments. As for atom numbering, the carbon atoms in the 12-membered macrolactone ring are designated as C1–C11 (with the carbonyl carbon of the lactone ester group as C1), those in the carbon-linked (“upper”) side chain as C12–C20 and those of the oxygen-linked (“lower”) side chain as C1’–C16’ (with the carbonyl carbon of the exocyclic ester group as C1’). Finally, in our terminology, the term “mycolactone core” refers to the C1–C11 macrolactone ring (including the OH group on C5) without the C12–C20 side chain, while the term “extended core” encompasses the entire C1–C20 segment. Consequently, the upper side chain comprising C12–C20 will be referred to as “core extension.”

The discovery of mycolactones other than mycolactone A/B (**1a**,**b**) was initially triggered by the observation that *M. ulcerans* strains from Asia, Mexico, and Australia were apparently less virulent than African strains [46]. Intriguingly, these differences seem to translate into differences in the specific pathology of *M. ulcerans* infections [46]. For example, osteomyelitis, a pathology regularly observed in association with *M. ulcerans* infections in Benin [6] is absent in Australia or Mexico. Likewise, the plaque form of Buruli ulcer which is also found in Benin has not been reported in Australia [47]. Finally, Asian strains seem to be less virulent than their African complements [48–50]. These observations led the Small group to analyze partially purified mycolactones from *M. ulcerans* isolates of different geographical origin by TLC, (LC–)MS and in a cytopathogenicity assay [47,51]. These studies suggested the presence of at least two additional mycolactone congeners, with the dominant mycolactone variant found in Australian strains lacking one oxygen atom. Importantly, this compound, which was termed mycolactone C (**2**), had a lower cytopathogenic activity than mycolactone A/B (**1a**,**b**), thus offering a rationale for the lower virulence of Australian *M. ulcerans* strains. Asian strains contained significant amounts of a variant that was denominated mycolactone D (**3**) and which was hypothesized to contain an additional oxygen atom; in addition, the presence of minor amounts of the (non-acylated) extended mycolactone core was demonstrated. These findings were subsequently confirmed by Spencer et al. employing LC−sequential mass spectrometry (LC–MS*^n^*) analysis, which suggested that the various mycolactone congeners only differ in the exact structure of the polyunsaturated side chain. More specifically, they concluded that mycolactone C (**2**) is distinct from mycolactone A/B (**1a**,**b**) by a lack of the hydroxy group at C12’ [52], a proposal that was finally verified by Kishi and co-workers by means of total synthesis (vide infra) [53]. The structure of mycolactone D (**3**) was later re-investigated by Leadlay and co-workers applying LC−sequential and high-resolution mass spectrometry in combination with deuterium exchange experiments [54]. Instead of the additional hydroxy group proposed by the Small group, these studies provided strong evidence for mycolactone D (**3**) to feature an extra methyl group at the C2’-position. However, ultimate proof for the structure of mycolactone D (**3**) is still elusive.

**Figure 3:**
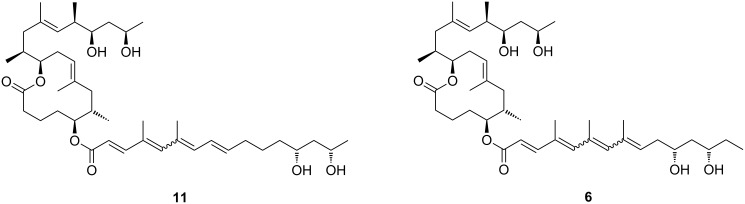
Misassigned mycolactone E structure according to Small et al. [50] (**11**) and the correct structure (**6**) first proposed by Leadlay et al. [56].

More recently, it was discovered that not only *M. ulcerans* but also the fish pathogens *M. marinum* and *M. pseudoshottsii* and the frog pathogen *M. ulcerans* ecovar *liflandii* are capable of producing mycolactones. In contrast to *M. ulcerans*, those organisms cause systemic infections [50,55], probably enabled by the lower body temperature of their poikilothermic hosts. In 2005, the Small [50] and the Leadlay [56] group independently discovered mycolactone E (**6**) from *M. ulcerans* ecovar *liflandii*, a pathogen that causes lethal infections in *Xenopus* frogs. This congener differs from mycolactone A/B (**1a**,**b**) in the lower side chain by the lack of the C8'–C9' segment, the replacement of the terminal methyl group by ethyl, and the absence of one hydroxy group. A different structure of mycolactone E (**6**) had originally been proposed by the Small group (**11**, Figure 3) after partial TLC purification and subsequent high-resolution mass spectrometry and ^1^H NMR spectroscopy (although no NMR data are shown in their report) [50]. Shortly afterwards, the Leadlay group proposed structure **6** (Figure 2) based on tandem mass spectrometry in conjunction with oxidative degradation and deuterium exchange experiments [56]. In spite of the challenge posed by the severely limited availability of natural material for structural analysis, Kishi and co-workers later demonstrated by total synthesis (vide infra) that the Leadlay structure was the correct one [57]. Besides mycolactone E (**6**), a minor metabolite (**7**) with a keto group replacing the hydroxy function at the C13’-position was found in *M. ulcerans* ecovar *liflandii* lipid extracts [50,56]. Again, the structure was finally established by Kishi and co-workers using a combination of total synthesis and HPLC on a chiral stationary phase [58]. Of note, the shorter conjugated system in these tetraenoate derivatives causes a different pigmentation of the respective mycobacteria. While *M. ulcerans* colonies generally possess a light yellow color, *M. ulcerans* ecovar *liflandii* colonies are light orange [50].

In 2006, mycolactone F (**8**), a congener found in certain fresh water fish-infesting *M. marinum* strains and in *M. pseudoshottsii*, was first described by Small and co-workers [55]. With its molecular weight of 700 Da, mycolactone F is the smallest member of the mycolactone family known to date. A structure was proposed by the Small group based on mass fragmentation and ^1^H/2D NMR spectroscopic data. This structure, which features a tetraenoate (lower) side chain with a terminal 1,3-diol motif was once again confirmed by total synthesis in the Kishi laboratory; the relative and absolute stereochemistry of the compound was assigned by NMR in conjunction with HPLC on a chiral stationary phase [59]. Of note, the stereochemistry of the 1,3-diol motif of the polyunsaturated side chain of mycolactone F (**8**) is antipodal to the same motif in all other natural mycolactones with known configuration. Intriguingly, salt water fish-infesting *M. marinum* produces a remote diastereomer [60] of mycolactone F (*dia*-mycolactone F, **9**) that exhibits the regular configuration of the 1,3-diol motif at the end of the lower side chain [61]. Most recently, the Kishi laboratory isolated two new mycolactone family members, mycolactones S1 (**4**) and S2 (**5**), from the Japanese strain *M. ulcerans* subsp. *shinshuense* [62]*.* Both of these new variants are oxidized derivatives of mycolactone A/B (**1a**,**b**) bearing a keto group at the C15’-position; in addition, mycolactone S2 incorporates an extra hydroxy group at C14’.

The first and currently only mycolactone originating from a genetically engineered biosynthetic pathway was isolated by Leadlay and co-workers in 2007 [63]. Thus, the cloning of a CYP450 hydroxylase gene from a related strain into the *M. marinum* DL045 strain produced a mycolactone F variant with a formyl group attached to C8’ and a single bond between C8’ and C9’ (mycolactone G, **10**).

**Figure 4:**
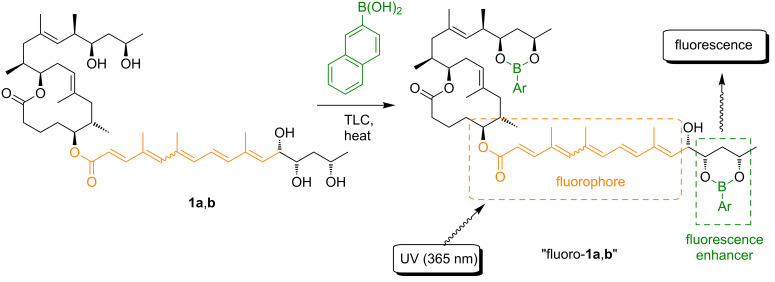
Schematic illustration of Kishi’s improved mycolactone TLC detection method exploiting derivatization with 2-napthylboronic acid as a fluorescence enhancer.

Due to its unspecific appearance at early stages, the diagnosis of Buruli ulcer is non-trivial and no point-of-care rapid diagnostic test is currently available [64]. Identification of the infection generally relies on the experience of local health professionals. Subsequent laboratory testing to confirm the clinical diagnosis might then be performed by 1) direct smear examination for acid-fast bacilli; 2) in vitro culture; 3) polymerase chain reaction (PCR), targeting the genomic region IS2404; and 4) histopathological examination [64]. Alternatively, serological testing has been proposed and promising results were obtained in a case control study in Ghana [65]. More recently, the detection of mycolactone from patient biopsy samples via LC–MS [66] and RNA aptamer binding [67] has been suggested, but the suitability of these methods for broad application in endemic areas is questionable. The WHO recommends at least two different confirmative tests for a conclusive diagnosis. In clinical practice, however, disease management without microbiological confirmation of the diagnosis is common. To improve this situation, non-invasive diagnostic tools that are cost-efficient, operationally simple and do not require sophisticated laboratory equipment are required. A method that fulfills these requirements and that relies on thin layer chromatography (TLC) for mycolactone separation was recently introduced by Kishi and co-workers [68]. While the UV-based quantification of mycolactones on TLC plates is hampered by a high detection limit (20–30 ng) and requires access to difficult to store reference samples, Kishi and co-workers have devised a more sensitive, specific detection method that is based on the chemical derivatization of mycolactone A/B (**1a**,**b**) with a 2-naphthylboronate-based fluorogenic chemosensor (Figure 4). The latter complexes the 1,3-diol moiety proximal to the pentaene motif of the lower side chain, thus resulting in enhanced fluorescence emission intensity of the mycolactone band upon irradiation with 365 nm UV light. This method allows the detection of as little as 2 ng of mycolactone within a considerably reduced background and it is specific for mycolactones A/B (**1a**,**b**), C (**2**), and D (**3**, no data for mycolactones S1 and S2 available). Mycolactones E (**6**) and F (**7**) do not yield fluorescent spots or bands. The method has been validated for a mouse footpad model of *M. ulcerans* infection [69] and for skin tissue samples from Buruli ulcer patients [70]. With a detection rate of 73%, TLC proved superior to microscopy (30–60%) or culture (35–60%) and comparable to histology (82%), but inferior to PCR (92–98%) [71–72].

Although spontaneous healing may occur in rare cases [73], early and continuous treatment is generally considered crucial for avoiding long-term damage by the Buruli ulcer disease [74]. It is beyond the scope of this review to detail the currently established or exploratory treatment options for Buruli ulcer; this topic has been reviewed elsewhere [75–76]. Suffice it to say that the WHO recommends combination treatment with oral rifampicin (10 mg/kg once daily) and intramuscular streptomycin (15 mg/kg once daily) over eight weeks [75].

### II. Biological effects of mycolactones and mechanisms of action

Although Buruli ulcer is associated with extensive fat cell necrosis at the sites of infection, the ulcers are typically accompanied by minimal pain or inflammatory response. These macroscopic observations reflect the cytotoxic [32], immunosuppressive [35,77] and analgesic [78] properties of mycolactones. Although these properties seem fairly general, mycolactone-promoted effects are still multifaceted and strongly depend on the cell line investigated. A detailed discussion of cell-type specific effects can be found in a recent review by Sarfo et al. [79]. Therefore, cellular effects of mycolactones will be discussed here only briefly, while emphasis is placed on their molecular targets.

Even at very low, non-toxic concentrations pure mycolactones or *M. ulcerans* culture supernatants suppress innate and adaptive immune response. For example, mycolactone treatment leads to a marked reduction of cytokine expression levels in human monocytes [77,80] and T-lymphocytes [77], although not all cytokines are affected [81]. In fact, the mycolactone-mediated downregulation of the immune response and prevention of the recruitment of inflammatory cells to the infection site might be crucial for Buruli ulcer pathogenesis [35,81]. With increasing concentrations, the cytotoxic effects of mycolactone become more prominent. These are typically accompanied by a profound structural change in the cytoskeleton followed by cell cycle arrest in the G_0_/G_1_ phase. Ultimately, cell death, mainly via apoptosis, is observed in vitro and in vivo [32–33,82]. It is worth mentioning that in certain cell types, e.g., adipocytes, cell death via necrosis is dominant over apoptosis [83].

The most commonly used cells to study the cytopathogenicity of mycolactones are murine L929 fibroblasts, which are extremely mycolactone-sensitive. Upon exposure to natural mycolactone A/B concentrations as low as 0.025 ng/mL (0.034 nM), L929 cells show cytoskeletal rearrangements at 12 h, cell rounding within 24 h and a loss of adhesion along with growth arrest after 48 h [32]. At this point, the effect of the toxin seems to be still reversible, since washed cells are capable of regrowth. Upon extended exposure (3 to 5 days), murine L929 fibroblasts undergo apoptosis at mycolactone A/B concentrations as low as 3 ng/mL (4 nM), while very high concentrations (15 µg/mL) cause cell death via necrosis within 4 h [35]. Interestingly, the addition of the pan-caspase inhibitor Boc-Asp(OMe)-fluoromethylketone [84] prevented apoptosis, while a normal cytopathogenic effect and subsequent cell death by necrosis was observed. Mycolactone A/B is also highly cytotoxic to keratinocytes [85], dendritic [81], and endothelial cells [86], while T cells [87–88] and macrophages [80,89] are less sensitive. Strikingly, no toxic potential was observed against human hepatoma HuH7 or human embryonic kidney HEK293 T cells [85]. Intriguingly, mycolactone A/B lacks antimicrobial activity [90], which suggests that the defense against competing microorganisms was not the evolutionary driver for the emergence of the toxin.

**Figure 5:**
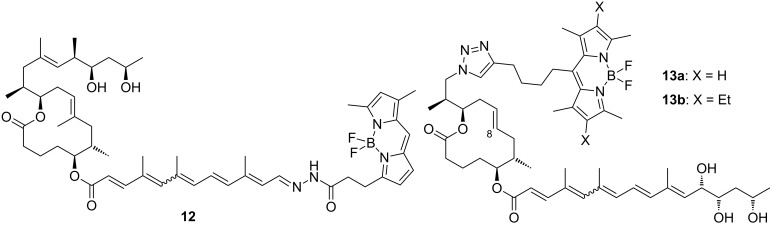
Fluorescent probes derived from natural mycolactone A/B (**1a**,**b**) or its synthetic 8-desmethyl analogs (**13a,b**).

It is generally assumed in the literature that mycolactones reach their cellular targets by passive diffusion [91]. Based on competition experiments with the fluorescent, boron-dipyrromethene (BODIPY)-labeled mycolactone analog **12** (Figure 5) which was obtained by chemical modification of natural mycolactone A/B (**1a**,**b**), Synder and Small concluded that mycolactone uptake is non-competitive and non-saturable. The compound quickly penetrated L929 fibroblasts and appeared to be localized in the cytoplasm, without any significant binding to the nucleus, mitochondria or actin being detectable. Similar results were obtained by Blanchard and co-workers with the fully synthetic 8-desmethylmycolactone analog **13**, which bears a BODIPY tag as a partial replacement for the core extension [92]. According to unpublished data from the Demangel group, corroborative results were obtained in human lymphocytes and epithelial cells exposed to a ^14^C-labeled form of the toxin [63].

Several molecular targets of mycolactone (A/B) have been identified so far, the first ones being the Wiskott–Aldrich syndrome protein (WASP) and the related neuronal Wiskott–Aldrich syndrome protein (N-WASP) that were discovered by Demangel and co-workers in 2013 based on experiments with a biotinylated mycolactone probe [93]. The WAS family comprises five scaffolding proteins that are crucially involved in the dynamic remodeling of the actin cytoskeleton [94]. While N-WASP is ubiquitously expressed, WASP is only found in cells of the hematopoietic lineage and appears to be critically involved in the regulation of the immune system [95]. WASP and N-WASP exist in a basal auto-inhibited state, a closed conformation in which the C-terminal verprolin homology, cofilin homology, and acidic (VCA) region interacts with a control region located at the N-terminus [96]. Upon cooperative binding of the cell division control protein 42 homolog (CDC42) and phosphatidylinositol 4,5-bisphosphate (PIP2), a conformational change is induced, which allows the (N-)WASP VCA domain to bind to and activate the cytoskeletal organizing complex ARP2/3, which in turn stimulates actin polymerization. Mycolactone A/B was found to bind to the CR1 domain of N-WASP and the CR7 domain of WASP about 100 times more tightly (*K*_d_ = 20–70 nM in both cases) than the natural ligand CDC42, thus triggering uncontrolled ARP2/3-mediated assembly of actin. As a consequence, mycolactone A/B causes impaired cell adhesion and defects in the migration of epithelial cells (e.g., increased cell motility accompanied by a loss of directionality). Mycolactone binding to WASP was also demonstrated by means of a fluorescent mycolactone-derived probe, which co-localized with active WASP to a small but significant extent in Jurkat T cells. At the same time, wiskostatin, a known N-WASP inhibitor [97] was found to counteract some of the effects of mycolactone (e.g., impaired cell adhesion in HeLa cells). Wiskostatin also suppressed the thinning of skin caused by mycolactone in a mouse model, thus indicating that N-WASP hyperactivation is indeed critically involved in the epidermal destruction seen in Buruli ulcer. Unfortunately, no X-ray or NMR data on WASP-bound mycolactone are available at this point and the interactions between mycolactone and WASP on a molecular level thus are still elusive.

More recently, Simmonds and co-workers [89,98] have provided evidence for a strong inhibitory effect of mycolactone on the Sec61 translocon. Earlier investigations from this group on human monocytes had indicated that the production of inflammatory mediators such as cytokines (e.g., TNF, IL-1β, IL-6, IL-10, and IP-10), chemokines (e.g., IL-8), and effector molecules like COX-2 was suppressed by subtoxic doses of purified natural mycolactone without any change in the corresponding mRNA levels [80]. A post-transcriptional mechanism was thus suggested to account for the discrepancy between mRNA and protein levels. Similar conclusions were later drawn by Demangel and co-workers [87]; intriguingly, however, mycolactone exposure affected only a subset of the proteome in human monocytes. Subsequent studies on human RAW264.7 macrophages then led to the hypothesis that rather than blocking translation, mycolactone A/B would block translocation of secretory proteins into the endoplasmic reticulum (ER) [89,99]. Nascent secretory proteins that are not translocated into the ER are usually rapidly degraded by the 26S proteasome. Consistent with this, mycolactone treatment in the presence of a proteasome inhibitor restored COX2 and TNF production in RAW264.7 cells and both proteins were found in the cytosol.

Translocation of secretory proteins into the ER is mediated by the Sec61 complex, a protein conducting channel that consists of three monomeric subunits, Sec61α, β and γ. Blockade of the Sec61 translocon by mycolactone was confirmed in several translocation assays. Of 18 cytokines produced in RAW264.7 cells after LPS stimulation, 17 were almost completely suppressed by mycolactone, generally with IC_50_ values of around 60 nM. Metabolic labeling experiments indicated that mycolactone exposure caused an almost complete blockage of the production of secretory and N-glycosylated proteins, which are generally processed in the ER [100]. In contrast, only minor changes in the levels of cytosolic proteins were detected. Similar results were obtained with human dermal microvascular endothelial cells (HDMVEC), murine L929 fibroblasts and HeLa cells. Mechanistic studies in a cell-free system then showed that mycolactone efficiently inhibited the co-translational translocation of polypeptides into the ER, while the post-translational, ribosome-independent translocation of short secretory proteins (SSPs) is only partially affected. Together with the results of cross-linking experiments, these data indicate that mycolactone interferes with the ribosome–nascent chain (RNC)-Sec61 complex. Similar conclusions were recently derived from an independent study by Demangel and co-workers, who confirmed by global proteome analysis via stable-isotope labeling with amino acids in cell culture (SILAC) [101] in T cells that mycolactone A/B is a broad-spectrum Sec61 inhibitor [102]. The mycolactone binding site on Sec61 appears to be located near a luminal plug of the Sec61α subunit, as the mutation of Arg66 in Sec61α to Gly renders Sec61 insensitive to mycolactone. The expression of this mutant in T cells restored their homing potential and effector functions, while expression in macrophages restored their IFN-γ-mediated bactericidal response, a critical factor for early host defense [103].

Interestingly, based on data from both the Simmonds as well as the Demangel group, WASP does not seem to play a major role for the immunosuppressive effects of mycolactone. Neither did the WASP inhibitor wiskostatin restore the production of secretory proteins nor did the silencing of (N)-WASP by RNA interference alter the suppression of secretory and membrane protein production by mycolactone.

The angiotensin pathway was identified as a third target of mycolactones by Brodin and co-workers in 2014 [104]. It has been known for some time that mycolactone is responsible for the local analgesia and the consequent painlessness of *M. ulcerans* infected lesions [78], a phenomenon that until recently was ascribed to the destruction of nerve bundles [78,105]. However, this assumption seems inconsistent with the fact that nerve damage only occurs at advanced stages of the infection, while lesions are painless from its very onset. In fact, Brodin and co-workers could demonstrate that the injection of either mycolactone A/B (**1a**,**b**) or a GFP-expressing *M. ulcerans* mutant into mouse footpads was associated with a rapid onset of analgesia that was reversible and not accompanied by macroscopic or ultrastructural signs of nerve destruction and hypoesthesia. Subsequent experiments revealed that mycolactone exposure caused hyperpolarization of neurons derived from PC12 cells that was mediated by the TRAAK potassium channel. Finally, screening of a siRNA library targeting 8000 host genes identified the angiotensin type II receptor (AT_2_R) as the molecular target of mycolactone, which was confirmed by genetic knockout in vitro and in vivo and by chemical inhibition. In a competition binding assay mycolactone was able to displace the potent radiolabeled agonist [^125^I]-CGP42,112A (*K*_d_ = 0.01 nM) [106–107] with an IC_50_ value of 3 µg/mL (corresponding to 4 µM). Binding of mycolactone A/B to AT_2_R was found to trigger the activation of phospholipase A2, resulting in the release of arachidonic acid. The latter can be converted into prostaglandin E2 (PGE2), which was shown to activate TRAAK channels. In line with this mechanistic model, cyclooxygenase (COX) 1 and prostaglandin-E synthase 2, which are central for PGE2 biosynthesis from arachidonic acid, were found to be essential for mycolactone-mediated hyperpolarization; in contrast, genetic or chemical abrogation of COX2-activity was inconsequential. The conclusions of Brodin and co-workers have recently been challenged by Anand and co-workers, who described a destructive effect of mycolactone A/B on human and rat nociceptive dorsal root ganglia (DRG) neurons [108]. Furthermore, mycolactone-treated DRG neurons showed a reversible and dose-dependent decline in capsaicin response, potentially indicating an interaction of mycolactone A/B with the transient receptor potential cation channel subfamily V member 1 (TRPV1, vanilloid receptor 1) [109]. On the other hand, co-treatment with either angiotensin II or the AT_2_R antagonist EMA401 [110] did not alter the morphological and functional defects provoked by mycolactone, thus putting into question the proposed role of the angiotensin II receptor in mycolactone-promoted analgesia.

**Figure 6:**
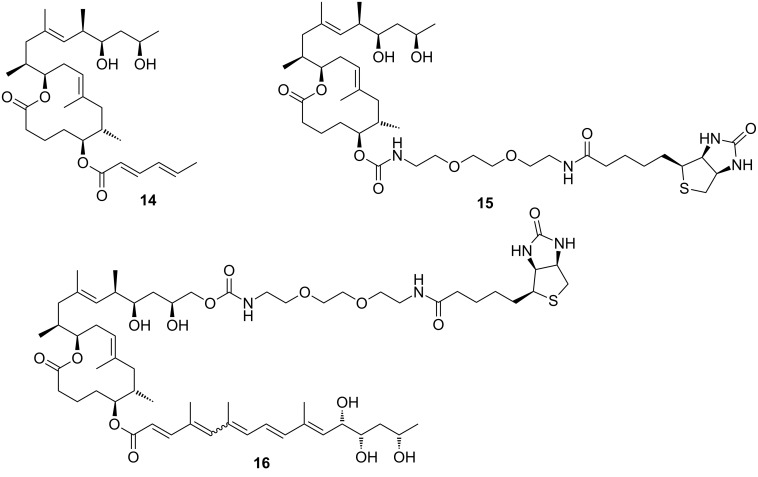
Tool compounds used by Pluschke and co-workers for elucidating the molecular targets of mycolactones.

Very recently, the Pluschke laboratory in collaboration with our own group identified the mammalian (more recently: mechanistic) target of rapamycin (mTOR) pathway as a key player in the pathogenesis of Buruli ulcer [111]. As a principal regulator of cell fate decisions, mTOR interacts with different proteins to form the multiprotein complexes mTOR complex 1 and 2 (mTORC 1 and 2), which trigger different downstream signaling cascades. As the core component of these complexes, mTOR exhibits protein kinase activity and phosphorylates a variety of downstream meditators. One of the principal substrates of the mTORC2 complex is the serine/threonine kinase Akt, which gets activated upon phosphorylation at Ser473 [112]. Disruption of the mTORC2 complex or inhibition of its kinase activity causes Akt inactivation via dephosphorylation that results in the dephosphorylation and activation of Akt-targeted transcription factors including forkhead box O1 and O3 (FoxO1 and FoxO3) [113–114]. Upon translocation to the nucleus, dephosphorylated FoxOs induce the expression of target genes such as *BCL2L11*, which encodes the pro-apoptotic Bcl-2-like protein 11, also referred to as BIM. Moreover, FoxOs can trigger apoptosis via the Fas death receptor signaling pathway [115]. In early investigations, we studied the toxicity of synthetic mycolactone A/B on L929 fibroblasts pre-treated with the pan-caspase inhibitor Z-Val-Ala-Asp-[OMe]-fluoromethyl ketone (Z-VAD-FMK) [116], the autophagy inhibitor 3-methyladenine [117] and necrostatin 1 [118], an inhibitor of programmed necrosis and found that mycolactone-treated cells die by apoptosis. Interestingly, the addition of wiskostatin, which was previously shown to counteract cytotoxic effects of mycolactones [93], even enhanced mycolactone toxicity. By using a real-time PCR (qPCR) screening of 84 genes involved in the regulation of apoptosis, autophagy and necrosis, a strong increase in the mRNA transcripts encoding for the BH3-only protein Bim and the Fas receptor was observed. Both translated into an increase of the respective protein levels and into the emergence of apoptosis markers such as cleaved caspase 3 and 8, which correlated well with the time course of mycolactone-mediated apoptosis. Silencing Bim and Fas by RNA interference proved that Bim is the key driver of mycolactone-mediated apoptosis while Fas upregulation may represent a passive bystander effect. Based on these results and considering the remote similarity of mycolactones with rapamycin, the mTOR pathway was contemplated as a potential molecular target. To put this hypothesis to test, the effect of mycolactone treatment on the phosphorylation of the mTORC1-targeted ribosomal protein S6 (rpS6) and the mTORC2-targeted kinase Akt was investigated in L929 fibroblast and Jurkat T cells. Strikingly, mycolactone treatment abolished both, S6 and Akt phosphorylation. Since mycolactone A/B did not directly interfere with the kinase activity of mTOR and caused a time-dependent gradual loss of mTORC1/2 signaling capacity, it was hypothesized that mycolactone interferes with the mTOR complex assembly. This hypothesis was confirmed by immunoprecipitation of the respective mTOR complexes at different time points after mycolactone treatment. Of note, the blockade of mTORC2 assembly by rapamycin [119] followed a similar time course as observed for mycolactone A/B. Subsequent Western blot analysis of L929 whole cell lysates proved the complete abrogation of FoxO3 phosphorylation at the Akt target site after 12 h of mycolactone treatment, which is in line with the time course of Akt inactivation. In accordance with these results, stable overexpression of constitutively active Akt (Myr-Akt) [120] rescued L929 fibroblasts from mycolactone-promoted apoptosis while silencing FoxO3 by RNA interference was only partially protective. The latter finding might be explained by the compensating effects of other FoxO proteins. Two synthetic mycolactone-derived probes bearing a biotin tag as a substitute of the lower side chain (**15**) or attached at C20 of the core extension (**16,** Figure 6) were used to investigate whether mycolactone supresses mTOR signaling in a similar fashion as rapamycin. The latter is known to bind to the FK506-binding protein FKBP12. Strikingly, target fishing in L929 whole cell lysates using these two tool compounds identified FKBP12 in the precipitates obtained with **16**, but not with **15.** In line with those observations and with published SAR (vide infra) [90], the simplified analog **14** with a truncated lower side chain caused neither inhibition of mTORC2 activity nor up-regulation of Bim. Furthermore, the suggested interaction of mycolactone A/B with FKBP12 is supported by the protective effects of an excess of FK506 against mycolactone-induced apoptosis. However, further experimental validation of the mycolactone-FKBP12 interaction, e.g., by SPR, NMR or X-ray crystallography, would be highly appreciable. Finally, we also demonstrated the key role of mycolactone-triggered Bim-mediated apoptosis in vivo. To this end, the food pads of wild-type (WT) and homozygous Bim and Fas knockout mice (Bim^−/−^ and Fas^−/−^) were infected with *M. ulcerans*. Intriguingly, WT and Fas^−/−^ mice showed the typical Buruli ulcer phenotype, while Bim^−/−^ mice were devoid of the typical Buruli ulcer-like symptoms. Moreover, Bim^−/−^ mice were able to contain the *M. ulcerans* infection suggesting that infiltrating phagocytes are able to eliminate *M. ulcerans* if they are not killed by the excreted toxin.

### III. Total synthesis of mycolactones

The fascinating biology and the challenging structural features of mycolactones have attracted significant interest from research groups worldwide with a focus on natural product synthesis. In this chapter the synthetic work on mycolactones that has been reported by the groups of Kishi, Negishi, Burkart, Altmann, Aggarwal, Gurjar, Feringa, Minnaard, Blanchard and Dai will be discussed. As a consequence of the enormous amount of work published in the field, not every single aspect of this research can be covered. While trying to be as comprehensive as necessary, we will focus on highlighting conceptual differences between different total syntheses and synthesis plans (even if not fully implemented) and exceptional chemistry that has emerged from these efforts. Moreover, a summary assessing synthetic efficiency by step count and overall yield will be provided for each synthesis. In this context, we will define a "step" as one in which a substrate is converted to a product (irrespective of the number of transformations) without intermediate workup [121]. For detailed information the interested reader is referred to the literature cited.

#### III.1. Syntheses of the mycolactone core

**Figure 7:**
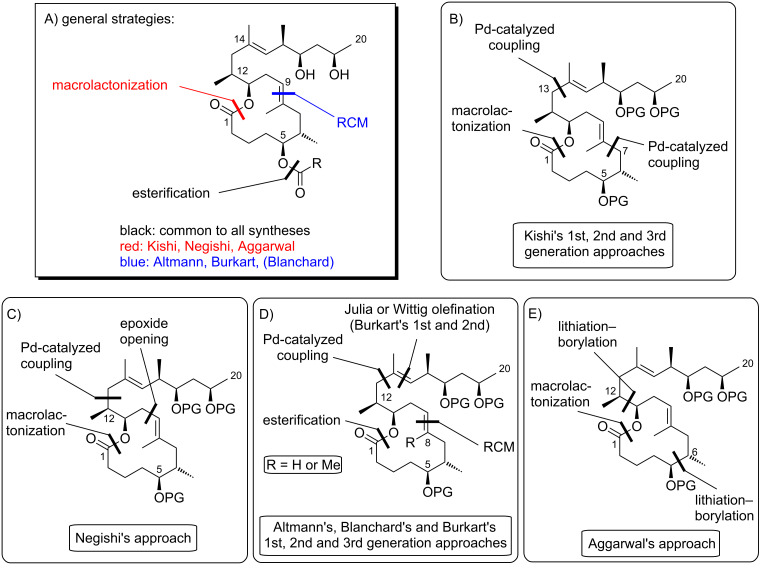
Synthetic strategies towards the extended mycolactone core. A) General strategies. B) Kishi’s approaches. C) Negishi’s approach. D) Altmann’s, Blanchard’s and Burkart’s approaches. E) Aggarwal’s approach. PG = protecting group.

Currently, all mycolactone partial and total syntheses share the (projected) final esterification of the C5-hydroxy group of the appropriately protected extended mycolactone core with the respective polyunsaturated fatty acid under Yamaguchi conditions (Figure 7). Two principal approaches have been used to establish the 12-membered macrolactone ring, namely (1) ring-closure by macrolactonization, the approach followed by Kishi, Negishi and Aggarwal, or (2) ring-closing olefin metathesis (RCM) to form the C8–C9 double bond, which is part of Burkart’s and Altmann’s syntheses of the mycolactone core and of Blanchard’s synthesis of its 8-desmethyl derivative.

A common element between Kishi’s 1st generation approach and Negishi’s and Aggarwal’s strategies consists in the assembly of the entire linear C1–C20 fragment prior to macrocyclization. For most other syntheses, namely Kishi’s 2nd and 3rd generation approaches, Burkart’s 3rd generation strategy as well as Altmann’s and Blanchard’s approaches, full elaboration of the upper (C12) side chain is performed only after formation of the macrocycle. Moreover, the majority of syntheses (all of Kishi’s syntheses, Burkart’s 3rd generation synthesis, Altmann’s and Blanchard’s syntheses) relied on the construction of the C13–C14 bond by means of palladium-mediated C(sp^2^)–C(sp^3^) cross-coupling between a C1–C13 and a C14–C20 fragment. As one of two exceptions, Negishi's mycolactone synthesis features the final assembly of the C1–C20 seco acid via formation of the C9–C10 bond by an epoxide-opening reaction with an alkyne-derived alkenyl trialkylaluminate. A distinct strategy was also chosen by the Aggarwal group, which connected the linear C1–C11 fragment to the C12–C20 fragment employing their lithiation–borylation homologation methodology; the required fragments were also obtained by the sequential application of this methodology. Of note, Burkart’s 1st generation approach aiming to assemble the cyclized C1–C14 fragment with the C15–C20 extension was unsuccessful, since the keto group located at C14 failed to undergo Wittig, HWE or Julia olefination with the respective C15–C20 fragments.

The most extensive contributions to the synthesis of mycolactones have come from Kishi and co-workers, who pioneered the synthesis of the extended mycolactone core structure. The group’s approaches to this problem have evolved over time, leading to three distinct generations of syntheses. The 1st generation synthesis [39] was developed in 2001 with the intention to confirm the mycolactone core structure, including the unambiguous assignment of its relative and absolute stereochemistry. In their 2nd generation approach [122], published in 2007, Kishi and co-workers increased the overall efficiency of the synthesis by reorganizing the assembly of the principal fragments and by optimizing the key C(sp^2^)–C(sp^3^) Negishi cross-coupling reactions as well as the choice of protecting groups. The 3rd generation approach [123], published in 2010 was developed with a main focus on scalability. Alternative access routes to key fragments allowed the efficient synthesis of multigram quantities of late stage intermediates. Finally, 1.3 g of the highly pure extended mycolactone core were prepared.

**Scheme 1:**
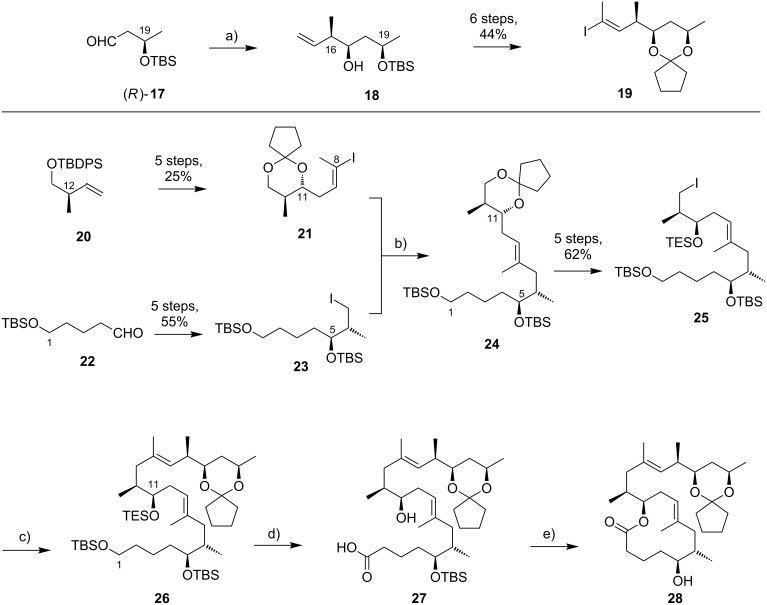
Kishi’s 1st generation approach towards the extended core structure of mycolactones. Reagents and conditions: a) (*Z*)-2-butene, *t*-BuOK, *n-*BuLi, (−)-Ipc_2_BOMe, BF_3_·OEt_2_, −78 °C, then aq H_2_O_2_ b) **23**, *t*-BuLi, ZnCl_2_, then **21**, Pd(Ph_3_P)_4_, THF, rt, 60%; c) **25**, *t*-BuLi, ZnCl_2_, then **19**, Pd(Ph_3_P)_4_, THF, rt, 50%; d) (i) HF∙pyridine/pyridine/THF (1:1:4), rt, 72%; (ii) TEMPO, *N*-chlorosuccinimide, Bu_4_NCl, CH_2_Cl_2_/pH 8.6 buffer 1:1, rt, 95%; (iii) NaClO_2_, NaH_2_PO_4_, 1,3-dimethoxybenzene, DMSO/*t*-BuOH 1:1, rt, 94%; e) (i) 2,4,6-trichlorobenzoyl chloride, DIPEA, DMAP, benzene, rt, 70%; (ii) CH_2_Cl_2_/H_2_O/TFA 16:4:1, rt, 62%.

Kishi’s 1st generation synthesis of the mycolactone core structure is depicted in Scheme 1. It relied on two consecutive Negishi cross-coupling reactions [124] to construct the linear C1–C20 fragment, which was to be cyclized by macrolactonization [39].

Vinyl iodide **19**, corresponding to the C14–C20 part of the core extension, was synthesized from literature-known aldehyde (*R*)-**17**, which defined the configuration of the C19 stereocenter (mycolactone numbering, see Figure 1). Aldehyde (*R*)-**17** can be easily prepared from commercially available methyl (*R*)-3-hydroxybutyrate ((*R*)-**47**, see Scheme 4) in two steps, namely TBS protection followed by selective reduction with DIBAL-H [125]. Aldehyde (*R*)-**17** was submitted to an asymmetric Brown crotylation reaction [126–127] to establish the C16 and C17 stereocenters. Of note, all four possible C16,C17-diastereomers of **18** were prepared (not shown) by using different combinations of (*E*)*-* or (*Z*)*-*butene and either enantiomer of methoxydiisopinocampheylborane (Ipc_2_BOMe). These compounds were required to assign the stereochemistry in the core extension by NMR spectroscopy. Homoallylic alcohol **18** was converted into vinyl iodide **19** in a high-yielding six step sequence involving ozonolysis of the double bond, Seyferth–Gilbert homologation [128–129] under Bestmann–Ohira conditions [130–131], a Schwartz hydrozirconation/iodination sequence [132], and appropriate protecting group manipulations.

Vinyl iodide **21**, which comprises the C8–C13 segment was prepared from TBDPS-protected (*R*)-hydroxy-2-methylbut-3-ene **20** that was obtained according to literature procedures [133], thus setting the stereochemistry at C12. The five-step sequence from **20** to vinyl iodide **21** included a (poorly diastereoselective) epoxidation, epoxide opening with a propynyl anion and a hydrozirconation/iodination reaction to generate the vinyl iodide moiety.

The synthesis of alkyl iodide **23** departed from TBS-protected 5-hydroxypentanal **22** and proceeded via an asymmetric Brown crotylation to establish the C5 and C6 stereocenters. Intermediates **21** and **23** were combined under Smith’s modified [134] Negishi cross-coupling [124] conditions to furnish the protected C1–C13 fragment **24**; the latter was then transformed into alkyl iodide **25** via several functional group interconversions and protecting group manipulations. Negishi cross-coupling of **25** with vinyl iodide **19** then furnished the full length intermediate **26** in moderate yield. Simultaneous removal of the secondary TES and the primary TBS ether protecting groups was followed by selective oxidation of the ensuing primary alcohol to deliver seco acid **27**. The crucial macrolactonization was performed under Yamaguchi conditions [135] in 70% yield and subsequent cleavage of the secondary TBS ether under mildly acidic conditions furnished the acetal-protected extended core structure **28**. In summary, Kishi’s 1st generation synthesis provided the extended mycolactone core in a longest linear sequence of 17 steps in 1.3% overall yield from known homoallylic silyl ether **20** [133]; the latter had to be prepared in four additional steps from commercially available (*R*)-Roche ester (*R*)-**70** (cf. Scheme 6, no yields are reported in [133] for the conversion of (*R*)-**70** into **20**).

**Scheme 2:**
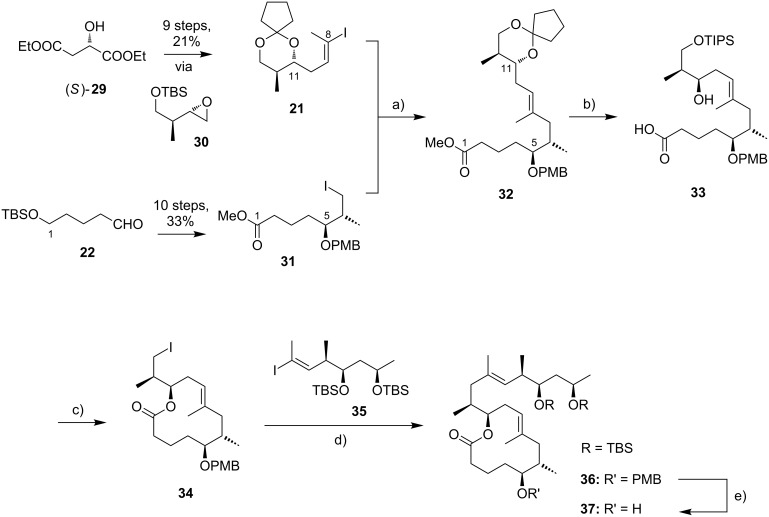
Kishi’s 2nd generation approach towards the extended core structure of mycolactones. Reagents and conditions: a) **31**, Zn/Cu, benzene/DMF 15:1, 55 °C, then **21**, Pd(PPh_3_)_4_, LiCl, NMP, 60 °C, 83%; b) (i) CH_2_Cl_2_/H_2_O/TFA 16:4:1, rt, 90%; (ii) TIPSCl, imidazole, DMF, 0 °C, quant.; (iii) LiOH, THF/MeOH/H_2_O 4:1:1, rt, 81%; c) (i) 2,4,6-trichlorobenzoyl chloride, DIPEA, benzene, then DMAP, benzene, rt, 96%; (ii) HF·pyridine/pyridine 1:1, MeCN, 0 °C, 90%; (iii) Ph_3_P, imidazole, I_2_, CH_2_Cl_2_, rt, 98%; d) (i) **34**, Zn/Cu, benzene/DMF 15:1, 55 °C, then **35**, Pd(PPh_3_)_4_, LiCl, NMP, 50 °C, 80%; e) DDQ, CH_2_Cl_2_/H_2_O, 0 °C, 91%.

Upon careful re-analysis of their 1st generation synthesis, Kishi and co-workers recognized that improvements could be made by more efficient strategies to access and assemble the chiral fragments. Moreover, they sought to employ a fully silyl-based protecting group strategy (including protection of the diol motif in the C12–C20 core extension) that would enable global deprotection after attachment of the polyunsaturated side chain. The major conceptual difference between Kishi’s 1st and 2nd generation approaches towards the extended mycolactone core structure consists in the fact that macrocyclization in the 2nd generation approach precedes Negishi coupling between a C14–C20 vinyl iodide and a C1–C13 alkyl iodide, thus making the synthesis more convergent. The synthesis of TBS-protected vinyl iodide **35** was realized by the same principle strategy as used for its cyclopentylidene-protected analog **19** in the 1st generation synthesis (cf. Scheme 1), while a distinct approach starting from diethyl (*S*)-malate ((*S*)-**29**) was used for the preparation of vinyl iodide **21**. As illustrated in Scheme 2, the nine-step synthesis of the latter proceeded via key epoxide **30** and comprised a diastereoselective alkylation of diethyl (*S*)-malate according to Seebach and Wasmuth [136] to introduce the C12-methyl group (dr = 8:1). Although being longer than the 1st generation sequence to **21**, the revised approach provided a similar overall yield and proved superior in terms of diastereoselectivity.

As for the synthesis of **23** in the 1st generation approach, alkyl iodide **31** was also prepared from aldehyde **22**. While the sequence leading to **31** was clearly longer than for **23** (10 steps vs 5 steps), the additional steps are accounted for the early adjustment of the final oxidation state at the C1-position and the protecting group change on the C5-hydroxy group from TBS to PMB. However, in terms of overall strategy, the synthesis of **31** resembles that of **23**, with an asymmetric Brown crotylation defining the C5/C6 stereochemistry as the key step.

The generation of an alkylzinc species from **31** in the presence of an ester required metalation with a zinc–copper couple [137] instead of a Li–Zn transmetalation. The efficiency of the Negishi cross-coupling between the intermediate organozinc species and vinyl iodide **21** was increased by the addition of LiCl, which is known to accelerate Stille coupling reactions [138]. Of note, a 1.4-fold excess of the alkyl iodide was needed to obtain the coupling product **32** in 83% yield. Subsequent protecting group manipulations then furnished seco acid **33**, which underwent macrocyclization to the corresponding lactone under Yamaguchi conditions in almost quantitative yield (compared to 70% for the cyclization of **27** in the 1st generation synthesis). After TIPS deprotection and Appel-type iodination [139], the ensuing alkyl iodide **34** was submitted to a second Negishi cross-coupling reaction under the conditions elaborated for the coupling of **21** and **31**, except that an excess of the vinyl iodide **35** (1.5 equivalents) was used in this case. Having fully protected intermediate **36** in hand, the final DDQ-promoted cleavage of the C5-PMB ether furnished the bis-TBS-protected extended mycolactone core **37** in 20 steps and 12% overall yield from known aldehyde **22** [140]; the latter can be prepared from commercially available 1,5-pentanediol in two additional steps.

**Scheme 3:**
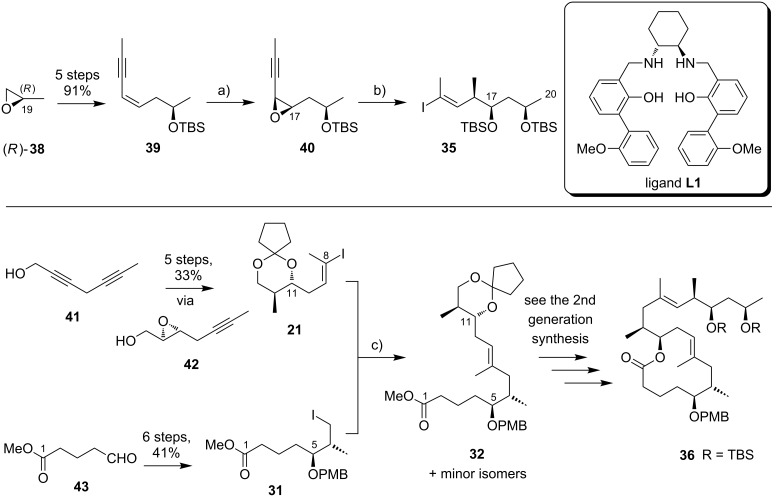
Kishi’s 3rd generation approach towards the extended core structure of mycolactones. Reagents and conditions: (a) **L1**, Ti(OiPr)_4_, 4,4′-thiobis(6-*tert*-butyl-*m*-cresol), H_2_O_2_, pH 7.4 phosphate buffer, CH_2_Cl_2_, 40 °C, 91%, ee > 99% after recrystallization; (b) (i) LiAlMe_4_, BF_3_·OEt_2_, CH_2_Cl_2_, −78 °C, 87%; (ii) TBSCl, imidazole, DMF, rt, 99%; (iii) Cp_2_Zr(H)Cl, THF, 50 °C, then I_2_, THF, 0 °C, 68%. c) **31**, Zn/Cu, benzene/DMF 15:1, 55 °C, then **21**, Pd(PPh_3_)_4_, LiCl, NMP, 55 °C, 95%.

Kishi’s 3rd generation synthesis of the extended mycolactone core differs from the two previous approaches mainly by employing alternative routes for the synthesis of vinyl iodides **21** and **35** (Scheme 3). Vinyl iodide **35** was prepared from commercially available (*R*)-propylene oxide ((*R*)*-***38**), which was opened with deprotonated TMS-acetylene. After TBS protection of the newly formed hydroxy group, iodination with *N*-iodosuccinimide followed by hydroboration/protodeboronation and Sonogashira coupling [141] with propyne gave conjugated enyne **39** in excellent overall yield. Intermediate **39** was stereoselectively epoxidized with hydrogen peroxide in the presence of titanium isopropoxide by using the Katsuki ligand **L1** [142] to give epoxide **40**, thus defining the C16/C17 stereochemistry. The C14–C20 fragment **35** was completed by selective epoxide opening with in situ generated LiAlMe_4_, TBS protection and installation of the vinyl iodide moiety by hydrozirconation/iodination. Vinyl iodide **21** was prepared from known hepta-2,5-diyn-1-ol (**41**) [143]. Briefly, the selective reduction of the hydroxymethyl-substituted alkyne **41** with LiAlH_4_ provided an allylic alcohol which underwent the key Sharpless asymmetric epoxidation [144] to furnish epoxide **42** in 69% yield and with excellent enantiomeric purity. Epoxide opening with a higher-order methyl cyanocuprate followed by TBS protection and hydrozirconation/iodination yielded the C8–C13 fragment **21**. Only minor adjustments were made to the synthesis of alkyl iodide **31**.

Starting from known aldehyde **43** [145], possessing the final oxidation state at the C1 atom, the sequence was shortened to six steps while maintaining the previous overall strategy. The connection of the fragments followed the 2nd generation logic and fine tuning of reaction conditions, most notably of the Negishi cross-coupling between **21** and **31**, led to an increased overall yield. In total, Kishi’s 3rd generation approach featured a longest linear sequence of 14 steps with an overall yield of 23% from aldehyde **43** [145], which is accessible in two additional steps from δ-valerolactone.

**Scheme 4:**
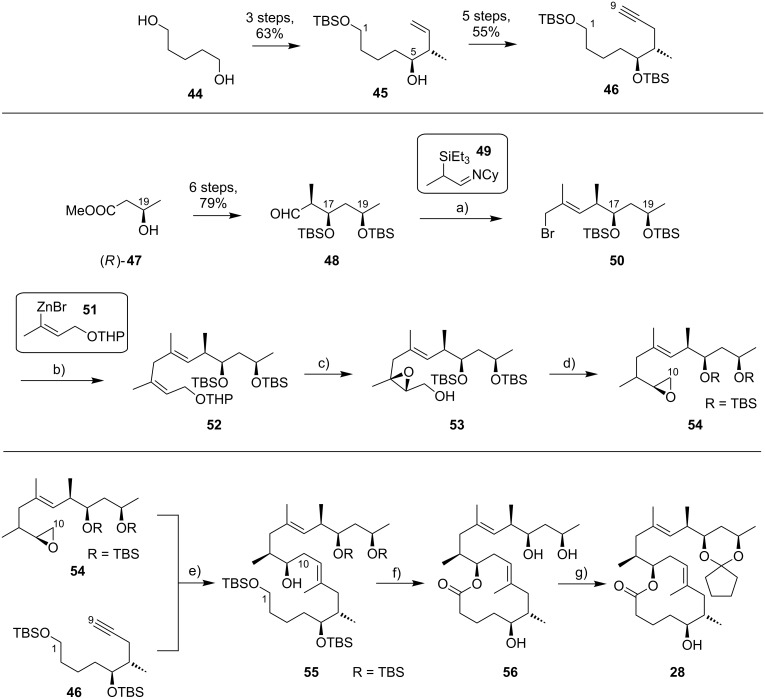
Negishi’s synthesis of the extended core structure of mycolactones. Reagents and conditions: a) (i) *s*-BuLi, THF, −78 °C to −20 °C; (ii) CF_3_COOH, THF, 0 °C, 91%; (iii) NaBH_4_, MeOH, 0 °C; (iv) CBr_4_, PPh_3_, 2,6-lutidine, CH_2_Cl_2_, 91% (2 steps); b) Pd_2_(dba)_3_, P(*o*-furyl)_3_ , DMF, 20 °C, 89%; c) (i) MgBr_2_, Et_2_O, 20 °C; (ii) Ti(OiPr)_4_, (−)-DIPT, *t-*BuOOH, CH_2_Cl_2_, −78 °C to −23 °C, 76% (2 steps); d) (i) LiBH_4_, BF_3_·OEt_2_, CH_2_Cl_2_, −40 °C, 75%; (ii) MsCl, 2,4,6-collidine, CH_2_Cl_2_, 0 °C; (iii) K_2_CO_3_, MeOH, 87% (2 steps); e) (i) **46**, Cp_2_ZrCl_2_, AlMe_3_, H_2_O, CH_2_Cl_2_, −40 °C, then *n*-BuLi, hexane, −78 °C, then **54**, Et_2_O, −40 °C to rt, then rt, 83%; f) (i) TBAF, THF, 0 °C, 78%; (ii) TEMPO, [bis(acetoxy)iodo]benzene, CH_2_Cl_2_/H_2_O 2:1, rt; (iii) NaClO_2_, 2-methyl-2-butene, NaH_2_PO_4_, *t-*BuOH/H_2_O 2:1, rt, 85% (2 steps); (iv) 2,4,6-trichlorobenzoyl chloride, DIPEA, DMAP, benzene, rt, 78%; (v) HF·pyridine, THF, rt, 86%; g) 1,1-dimethoxycyclopentane, PPTS, 80%.

An alternative approach to the extended mycolactone core that also relied on a late-stage macrolactonization was reported by the group of Negishi in 2011 [37]. They envisaged a strategy that would be heavily branded by methodologies that had been developed in their own laboratories. Moreover, increasing stereoselectivity compared to previous routes was defined as a major objective at the outset of Negishi’s work. As shown in Figure 7C, the Negishi strategy as a distinct key step features the formation of the C9–C10 bond by a zirconium-catalyzed methylalumination of alkyne **46** to form a vinyl trialkylaluminate that was reacted with epoxide **54** to obtain the linear C1–C20 fragment **55** (Scheme 4).

The synthesis of alkyne **46** followed a similar logic as Kishi’s synthesis of alkyl iodide **31**, with the contiguous stereocenters at C5 and C6 being installed by asymmetric Brown crotylation (Scheme 4, intermediate **45**). Subsequent TBS protection of the C5-hydroxy group, hydroboration of the homoallylic double bond followed by oxidation and a Corey–Fuchs reaction [146] sequence delivered stereochemically pure alkyne **46** in a total of eight steps and 35% overall yield from pentane-1,5-diol (**44**).

The preparation of epoxide **54** started from commercially available methyl (*R*)-3-hydroxybutyrate ((*R*)*-***47**) setting the stereochemistry at the C19 position. As for the elaboration of **44** into **45**, an asymmetric Brown crotylation was used to install the chiral centers at C16 and C17; after TBS protection of the newly formed hydroxy group, aldehyde **48** was then obtained by oxidative cleavage of the homoallylic double bond using the Upjohn dihydroxylation protocol [147] followed by periodate-mediated diol cleavage [148].

Aldehyde **48** was olefinated with **49** in a highly *E-*selective manner via a Corey, Schlessinger, and Mills (CSM)-modified [149–151] Peterson olefination [152] and the ensuing homologated aldehyde was subsequently converted into alkyl bromide **50** by reduction and Appel reaction. Bromide **50** was then reacted with the vinylzinc bromide **51** in an alkenyl–allyl Negishi coupling reaction [153] to deliver protected allylic alcohol **52**. A Lewis acid-promoted removal of the THP group followed by Sharpless asymmetric epoxidation of the resulting allylic alcohol furnished epoxide **53** in excellent stereochemical purity. The epoxide was then migrated to the terminal position (intermediate **54**) using a three-step procedure, thus setting the stage for the assembly of the principal fragments. In this key step, the alkenylalanate-based epoxide-opening reaction developed by the Negishi group in the 1980s [154–155] was put to test.

Thus, alkyne **46** was treated with an excess of trimethylaluminum in the presence of zirconocene dichloride to furnish the neutral methylaluminated alkene that was transformed into the respective alkenyl trialkylaluminate with *n*-BuLi. The latter selectively opened the epoxide ring in **54** to furnish the isomerically pure linear C1–C20 fragment **55** in high yield (83%). Selective removal of the primary TBS group followed by a TEMPO/Pinnick–Kraus oxidation [156–157] gave the corresponding seco acid that smoothly underwent macrolactonization under Yamaguchi conditions. Global removal of the TBS groups with HF·pyridine yielding triol **56** was followed by acid-catalyzed protection of the 1,3-diol at the core extension as the cyclopentylidene acetal, which finally led to **28**, the partially protected extended mycolactone core. Ultimately, Negishi’s synthesis of the extended mycolactone core comprised a longest linear sequence of 23 steps and 8.3% yield from commercially available (*R*)-methyl 3-hydroxybutyrate ((*R*)*-***47**).

The preparation of the extended mycolactone core via ring-closing (olefin) metathesis (RCM) [158] was first reported by the Burkart group in 2006 as part of a projected synthesis of mycolactone A/B. In addition to this alternative approach to ring closure, Burkart’s overall strategy towards the extended mycolactone core also featured a new concept for the full elaboration of the upper side chain, which was to be based on Wittig [159–160] or Julia–Lythgoe olefination [161–162] between C14 and C15. Of note, a high *E*-selectivity would be necessary in both key reactions.

**Scheme 5:**
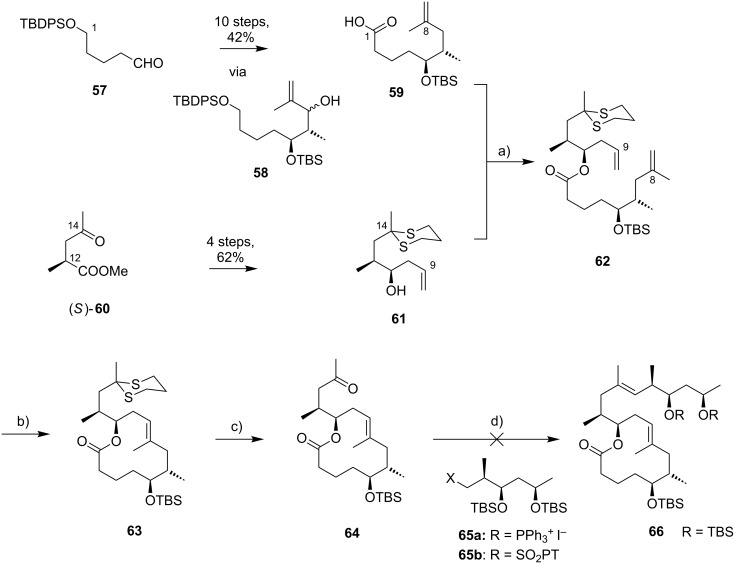
Burkart’s (incomplete) 1st generation approach towards the extended core structure of mycolactones. Reagents and conditions: a) DCC, DMAP, CSA, CH_2_Cl_2_, rt, 95%; b) Grubbs II (5 mol %), CH_2_Cl_2_, reflux, 60%; c) *N*-chlorosuccinimide, AgNO_3_, MeCN/H_2_O 4:1, 81%; d) Wittig or Julia–Kocienski olefination (undisclosed reaction conditions).

In Burkart’s 1st generation strategy, the C1–C8 fragment **59** was prepared from known aldehyde **57** [163] via an asymmetric Evans aldol reaction [164], providing the C5 and C6 stereocenters in a highly stereoselective manner (Scheme 5). TBS protection of the newly formed hydroxy group, reductive removal of the Evans auxiliary, oxidation of the resulting primary alcohol, and addition of 2-propenylmagnesium bromide to the ensuing aldehyde furnished intermediate **58**. The secondary hydroxy group was acetylated and the acetate was reduced by palladium-catalyzed transfer hydrogenolysis according to a modification of the Tsuji protocol [165]. Selective cleavage of the primary TPDPS group in the presence of a secondary TBS ether was readily achieved with NaOH in refluxing methanol. The primary alcohol was oxidized in a Swern [166]/Pinnick–Kraus oxidation sequence to obtain acid **59** in excellent overall yield.

The synthesis of the C9–C14 segment started from known methyl (*S*)-2-methyl-4-oxopentanoate ((*S*)*-***60**) [167], which was protected as the 1,3-dithiane followed by reduction of the ester moiety to the aldehyde stage. The installation of the secondary homoallylic alcohol moiety and thereby the C11 stereocenter was achieved by asymmetric allylboration using a 9-BBD-derived reagent developed by Soderquist et al. [168]. Despite being a mismatched and *anti*-Felkin addition, **61** was obtained with excellent diastereoselectivity, indicating a high level of reagent control. Secondary alcohol **61** and acid **59** were then coupled in high yield employing the Keck modification of the Steglich esterification [169–170], to furnish the crucial RCM precursor **62**. The RCM reaction was accomplished with Grubbs 2nd generation catalyst (5 mol %) [171] to deliver macrolactone **63** in 60% yield after flash-chromatographic removal of the concomitantly formed acyclic dimer and a catalyst-derived benzylidene derivative. The RCM provided the desired product with exceptional *E-*selectivity, as indicated by the lack of a NOE correlation between the C9-proton and the C8-methyl group. Removal of the 1,3-dithiane protecting group with *N*-chlorosuccinimide in the presence of silver nitrate set the stage for the final olefination that had been envisioned to complete the construction of the C1–C20 fragment (vide infra). The longest linear sequence to crystalline macrocyclic ketone **64**, whose structure was confirmed by X-ray crystallography, comprised 14 steps with an overall yield of 19% from known aldehyde **57** [172]; the latter can be obtained in two additional steps from 1,5-pentanediol.

**Scheme 6:**
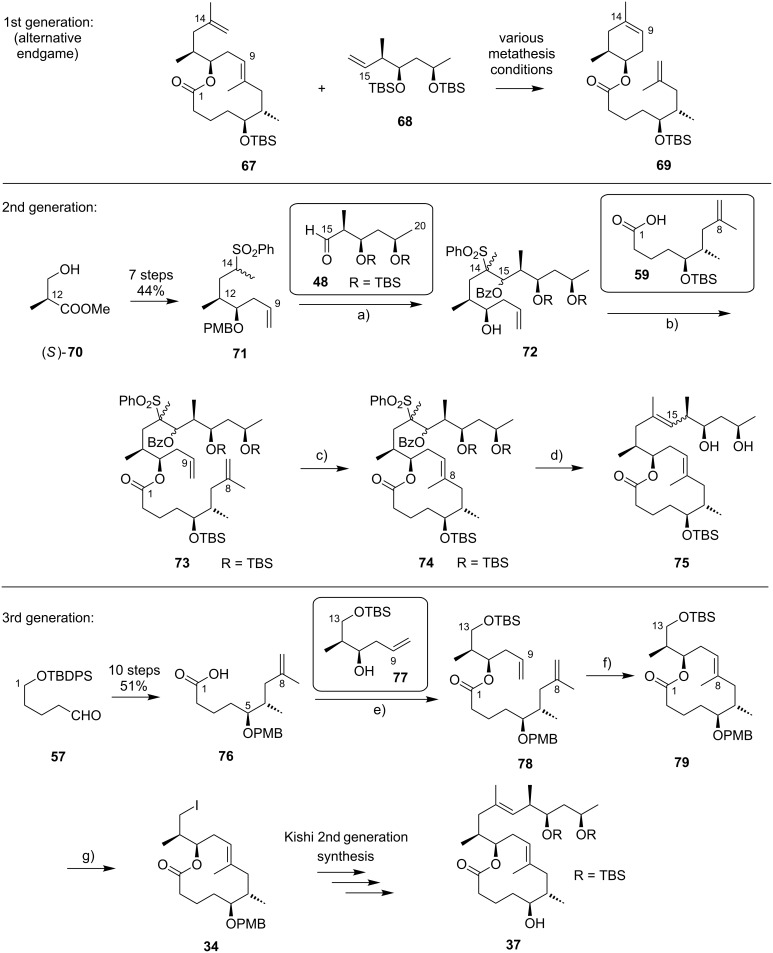
Burkart’s (incomplete) 1st, 2nd and 3rd generation approach towards the extended mycolactone core structure. Reagents and conditions: a) (i) *n*-BuLi, THF, −78 °C to −20 °C, then **48**, THF, −78 °C to −20 °C, then BzCl, −78 °C to rt, 57%; (ii) DDQ, wet CH_2_Cl_2_, rt, 95%; b) **59**, DCC, DMAP, CSA, CH_2_Cl_2_, 0 °C to rt, 96%; c) Grubbs II (4.3 mol %), CH_2_Cl_2_, reflux, 94%; d) (i) Na/Hg, MeOH, −20 °C, 90% (*E*/*Z* 2:1); (ii) TASF, DMF, 42% of *E*-isomer and 19% of *Z*-isomer; e) DCC, DMAP, pyridine, CH_2_Cl_2_, 0 °C, 87%; f) Grubbs II, CH_2_Cl_2_, reflux, 78%; g) (i) TBAF, THF, 0 °C to rt, 85%; (ii) I_2_, PPh_3_, imidazole, toluene, 0 °C, 98%.

While Burkart’s 2006 paper did not discuss the elaboration of **64** into a protected version of the extended macrolactone core, such attempts were described in a follow-up paper published in 2010 [173]. Due to problems with the originally envisaged extension of **64** at C14 by means of Wittig or Julia-type olefinations, a number of alternative strategies were explored for the elaboration of the C-linked upper side chain (Scheme 6). Initial experiments focused on cross metathesis between alkene **67**, which was accessible from ketone **64** (Scheme 5) by Wittig olefination, and known alkene **68**. A variety of conditions were investigated, all of which led to undesired intramolecular cyclization to cyclohexene ester **69** as the only isolated product, along with several side products (Scheme 6).

An alternative strategy (termed 2nd generation here) was then explored, probing the completion of the core extension by Julia olefination prior to RCM. For this purpose a route toward sulfone **71**, corresponding to the C9–C14 segment was developed. Starting from commercial (*S*)-Roche ester ((*S*)-**70**), a high-yielding seven-step sequence, employing a chelation-controlled Keck-type [174] allylation as the key step, led to **71**.

The anion of sulfone **71** was next reacted with known aldehyde **48** to furnish the Julia-olefination intermediate **72** that could be trapped with benzoyl chloride as a 12:6:1:0.5 mixture of diastereomers. Elimination of **72** to form the corresponding olefin was postponed to a later stage of the synthesis, in order to avoid intramolecular cyclization as it had been observed for **67** during attempted cross metathesis with **68**. Instead, oxidative removal of the PMB group followed by esterification with acid **59** yielded the full-length linear precursor **73** ready for cyclization. RCM to macrolactone **74** with 2nd generation Grubbs catalyst proceeded in excellent yields. Although the *E*/*Z*-selectivity of the RCM was not commented on, one may assume that the *E-*isomer was formed exclusively, based on Burkart’s previous results with diene **62**. Treatment of **74** with sodium amalgam then gave a 2:1 mixture of the *E*- and *Z*-olefins, regardless of the configuration of the starting diastereomer. Finally, removal of the TBS groups at the core extension with tris(dimethylamino)sulfonium difluorotrimethylsilicate (TASF) gave diol **75** in 8.1% yield over 13 steps from (*S*)-Roche ester (*S*)-**70**, while no suitable deprotection method for the C5-TBS ether was found. This is in sharp contrast to results from Kishi [39] who removed the C5-TBS ether under mildly acidic conditions and Negishi [37] and Aggarwal [175] who performed global TBS deprotection of the same intermediate using HF·pyridine in very good yield (see Scheme 1, Scheme 4, Scheme 11). Due to the issues encountered with the cleavage of the C5-TBS ether, the Burkart group developed a 3rd generation RCM-based access to the mycolactone core that relied on PMB protection of the C5-hydroxy group. This strategy was guided by a prior work by Kishi and co-workers, who had already demonstrated that a PMB ether masking the C5-hydroxy group could be readily removed by oxidation with DDQ [122]. To this end, C5-OPMB-protected acid **76** was prepared from aldehyde **57** in 10 steps and 51% yield (Scheme 6). The synthesis was performed in analogy to the approach depicted in Scheme 5, with the notable difference that a Crimmins thiazolidinethione auxiliary was used to enable the selective formation of the C5 and C6 stereocenters in a TiCl_4_-mediated aldol addition. After Keck-modified Steglich esterification with literature-known alcohol **77** [176], the stage was set for RCM. Cyclization of diene **78** with 2nd generation Grubbs catalyst furnished macrolactone **79** in good yield. The subsequent cleavage of the TBS ether at the C13 position followed by iodination under Appel conditions gave alkyl iodide **34** that was further elaborated into the extended mycolactone core **37** following Kishi’s lead [122]. The Burkhart synthesis provided iodide **34** in 14 linear steps and 29% yield from known aldehyde **57**.

In early 2007, shortly after the publication of Burkart’s initial work, our own group reported a distinct synthesis of the mycolactone core structure via RCM [177]. The approach delivered alkyl iodide **91** that was further elaborated into the extended mycolactone core and, ultimately, the entire natural product (as reported in 2011) [178].

**Scheme 7:**
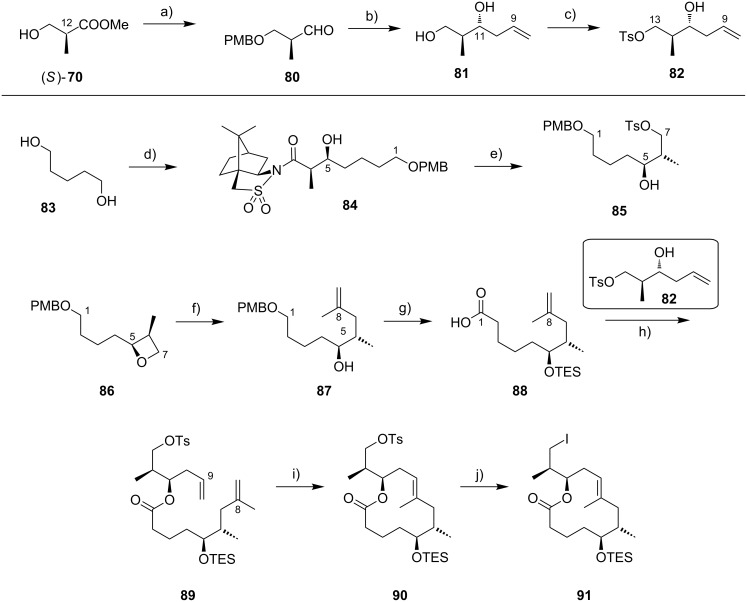
Altmann’s synthesis of alkyl iodide **91**. Reagents and conditions: a) (i) PMB-trichloroacetimidate, TfOH, Et_2_O, rt, 58%. (ii) DIBAL-H, CH_2_Cl_2_, −78 °C, quant.; b) (i) allyl–SnBu_3_, SnCl_4_, CH_2_Cl_2_, −90 °C, 82%, dr > 20:1; (ii) DDQ, H_2_O, CH_2_Cl_2_, rt; (iii) LiAlH_4_, Et_2_O, 0 °C to rt, 76% (2 steps); c) TsCl, Et_3_N, DMAP, CH_2_Cl_2_, 35 °C, 86%; d) (i) PMBCl (0.15 equiv), NaH, benzene, reflux, 97%; (ii) (COCl)_2_, DMSO, Et_3_N, CH_2_Cl_2_, −78 °C to rt, 99%; (iii) *N*-propionyl-(2*R*)-bornane-(10,2)-sultam, Et_2_BOTf, CH_2_Cl_2_, −5 °C, then addition of aldehyde, −78 °C, 83%, >95% de; e) (i) LiAlH_4_, THF, 0 °C to rt, 78%; (ii) TsCl, Et_3_N, DMAP, CH_2_Cl_2_, 0 °C to rt, 96%; f) NaH, THF, rt to 40 °C, 98%; g) isopropenyllithium, BF_3_·Et_2_O, Et_2_O, −78 °C, 90% (optimized: 97%; Gehringer & Altmann, unpublished). g) (i) TESOTf, 2,6-lutidine, CH_2_Cl_2_, −78 °C to rt, 98%; (ii) DDQ, CH_2_Cl_2_, buffer pH 7.2, rt, 92%; (iii) DMP, CH_2_Cl_2_, 0 °C to rt; (iv) NaClO_2_, 2-methyl-2-butene, NaH_2_PO_4_, *t-*BuOH/H_2_O 9:2, rt, 91% (2 steps); h) DCC, DMAP, CH_2_Cl_2_, 0 °C to rt, 82%; i) Grubbs II (12 mol %), CH_2_Cl_2_, reflux, 80%; j) NaI, acetone, rt to 65 °C, 95%.

The synthesis of the C9–C13 fragment started from (*S*)-Roche ester ((*S*)-**70**), thereby setting the stereochemistry at the C12-position (Scheme 7). After PMB protection of the hydroxy group and reduction of the protected ester to the corresponding aldehyde **80**, a chelation controlled Keck-type allylation with allyltributyltin in the presence of tin tetrachloride furnished diol **81** with high diastereoselectivity. Selective tosylation of the C13-hydroxy group completed the synthesis of this fragment (**82**). The C1–C8 fragment containing the carboxylic acid moiety was prepared from 1,5-pentanediol (**83**). In the initial steps this involved mono-PMB protection of **83**, Swern oxidation of the resulting mono-protected diol, and an Oppolzer aldol reaction [179] with the ensuing aldehyde to provide **84** with a dr > 20:1. Reductive removal of the Oppolzer auxiliary followed by selective tosylation of the primary hydroxy group gave tosylate **85**.

Direct substitution of the tosyl group with isopropenyllithium failed, however, and so did the attempted copper-catalyzed reaction with the corresponding iodide. Therefore, a two-step procedure was applied. Upon treatment with sodium hydride, tosylate **85** was cleanly converted into oxetane **86**, a stable intermediate suitable for extended storage periods. Regioselective opening of **86** with isopropenyllithium in the presence of BF_3_·etherate gave terminal alkene **87** in excellent yield. After TES protection of the unmasked secondary hydroxy group, PMB cleavage with DDQ followed by a Dess–Martin [180]/Pinnick–Kraus oxidation sequence gave acid **88**. The esterification of **88** with secondary alcohol **82** under Höfle–Steglich conditions [181] proceeded smoothly and gave key diene **89** in very good yield. An RCM was achieved with Grubbs 2nd generation catalyst in refluxing methylene chloride.

Since yields for the RCM reaction varied over a wide range without any changes in reaction conditions (mostly between 50% and 60%), a screening of alternative catalysts and solvents was performed; however, these efforts proved to be futile (Gehringer & Altmann, unpublished). These findings mirror those made in the Blanchard laboratory as part of their work on 8-desmethylmycolactones [182].

**Scheme 8:**
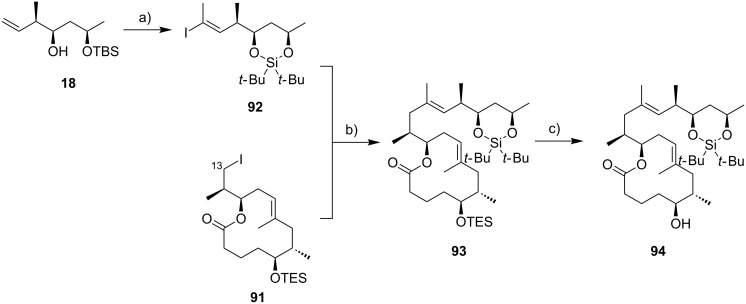
Final steps of Altmann’s synthesis of the extended core structure of mycolactones. Reagents and conditions: a) (i) TBAF, THF, rt, 83%; (ii) *t-*Bu_2_Si(OTf)_2_, pyridine, CH_2_Cl_2_, 0 °C, 87%; (iii) O_3_, CH_2_Cl_2_, −78 °C, then Me_2_S, PPh_3_, −78 °C to rt, 84%; (iv) CBr_4_, PPh_3_, CH_2_Cl_2_, 0 °C, 88%; (v) *n-*BuLi, MeI, THF, −78 °C, 79%; (vi) Cp_2_Zr(H)Cl, THF, 45 °C, then I_2_, 0 °C, 95% (Gehringer, Bucher & Altmann, unpublished); b) **91**, 9-MeO-9-BBN, *t-*BuLi, Et_2_O, THF, −78 °C to rt, then **92**, [Pd(dppf)Cl_2_], AsPh_3_, Cs_2_CO_3_, DMF, rt, 80% (optimized: 97%; Gehringer & Altmann, unpublished); c) THF/H_2_O/AcOH (2:1:1), rt, 90%.

The macrocyclic tosylate **90** was then converted into iodide **91** under Finkelstein conditions [183] to enable chain extension by C(sp^2^)–C(sp^3^) cross-coupling. Since the attempted coupling of **91** with the Kishi vinyl iodide **35**, either under modified Suzuki [184] or Negishi conditions did not furnish any of the desired product, an adjustment of the protecting group strategy was made at the stage of the vinyl iodide fragment: The two TBS ethers in **35** were cleaved and a cyclic bis*-tert*-butylsilyl ether was installed to mask the 1,3-diol moiety (**92**) (Scheme 8). Strikingly, the reduced steric hindrance of this protecting group enabled the 9-MeO-9-BBN-promoted C(sp^2^)–C(sp^3^) Suzuki coupling [184] giving rise to the complete extended mycolactone core **93**. Most recently, a more concise route furnishing vinyl iodide **92** from known homoallylic alcohol **18** in six steps and 40% yield was developed (Scheme 8, Gehringer, Bucher & Altmann, unpublished). Moreover, the yields for the Suzuki coupling reaction could be improved to up to 97% (Gehringer & Altmann, unpublished). Cleavage of the secondary TES ether under mildly acidic conditions furnished the key intermediate **94**, ready for acylation with the lower side chain. Up to this point the synthesis comprised a longest linear sequence of 16 steps and gave **94** in overall yields up to 26%, if the optimized conditions were employed.

**Scheme 9:**
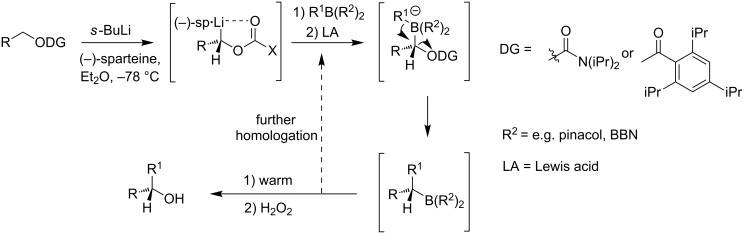
Basic principles of the Aggarwal lithiation–borylation homologation process [185–186].

The most recent and probably most elegant contribution to the synthesis of the extended mycolactone core has been made by Aggarwal and co-workers [175]. Besides the goal of providing material for biological studies, the Aggarwal group also adopted the synthesis of the extended mycolactone core as a case study to demonstrate the usefulness of their recently developed lithiation–borylation methodology [185–186] in a highly complex molecular setting. The Aggarwal methodology involves three steps [186]: 1) the generation of a chiral lithium carbenoid, typically by enantioselective Hoppe-type lithiation [187] of *N*,*N*-dialkyl carbamates in the presence of (+)- or (−)-sparteine; 2) electrophilic trapping with the organoboron reagent that usually occurs with retention of configuration; and 3) *anti-*1,2-metallate rearrangement substituting the carbamate leaving group by the migrating group on the boron atom (Scheme 9). This methodology enables simple desymmetrization in a largely reagent-controlled manner without any matching issues and it allows to perform iterative homologations to generate consecutive stereocenters.

**Scheme 10:**
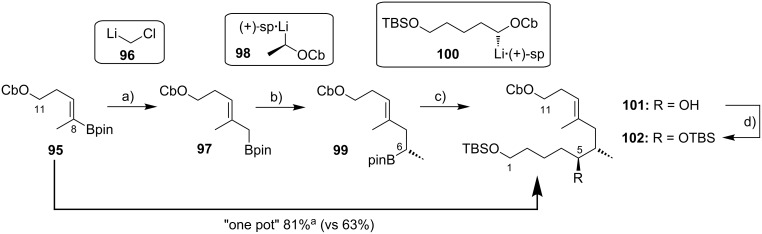
Aggarwal’s synthesis of the C1–C11 fragment of the mycolactone core. Reagents and conditions: a) ClCH_2_I, *n-*BuLi, Et_2_O, −95 °C, 99%; b) EtOCb, (+)-sparteine, *s-*BuLi, Et_2_O, −78 °C, then **97**, −78 °C to 40 °C, 83%, er 97:3; c) (i) 5-TBSO-pentyl-OCb, (+)-sparteine, *s-*BuLi, Et_2_O, −78 °C, then **99**, −78 °C to 40 °C; (ii) NaOH/H_2_O_2_, THF, 0 °C, 77% (2 steps), dr 94:6*;* d) TBSCl, imidazole, DMF, 25 °C, 82%. OCb = *N,N*-diisopropyl carbamate. ^a^One pot = sequential reactions without intermediate purification.

The Aggarwal synthesis of the extended mycolactone core started from commercially available pent-3-yn-1-ol that was transformed into vinyl boronate **95** by means of a copper-catalyzed regioselective hydroboration followed by protection of the ensuing hydroxy group as the *N,N*-diisopropyl carbamate (not shown). Matteson one-carbon elongation [188] with in situ generated chloromethyllithium (**96**) then furnished allyl boronate **97** (Scheme 10). Further homologation with asymmetrically lithiated *N,N*-diisopropyl ethyl carbamate **98** elaborated the C6-stereocenter (**99**) in good yield and with excellent enantioselectivity.

The C1–C5 fragment was then introduced by homologation with chiral lithiated carbamate **100** that was accessible from 1,5-pentanediol in a simple two-step protection sequence. The high diastereomeric ratios obtained in this lithiation–borylation step highlight the level of reagent control mediated by lithiated carbamate **100**. Oxidative cleavage of the boronate furnished secondary alcohol **101** and subsequent TBS protection led to key intermediate **102**. Interestingly, the three consecutive homologation reactions from **95** to **101** could also be performed sequentially without intermediate purification (termed “one pot” by the authors) increasing the yield from 63% to 82% over three steps.

**Scheme 11:**
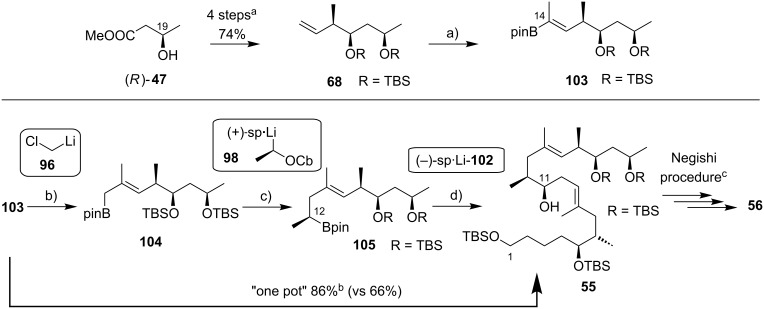
Aggarwal’s synthesis of the linear C1–C20 fragment of the mycolactone core. Reagents and conditions: a) isopropenylboronic acid pinacol ester, Hoveyda–Grubbs II catalyst (10 mol %, sequentially added), CH_2_Cl_2_, periodic degassing, 60%, *Z*/*E* > 99:1; b) ClCH_2_I, *n-*BuLi , Et_2_O, −95 °C, 99%; c) EtOCb, (+)-sparteine, *s-*BuLi, Et_2_O, −78 °C, then **104**, −78 °C to 40 °C, 81%, dr 97:3; d) (i) **102**, (−)-sparteine, *s-*BuLi, Et_2_O, −78 °C, then **105**, −78 °C to 40 °C; (ii) NaOH/H_2_O_2_, THF, 0 °C, 82%, (2 steps). ^a^Procedure according to [37,39,178]. ^b^One pot = sequential reactions without intermediate purification. ^c^Procedure according to [37].

Vinyl boronate **103**, corresponding to the C14–C20 segment of mycolactones was prepared via alkene **68** (cf. Scheme 6), which was accessed from methyl (*R*)-3-hydroxybutyrate ((*R*)-**47**) using the Kishi approach [122]. Cross metathesis with isopropenylboronic acid pinacol ester using Hoveyda–Grubbs 2nd generation catalyst [189] under optimized conditions gave **103** in moderate yield (Scheme 11). Matteson one-carbon homologation to **104** followed by another homologation with enantioselectively lithiated *N,N*-diisopropyl ethyl carbamate **98** produced the C12 stereocenter (**105**) with excellent diastereoselectivity.

Subsequently, **105** was stereoselectively elongated with lithiated key intermediate **102** followed by oxidative cleavage of the boronate to yield the complete linear C1–C20 fragment **55**. Again, performing the reaction sequence from **103** to **55** in “one pot” increased the yield from 66% to 81% over three steps.

With known intermediate **55** in hand, the endgame was realized according to Negishi’s approach [37] and gave the unprotected extended mycolactone core in 45% yield over 5 more steps. The Aggarwal synthesis outcompetes the other published syntheses in terms of longest linear sequence (11 or 13 steps, respectively, vs 14 steps [123]) and total step count (15 or 19 steps, respectively, vs 26 steps [173]), but not in terms of overall yield (17% and 13%, respectively, vs 23% [123]). The optional implementation of “one pot” reaction sequences suggest that this synthesis may be performed in a very time-efficient manner. In addition, the synthesis proved to be scalable (950 mg of intermediate **55** were produced in a single batch) and most of the expensive sparteine required for the stereoselective homologations can be recovered.

#### III.2. Synthesis of the lower mycolactone side chain

**Figure 8:**
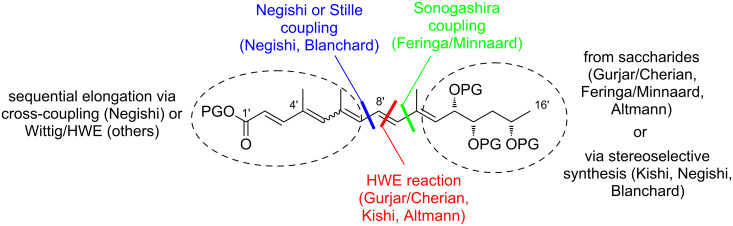
Synthetic strategies towards the mycolactone A/B lower side chain.

A general feature of all syntheses of the mycolactone A/B polyunsaturated side chain is the convergent late stage assembly of two fragments of similar size (Figure 8). The pioneering approach by Gurjar and Cherian connecting the C8’–C9’ double bond by HWE olefination was adopted by the groups of Kishi and Altmann, while the Negishi and the Blanchard groups opted for fragment assembly between the C7’ and the C8’ atoms by C(sp^2^)–C(sp^2^) cross-coupling reactions. A disconnection between the C9’ and the C10’ atom was envisaged by the groups of Feringa and Minnaard who intended to join their fragments by C(sp)–C(sp^2^) cross-coupling followed by selective reduction of the generated internal triple bond.

The western trienoate fragment is usually built up by Wittig two-carbon elongation cycles, with the notable exception of the Negishi approach, which relied exclusively on (hydro/carbo)metalation and cross-coupling reactions. The eastern fragment incorporating the three chiral centers (C12’, C13’ and C15’) was either constructed by chiral pool synthesis from monosaccharides (Gurjar/Cherian, Feringa/Minnaard and Altmann), by a strategy relying solely on asymmetric synthesis (Blanchard) or by mixed approaches (Kishi/Negishi). The convergent strategy based on the assembly of two advanced fragments was also pursued in the synthesis of the pentaenoate chains of mycolactones C, S1 and S2, while the tetraenoate chains in mycolactones E and F were constructed completely by iterative elongation cycles. As an exception, the mycolactone E side chain was prepared by Wang and Dai via connection of the C1’–C7’ and the C8’–C15’ fragments by Suzuki cross-coupling.

**III.2.1. Synthesis of the mycolactone A/B pentaenoate side chain:** In 2001, Gurjar and Cherian were the first to complete the synthesis of the protected mycolactone fatty acid side chain [190]. Their retrosynthetic analysis involved a Horner–Wadsworth–Emmons (HWE) [191–192] reaction to assemble the pentaene from a triene harboring the requisite phosphonate and an α,β-unsaturated aldehyde bearing the triol moiety. Due to the unknown stereochemistry at the C12’, C13’ and C15’ position at the beginning of their synthetic endeavor, Gurjar and Cherian needed a flexible approach towards this eastern fragment. They opted for a chiral pool synthesis starting from different 4,6-deoxyhexoses that would eventually define the stereochemistry of the triol moiety.

**Scheme 12:**
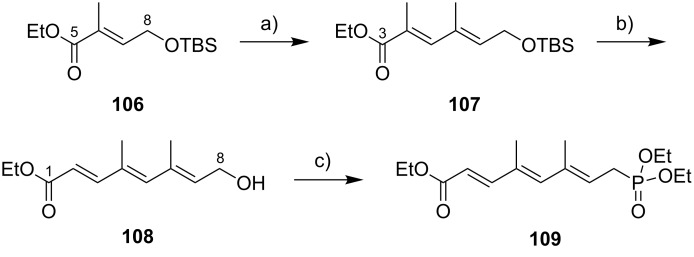
Gurjar and Cherian’s synthesis of the C1’–C8’ fragment of the mycolactone A/B pentaenoate side chain. Reagents and conditions: a) (i) DIBAL-H, CH_2_Cl_2_, −78 °C; (ii) MnO_2_, CHCl_3_, rt; (iii) Ph_3_P=C(Me)COOEt, benzene, reflux, 84% (2 steps); b) (i) DIBAL-H, CH_2_Cl_2_, −78 °C, 92%; (ii) MnO_2_, CHCl_3_, rt; (iii) Ph_3_P=CHCOOEt, benzene, reflux, 83% (2 steps); (iv) TBAF, THF, rt, 93%; c) (i) PBr_3_, Et_2_O, 0 °C; (ii) P(OEt)_3_, 90 °C, 64% (2 steps).

The western triene fragment was prepared starting from α,β-unsaturated ester **106**, which is readily accessible from ethylene glycol or allyl alcohol in a three-step protection, oxidation, Wittig reaction sequence [193]. The ester **106** was then reduced to the corresponding allylic alcohol with DIBAL-H, oxidized with MnO_2_ and the ensuing aldehyde was olefinated with ethyl 2-(triphenylphosphoranylidene)propionate to furnish diene **107** (Scheme 12).

The same three-step homologation procedure was repeated with ethyl (triphenylphosphoranylidene)acetate as the Wittig reagent, giving triene **108** upon TBS deprotection. The transformation of the primary hydroxy group to the respective bromide with PBr_3_ was succeeded by conversion to phosphonate **109** in a Michaelis–Arbuzov reaction [194–195] with neat triethyl phosphite.

**Scheme 13:**
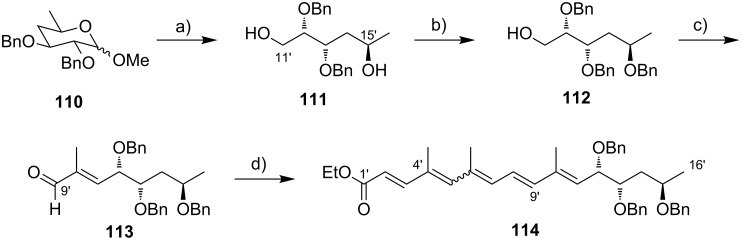
Gurjar and Cherian’s synthesis of the benzyl-protected mycolactone A/B pentaenoate side chain. Reagents and conditions: a) (i) H_2_SO_4_, dioxane/water 2:1, 100 °C; (ii) NaBH_4_, MeOH, 0 °C, 54% (2 steps); b) (i) TBSCl, imidazole, CH_2_Cl_2_, rt; (ii) BnBr, NaH, DMF, rt; (iii) TBAF, THF, rt, 75% (3 steps); c) (i) (COCl)_2_, DMSO, Et_3_N, −78 °C; (ii) Ph_3_P=C(Me)COOEt, benzene, reflux, 80%; (iii) DIBAL-H, CH_2_Cl_2_, −78 °C, 94%; (iv) MnO_2_, CHCl_3_, rt; d) **109**, LDA, THF, −78 °C to 0 °C, 65% (*Z*-Δ^4’,5’^/ *E*-Δ^4’,5’^ 3:2).

The synthesis of the eastern fragment started from benzylated methyl 4,6-dideoxy-D-glucose **110**, which was hydrolyzed with sulfuric acid and reduced with sodium borohydride to give the dibenzylated tetraol **111** (Scheme 13). For selective benzylation of the secondary hydroxy group at the C15’ position, a three step sequence involving protection and deprotection of the primary hydroxy group was required. The resulting alcohol **112** was oxidized under Swern conditions [166] and the resulting aldehyde was submitted to a Wittig reaction with ethyl (triphenylphosphoranylidene)propionate. The subsequent reduction of the ensuing ester with DIBAL-H and oxidation with MnO_2_ delivered α,β-unsaturated aldehyde **113**.

At this point, it is worth mentioning that an initial attempt to elaborate the entire pentaene backbone iteratively was hampered by the limited stability of the doubly unsaturated aldehyde obtained from **113** after another two-carbon elongation cycle. The LDA-mediated HWE reaction of **113** with phoshonate **109**, however, proceeded smoothly to provide the fully protected pentaenoate **114** in a longest linear sequence of 10 steps (19 in total) and 20% overall yield from benzylated 4,6-dideoxy-D-glucose **110**. Compound **114** was obtained with a *Z*-Δ^4’,5’^/*E*-Δ^4’,5’^-ratio of 3:2 as demonstrated by NOESY-NMR studies. In accordance with later findings [43,122], the authors reported a slow re-equilibration of the C4’–C5’ double bond isomers after separation by HPLC on a chiral stationary phase. However, no conclusions were drawn at that stage with regard to the configuation of the C12’, C13’ and C15’ stereocenters. Although the utility of Gurjar and Cherian’s work is confined by the limited availability of methods to selectively remove the benzyl ether protecting groups in the presence of the sensitive pentaenoate system and the stereochemistry at the C15’ position that would require to start from expensive L-sugars [196] to furnish the desired epimer, as already alluded to above, their approach was adopted by other groups in their strategies towards mycolactones A/B (vide infra).

**Scheme 14:**
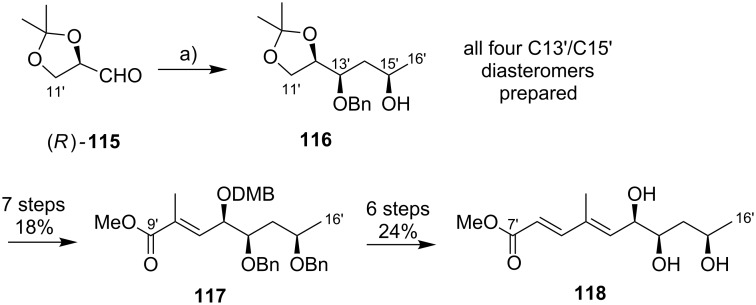
Kishi’s synthesis of model compounds for elucidating the stereochemistry of the C7’–C16’ fragment of the mycolactone A/B pentaenoate side chain. Reagents and conditions: a) (i) (*R*,*R*)- and (*S*,*S*)-diisopropyl tartrate-modified allylboronate, 4 Å molecular sieves, toluene, −78 °C, then NaBH_4_, EtOH, −78 °C; (ii) NaH, BnBr, DMF, 0 °C to rt, 88%; (iii) OsO_4_, NMO, DABCO, THF/H_2_O 10:1, rt; (iv) Pb(OAc)_4_, benzene, rt; (v) MeLi, CuI, −20 °C, 83% (3 steps), 1:1 mixture of diastereomers.

Kishi’s synthesis of the mycolactone A/B pentaenoate side chain incorporated Gurjar and Cherian’s approach towards triene **109** (Scheme 12) with the minor modification of using the methyl ester instead of the ethyl ester at the C1’ position [43]. For the eastern C9’–C16’ fragment, however, a different strategy was chosen. In Kishi’s earlier studies, model compounds were prepared to elucidate the stereochemistry at C12’, C13’ and C15’ by NMR spectroscopy [40]. To enable the determination of the relative configuration at those three proximal stereocenters, a route permitting the synthesis of all possible stereoisomers at the C13’ and the C15’ position was chosen, while keeping the configuration at C12’ invariable. The absolute stereochemistry would then be deduced by comparison of NMR spectra of the model compounds with the natural material in chiral solvents. The synthesis of the model compounds started from D-glyceraldehyde acetonide ((*R*)-**115**), which was subjected to Roush allylation [197] with either (*R*,*R*)- or (*S*,*S*)-diisopropyl tartrate-modified allylboronates [176] to separately obtain two diastereomeric homoallylic alcohols (Scheme 14).

After benzyl protection and oxidative cleavage of the double bond, an unselective methyl cuprate addition gave access to a diastereomeric mixture of **116** that could be separated after hydrogenolytic benzyl cleavage. In seven more steps, including a Wittig olefination and several redox and protecting group manipulations, **116** was transformed into α,β-unsaturated ester **117**, and six more steps were required to obtain the four diastereomeric C7’–C16’ model dienes exemplified by **118**. As discussed above, comparison of ^1^H NMR shifts revealed the relative *syn*,*syn*-relationship of the C12’, C13’ and C15’ hydroxy groups and differential ^1^H NMR profiles in (*R*)- and (*S*)-*N*,α-dimethylbenzylamine (DMBA) unveiled the C12’/C13’/C15’ configuration of **118** to be the opposite of natural mycolactone A/B.

**Scheme 15:**
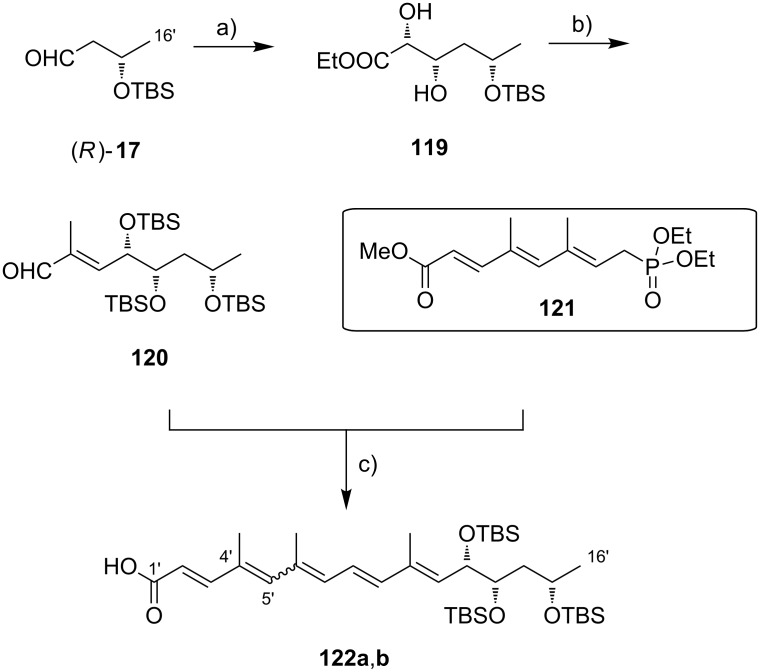
Kishi’s synthesis of the mycolactone A/B pentaenoate side chain. (a) (i) NaH, (EtO)_2_P(O)CH_2_CO_2_Et, THF, rt, 64%; (ii) AD-mix-α, MeSO_2_NH_2_, 1:1 *t*-BuOH/H_2_O, 0 °C, 70%, dr 3.8:1; (b) (i) TBSOTf, 2,6-lutidine, CH_2_Cl_2_, 0 °C, 99%; (ii) DIBAL-H, CH_2_Cl_2_, −78 °C, 89%; (iii) SO_3_·pyridine, DIPEA, 3:2 CH_2_Cl_2_/DMSO, rt; (iv) Ph_3_P=C(Me)CO_2_Et, toluene, 110 °C, 83% (2 steps); (v) DIBAL-H, CH_2_Cl_2_, −78 °C, then separation of diastereomers by flash chromatography; major isomer: 57%; minor isomer: 15%; (vi) SO_3_·pyridine, DIPEA, 3:2 CH_2_Cl_2_/DMSO, rt, quant.; c) (i) LDA, THF, −78 °C to rt, 94% (*E*-Δ^4’,5’^/*Z*-Δ^4’,5’^/other isomers 73:17:10); (e) LiOH, THF/MeOH/H_2_O 4:1:1, rt, quant. (*Z*-Δ^4’,5’^/*E*-Δ^4’,5’^ 3:2 + minor isomers).

Although the route used to prepare the model compounds could have been used to prepare aldehyde **120** (Scheme 15) required for assembly of the lower side chain of natural mycolactone A/B by HWE olefination, Kishi and co-workers pursued an alternative strategy. Even though, not commented on in their report, obvious reasons against the previous strategy include its length and the lack of stereocontrol during the desymmetrization of the C15’ atom. Kishi’s improved approach commenced with a Wittig reaction to elongate literature known aldehyde (*R*)-**17** (Scheme 15).

The α,β-unsaturated ester obtained was submitted to asymmetric Sharpless dihydroxylation [198] with AD-mix-α [199], which proceeded with a moderate 3.8:1 diastereoselectivity in favor of the desired diastereomer **119**. The undesired diastereomers, however, could be separated chromatographically at a later stage of the synthesis. TBS protection of both hydroxy groups followed by a five-step reduction/oxidation/Wittig reaction sequence furnished key aldehyde **120**. The latter was connected to phosphonate **121** under Gurjar and Cherian’s HWE conditions, furnishing full length pentaenoate **122a**,**b**.

Photochemical equilibration gave an inseparable 35:52:4:5 mixture of the all-*E,* the *Z*-Δ^4’,5’^, the *Z*-Δ^6’,7^ and the *Z*-Δ^4’,5’^/*Z*-Δ^6’,7’^ isomers, containing, in addition, 3% of a fifth isomer. After ester hydrolysis, the two major geometric isomers could be separated as a 3:2 mixture of the *Z*-Δ^4’,5’^ and the *E*-Δ^4’,5’^ isomer. The mycolactone side chain was thus obtained in 10 steps and 18% overall yield from aldehyde (*R*)-**17**.

Endeavors towards the synthesis of the polyunsaturated mycolactone A/B side chain were subsequently reported by the groups of Feringa and Minnaard [196]. Although they did not ultimately complete the synthesis, Feringa and Minnaard established a convenient access towards intermediates with the correctly configured C12’, C13’ and C15’ stereocenters by using readily available α*-*D-glucopyranoside or α*-*L*-*rhamnopyranoside as starting materials. Furthermore, the preparation of several key precursors that might be useful for the assembly of (modified) mycolactone A/B side chains was reported, although the connection of these fragments could not be successfully executed at the time. Due to space limitations, only the most significant aspects of this work will be highlighted here.

**Scheme 16:**
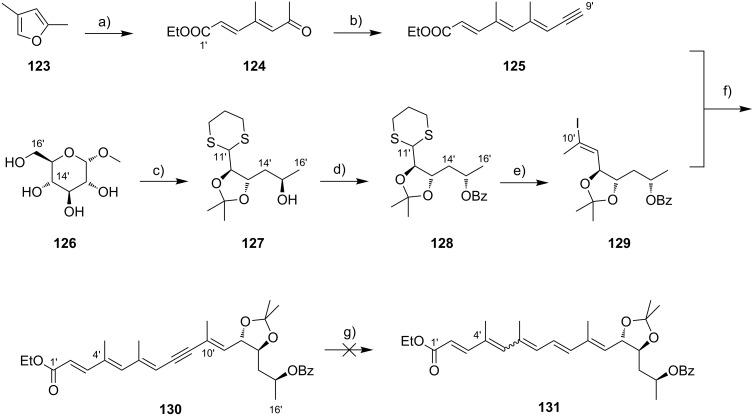
Feringa and Minnaard's incomplete synthesis of mycolactone A/B pentaenoate side chain. Reagents and conditions: a) (i) Rh_2_(OAc)_4_ (0.4 mol %), CH_2_Cl_2_, ethyl diazoacetate, rt; (ii) I_2_, CH_2_Cl_2_, rt; b) (i) TMS–C≡C–CH_2_–P(O)(OEt)_2_, *n*-BuLi, THF, 0 °C to rt, 61%; (ii) TBAF, THF, EtOAc, 0 °C, 80%; c) (i) SO_2_Cl_2_, pyridine, CHCl_3_, −78 °C to 50 °C, 56%; (ii) Bu_3_SnH, AIBN, toluene, reflux, 89%; (iii) 1,3-propanedithiol, conc. HCl, 87%; (iv) acetone, CuSO_4_, H_2_SO_4_, 95%; d) PPh_3_, BzOH, DEAD, THF, 82%; e) (i) MeI, 2,4,6-collidine, acetone, H_2_O, reflux, 89%; (ii) PPh_3_, CBr_4_, CH_2_Cl_2_, 0 °C to rt, 72%; (iii) LDA, THF, −78 °C, 88%; (iv) LDA, HMPA, MeI, THF, −78 °C to −10 °C, 88%; (v) Pd(PPh_3_)_2_Cl_2_, Bu_3_SnH, pentane, 63%; (vi) CH_2_Cl_2_, I_2_, −78 °C to rt, 99%; f) Pd(PPh_3_)_4_, CuI, iPrNH_2_, 94%; g) H_2_, Lindlar catalyst, hexanes, EtOAc, quinoline; or Zn, Cu(OAc)_2_·H_2_O, AgNO_3_, H_2_O, MeOH; or H_2_, THF, Elsevier catalyst; or Ni(OAc)_2_·4H_2_O, EtOH, H_2_, hydrazine, NaBH_4_.

The preparation of the western C1’–C9’ fragment started from known 2,4-dimethylfuran (**123**) [200], which was transformed into keto ester **124** by a rhodium-catalyzed reaction with ethyl diazoacetate [201] (Scheme 16).

A two-carbon elongation was then performed by HWE reaction with TMS-protected diethyl ethynylmethyl phosphonate, and the subsequent TMS cleavage afforded alkyne **125**. The synthesis of the eastern fragment started from α*-*D-methyl glucopyranoside (**126**), which was converted into partially protected triol **127** by selective chlorination of the C14’ and the C16’ positions and consecutive reductive removal of the chlorine atoms as the key steps. The configuration at the C15’ position was subsequently inverted under Mitsunobu conditions [202] furnishing benzoate ester **128**, which is also a key intermediate in the Altmann synthesis of the mycolactone A/B pentaenoate chain. After dithiane cleavage, the resulting aldehyde was subjected to a Corey–Fuchs reaction [146]/methylation sequence to furnish a methylalkyne that underwent palladium-catalyzed hydrostannylation [203] with moderate regioselectivity (6.3:1 ratio in favor of the desired isomer). Tin–iodine exchange finally delivered vinyl iodide **129** in 16% yield over 10 steps. An alternative synthesis of **129** starting from α*-*L*-*rhamnopyranoside proved less cost-efficient and concise. Vinyl iodide **129** was reacted with terminal alkyne **125** in a Sonogashira cross-coupling reaction [141] to produce the full length C1’–C16’ fragment **130**. Unfortunately, all conditions screened to selectively reduce the internal triple bond in **130** (e.g., hydrogenation with Lindlar catalyst [204] or Elsevier catalyst [205], reduction with Ni(OAc)_2_/NaBH_4_ [206] or Zn(Cu/Ag) [207]) failed to provide pentaenoate **131**. Moreover, all attempts to convert terminal alkyne **125** into the corresponding *E*-vinyl iodide or stannane that might have been used to assemble the C1’–C16’ fragment in a palladium-catalyzed C(sp^2^)–C(sp^2^) cross-coupling reaction were not successful nor was an alternative strategy with a retrosynthetic disconnection at the C7’–C8’ double bond.

**Scheme 17:**
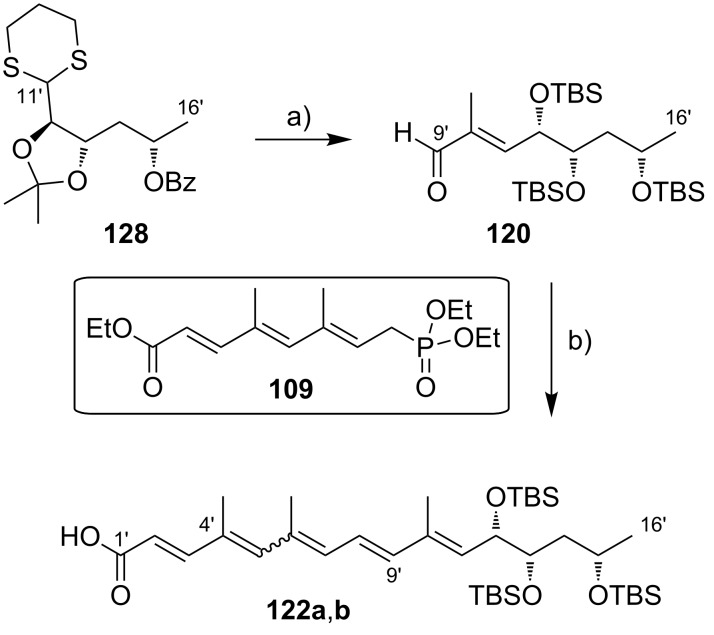
Altmann’s approach towards the mycolactone A/B pentaenoate side chain. Reagents and conditions: a) (i) NaH, MeOH, rt, 95%; (ii) cat. TFA, AcOH, H_2_O, rt, quant.; (iii) TBSCl, imidazole, DMF, 70 °C, 96%; (iv) MeI, 2,4,6-collidine, acetone, H_2_O, reflux, 97%; (v) Ph_3_P=C(Me)COOEt, benzene, reflux, 93%; (vi) DIBAL-H, CH_2_Cl_2_, −78 °C, 91%; (vii) SO_3_-pyridine, DIPEA, CH_2_Cl_2_/DMSO, rt, 97%; b) (i) **109**, LDA, THF, −78 °C to rt, 90%; (ii) LiOH, THF/H_2_O/MeOH (4:1:1), rt, 99%.

As noted above, our own group used a hybrid approach that combined access to the C1’–C8’ fragment according to Gurjar and Cherian with the synthesis of the chiral C11’–C16’ fragment according to Feringa and Minnaard (Scheme 17) [178]. To this end, intermediate **128** from the Feringa/Minnaard synthesis [196] was partially deprotected and reprotected and then elongated to **120** by a Wittig/reduction/oxidation sequence. HWE reaction of **120** with **109** under the conditions elaborated by Gurjar and Cherian gave rise to the full length C1’–C16’ fragment; alkaline saponification finally furnished acid **122a**,**b** in 15 steps (longest linear sequence) and 18% overall yield from α*-*D-methyl glucopyranoside.

A distinct approach selectively providing both the mycolactone A and the mycolactone B pentaenoate chain was followed by the Negishi group [37]. In analogy to their synthesis of the mycolactone core, this strategy was largely driven by the desire to demonstrate the synthetic utility of their (hydro/carbo)metalation and cross-coupling methodologies. In a communication in 2006, Negishi and co-workers reported the stereoselective synthesis of both of the above mycolactone side chains [208]. However, it turned out later that the protecting group strategy chosen in this initial work was not appropriate for the late stage global deprotection envisaged in the total synthesis of mycolactones A and B. Therefore, minor adjustments (replacement of the C12’ MOM ether by a TBS ether) were made in the context of the total synthesis. Since the syntheses from both reports are virtually identical, only the 2nd generation approach will be discussed here.

**Scheme 18:**
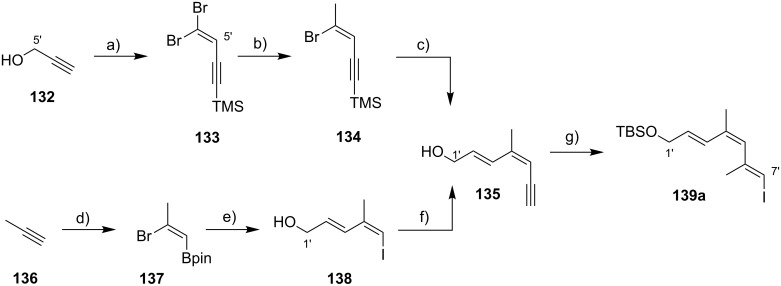
Negishi’s access to the C1’–C7’ fragment of mycolactone A. Reagents and conditions: a) (i) *n*-BuLi, TMSCl, then HCl; (ii) (COCl)_2_, DMSO; (iii) CBr_4_, PPh_3_, Zn, 90% (3 steps); b) Me_2_Zn, Pd(dpePhos)Cl_2_ (5 mol %), DMF/THF 1:1, rt, 70%; c) (i) [*trans*-TBSO–CH_2_C=C–ZnBr], Pd(dpePhos)Cl_2_ (5 mol %), THF/DMF 1:1, rt to 45 °C; (ii) TBAF, THF, rt, 66% (2 steps); d) (i) BBr_3_, CH_2_Cl_2_; (ii) pinacol; e) (i) [*trans*-iBu_2_Al–OCH_2_C=C–ZnBr], PEPPSI (1 mol %); (ii) I_2_, NaOH, THF/H_2_O, 77% (2 steps); f) Et_2_Zn, then (HC≡C)_2_–Zn, Pd(*t*-Bu_3_P)_2_ (0.5 mol %), 94%; g) (i) AlMe_3_, Cp_2_ZrCl_2_, CH_2_Cl_2_, −78 °C to rt, then I_2_, THF, −78 °C; (ii) TBSCl, imidazole, DMF, rt, 65% (2 steps).

The Negishi group provided two different synthetic pathways to prepare the C1’–C7’ fragment with a *Z*-configured C4’–C5’-double bond (Scheme 18). The first started from propargyl alcohol (**132**) that was converted into the geminal dibromoolefin **133** [209] by a sequence of TMS protection of the terminal alkyne moiety, Swern oxidation and dibromoolefination of the ensuing aldehyde according to Corey–Fuchs. A highly *E*-selective palladium-catalyzed methylation with ZnMe_2_ yielded *Z-*bromoolefin **134** that was converted into ynediene **135** by another Negishi-type cross-coupling reaction with (*E*)-(3-((*tert*-butyldimethylsilyl)oxy)prop-1-en-1-yl)zinc bromide followed by global silyl deprotection. Compound **135** was thus obtained in 42% yield over 6 steps.

An alternative route to obtain **135** departed from propyne (**136**) [210] which underwent bromoborylation to **137**, which served as the precursor for a Negishi alkenylation and alkynylation reaction, respectively. Of note, this approach relied on a transient protection of the C1’ hydroxy group as a diisobutylaluminum complex during cross-coupling. Intermediate **135** was obtained in only three steps and 72% overall yield thereby clearly outcompeting the approach departing from propargylic alcohol both in terms of step count and efficiency. Finally, transformation to *E*,*Z*,*E*-configured trienyl iodide **139a** was achieved in two steps and 65% yield by means of a zirconium-mediated carboalumination/iodination sequence followed by TBS protection [211].

**Scheme 19:**
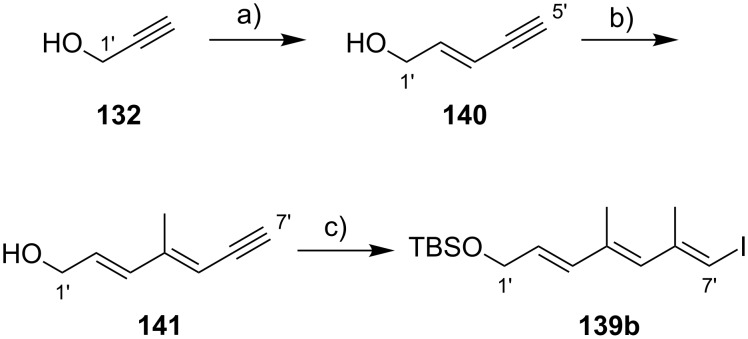
Negishi’s approach to the C1’–C7’ fragment of mycolactone B. Reagents and conditions: a) (i) DIBAL-H, THF, 0 °C, then DIBAL-H, Cp_2_ZrCl_2,_ THF, 0 °C to rt, then I_2_, THF, −78 °C; (ii) Et_2_Zn; then (HC≡C)_2_–Zn, Pd(dpePhos)Cl_2_ (5 mol %), THF, 0 °C to rt, 58% (2 steps); b) (i) AlMe_3_/Cp_2_ZrCl_2_, CH_2_Cl_2_, −78 °C to rt, then I_2_, THF, −78 °C; (ii) (HC≡C)_2_–Zn, Pd(dpePhos)Cl_2_ (5 mol %), THF, 0 °C to rt, 62% (2 steps); c) (i) AlMe_3_/Cp_2_ZrCl_2_, CH_2_Cl_2_, −78 °C to rt, then I_2_, THF, −78 °C; (ii) TBSCl, imidazole, DMF, rt, 63% (2 steps).

The C1’–C7’ fragment with an *E*-configured C4’–C5’-double bond was also prepared from propargyl alcohol (**132**, Scheme 19). Transient protection of the C1’ hydroxy group with DIBAL-H followed by hydrozirconation with in situ-generated Schwartz reagent [212] and quenching with iodine yielded an (*E*)*-*vinyl iodide, that was further processed into enyne **140** by Negishi cross-coupling with bis(ethynyl)zinc. Again, transient hydroxy protection was employed with diethylzinc as the blocking agent. A second zirconium-mediated carboalumination/iodination/cross-coupling sequence then furnished terminal alkyne **141** that was eventually transformed into all-(*E*) vinyl iodide **139b**, again by carboalumination/iodination.

**Scheme 20:**
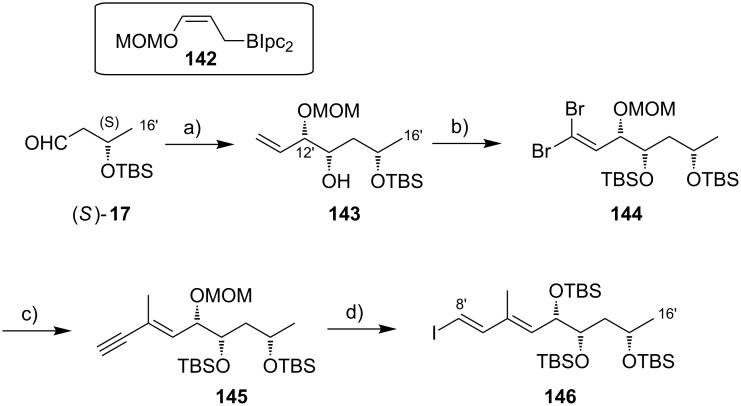
Negishi’s synthesis of the C8’–C16’ fragment of mycolactone A/B. Reagents and conditions: a) **142**, BF_3_·Et_2_O, Et_2_O, −90 °C to 0 °C, then H_2_O_2_, aq NaHCO_3_, 91%, dr 94:4; b) (i) TBSOTf, 2,6-lutidine, CH_2_Cl_2_, 0 °C; (ii) OsO_4_ (1 mol %), NMO, THF, H_2_O, rt; (iii) NaIO_4_, THF, H_2_O, rt, 95% (3 steps); (iv) PPh_3_, CBr_4_, 2,6-lutidine, CH_2_Cl_2_, 0 °C, 96%; c) (i) TMS–C≡C–ZnBr, Pd(dpePhos)Cl_2_ (5 mol %), THF, 0 °C; (ii) Me_2_Zn, Pd(*t-*Bu_3_P)_2_ (2 mol %), THF, rt; (iii) K_2_CO_3_, MeOH, rt, 61% (3 steps); d) (i) HCl (3 M in H_2_O), MeOH, 55 °C; (ii) TBSOTf, 2,6-lutidine, CH_2_Cl_2_, rt; (iii) Cp_2_Zr(H)Cl, THF, rt, then I_2_, −78 °C, 55% (3 steps).

Negishi and co-workers again decided for an independent strategy, when it came to the synthesis of the eastern C8’–C16’ fragment. Aldehyde (*S*)-**17** was prepared as previously and submitted to a variant of the Brown allylation [213] employing (+)-(*Z*)*-*MOM-OCH=CHCH_2_B(Ipc)_2_ (**142**), thus enabling the simultaneous installation of the stereocenters at C12’ and C13’ (Scheme 20). Although this reaction was highly selective, it came at the cost of requiring a MOM ether protecting group which necessitated further protecting group manipulations, in order to enable late stage global deprotection. Alkene **143** was cleaved under Upjohn/Lemieux–Johnson [147–148] conditions and conversion of the resulting aldehyde into the geminal vinyl dibromide **144** was achieved by employing the Corey–Fuchs protocol. The more reactive *E*-bromo substituent underwent selective palladium-mediated alkynylation with TMS-ethynylzinc bromide, which was followed by another Negishi-type cross-coupling reaction between the *Z*-bromide and dimethylzinc to furnish enyne **145**. After several protecting group manipulations, a final hydrozirconation/iodination reaction then yielded key dienyl iodide **146** in 28% yield over 11 steps (longest linear sequence from (*S*)-**17**). At this point, it is worth mentioning that dienyl iodide **146** was also prepared using a different route in the course of our own studies and we found this material to be relatively unstable even at −18 °C, thus hampering the storage of this key intermediate (Gehringer & Altmann, unpublished).

**Scheme 21:**
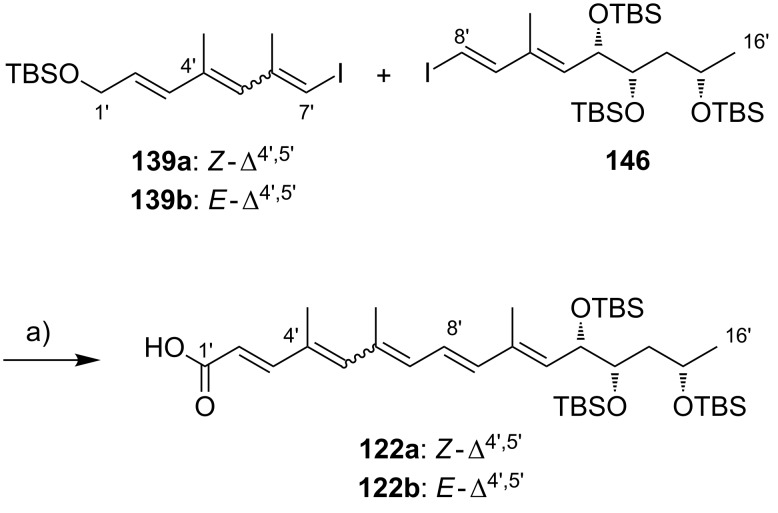
Negishi’s assembly of the mycolactone A and B pentaenoate side chains. Reagents and conditions: a) (i) **139a** or **139b**, *t*-BuLi, then dry ZnBr_2_, Et_2_O, THF, −78°C to rt, then **146**, Pd(dpePhos)Cl_2_ (5 mol %), DMF, rt; (ii) TBAF, THF, 0 °C, 61% and 65% (2 steps); (iii) DMP, NaHCO_3_, CH_2_Cl_2_, rt; (iv) NaClO_2_, NaH_2_PO_4_, 2-methyl-2-butene *t-*BuOH/H_2_O 2:1, rt, **122a**: 73%, **122b**: 76% (2 steps).

With the C1’–C7’ and the C8’–C16’ fragments in hand, the subsequent assembly was carried out in parallel for the respective precursors of mycolactone A and B. To assemble the polyunsaturated side chain, the trienyl iodides **139a** and **139b** were lithiated with *t*-BuLi. After transmetallation, the corresponding alkenylzinc intermediates were subjected to Pd-mediated Negishi coupling with dienyl iodide **146** (Scheme 21). After selective unmasking of the primary hydroxy group at C1’, a Dess–Martin/Pinnick–Kraus oxidation sequence afforded the highly pure (≥98%) side chain acids of mycolactone A (**122a**) and B (**122b**) in 15 steps (longest linear sequence) from (*S*)-**17** in 12% and 14% overall yield, respectively.

**Scheme 22:**
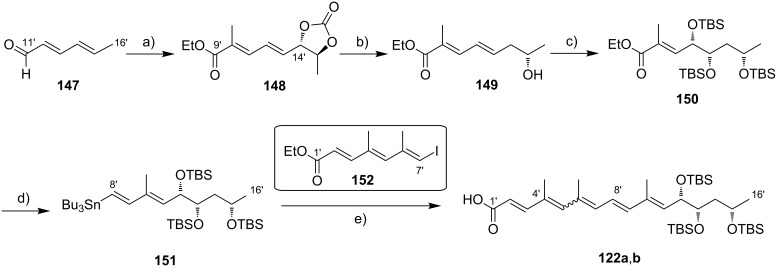
Blanchard’s approach to the mycolactone A/B pentaenoate side chain. a) (i) Ph_3_P=C(Me)COOEt, CH_2_Cl_2_, rt, 99%; (ii) AD-mix α, K_2_OsO_4_·2H_2_O (0.6 mol %), MeSO_2_NH_2_, *t*-BuOH, H_2_O, 0 °C, 70%, 86% ee; (iii) triphosgene, pyridine, CH_2_Cl_2_, rt, 79%; b) Pd_2_(dba)_3_·CHCl_3_ (0.5 mol %), HCO_2_H, Et_3_N, THF, rt, 63%; c) (i) TBSCl, imidazole, DMAP, DMF, rt, 93%; (ii) AD-mix α, K_2_OsO_4_·2H_2_O (2 mol %), MeSO_2_NH_2_, *t*-BuOH, H_2_O, 0 °C, 70%; (iii) TBSCl, imidazole, DMAP, DMF, rt, 83%; d) (i) DIBAL-H, CH_2_Cl_2_, −78 °C, 97%; (ii) MnO_2_, CH_2_Cl_2_, 94%; (iii) CrCl_2_, *n*-Bu_3_SnCHBr_2_, LiI, THF/DMF 20:1, rt; e) (i) **152**, CuTC, Ph_2_P(O)OBu_4_N, NMP, rt, 48% (2 steps); (ii) LiOH, THF, H_2_O, 92%; (iii) *hν*, acetone, rt, quant.

Finally, a very distinct approach to the polyunsaturated mycolactone A/B side chain was established by Blanchard and co-workers. With the goal of developing a diverted total synthesis of C8-desmethylmycolactone analogs for SAR studies, the Blanchard group required a general strategy that would give access to different stereoisomers of the lower side chain [92]. Therefore, they adopted a methodology developed by O’Doherty [214] that involves catalytic asymmetric oxidation and subsequent reductive defunctionalization reactions to construct all three stereocenters. The linkage of the C1’–C7’ and the C8’–C16’ fragments relied on a Stille-type coupling reaction. Starting from readily available *trans*-hexadienal (**147**) (which corresponds to the C11’–C16’ segment), a Wittig two-carbon elongation followed by stereoselective Sharpless dihydroxylation (86% ee) of the most electron-rich double bond and subsequent reaction with triphosgene furnished cyclic carbonate **148** (Scheme 22). The C14’ position was then defunctionalized to give alcohol **149** by palladium-catalyzed allylic reduction using triethylammonium formate as the hydride donor [214]. A second Sharpless dihydroxylation and subsequent TBS protection afforded fully protected triol **150** with the correctly configured stereocenters at C12’, C13’, and C15’ in place. Ester reduction and allylic oxidation with MnO_2_ followed by chromium-mediated one-carbon elongation with Bu_3_SnCHBr_2_ [215] led to dienyl stannane **151**, the precursor for the Stille cross-coupling.

The partner for this coupling reaction, vinyl iodide **152**, was obtained from known (*E*)-3-iodo-2-methylprop-2-en-1-ol [216] by two Wittig elongation cycles. Instead of using traditional Stille conditions, Blanchard and co-workers relied on the palladium-free copper(I) thiophene-2-carboxylate (CuTC)-promoted variant developed by Allred and Liebeskind [217]. Coupling proceeded rapidly at ambient temperature in the presence of tetra-*n*-butylammonium diphenylphosphinate as tin scavenger [218], but only moderate yields were obtained, which somewhat limits the overall efficiency of the synthesis. Final ester hydrolysis and photochemical equilibration furnished the mycolactone A/B pentaenoate side chain acid in 12 steps (longest linear sequence) and 7.4% overall yield from *trans*-hexadienal (**147**). Ultimately, a set of 4 stereoisomers (vide infra) was prepared via this route (as pairs of *E*/*Z* isomers at C4',C5', including **122a**,**b**).

**III.2.2 Synthesis of the polyunsaturated side chains of other natural mycolactones:** The most extensive contributions to the synthesis of the polyunsaturated side chains of other natural mycolactones were again made by the Kishi laboratory. After having completed the total synthesis of mycolactone A/B, Kishi and co-workers devised strategies for the synthesis of mycolactones C, E, F, S1 and S2 and the photochemical decomposition products of mycolactone A/B (“photo-mycolactones”). Contributions from other groups include the approach to the mycolactone E side chain developed by Wang and Dai and Blanchard’s synthesis of the mycolactone C side chain, as well as our own unpublished work on the latter.

**Scheme 23:**
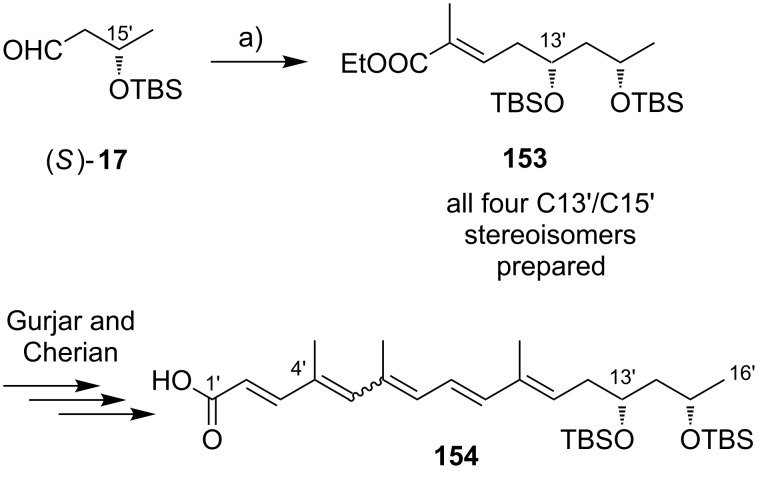
Kishi’s approach to the mycolactone C pentaenoate side chain exemplified for the 13’*R*,15’*S*-isomer **154**. Reagents and conditions: a) (i) (+)-Ipc_2_BOMe, allylmagnesium bromide, Et_2_O, −78 °C, 67%, dr 8:1; (ii) TBSCl, imidazole, DMF, rt; (iii) O_3_, CH_2_Cl_2_, −78 °C, then PPh_3_; (iv) Ph_3_P=C(Me)COOEt, toluene, 110 °C, 84% (3 steps).

**III.2.2.1. Synthesis of the pentaenoate side chain of mycolactone C:** At the time when Kishi and co-workers initiated their work on mycolactone C, only a gross structure had been proposed for the compound by the Small [47] and the Leadlay groups [52] (vide supra). Having a suitable route to the mycolactone core in hand, a flexible approach enabling the synthesis of all four possible stereoisomers of the proposed 1,3-diol motif in the pentaenoate side chain was required for Kishi to synthesize mycolactone C and establish its exact structure. Kishi’s work departed from the two enantiomers of TBS-protected 3-hydroxybutyraldehyde **17**. The second stereocenter was introduced by Brown asymmetric allylation with allylmagnesium bromide [219] in the presence of (+)- or (−)-Ipc_2_BOMe, respectively (Scheme 23) [53]. After TBS-protection of the resulting secondary alcohols and ozonolysis of the homoallylic double bond, a two-carbon chain extension was performed by Wittig chemistry to obtain α,β-unsaturated ester **153** (and all of the corresponding stereoisomers). The ester was further processed according to Gurjar and Cherian’s protocol to deliver all four C13’/C15’ stereoisomers of the putative mycolactone C side chain as 1:1 mixtures of *Z*-Δ^4’,5’^ and *E*-Δ^4’,5’^-isomers. As an example, Scheme 23 shows that the 13’*R*,15’*S*-isomer **154** was obtained in 48% overall yield for the 8-step sequence from (*S*)-3-hydroxybutyraldehyde ((*S*)-**17**).

**Scheme 24:**
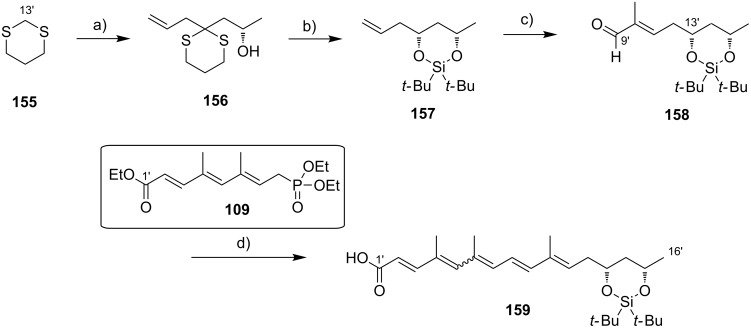
Altmann’s (unpublished) synthesis of the mycolactone C pentaenoate side chain. Reagents and conditions: a) (i) *n*-BuLi, THF, −78 °C, then allyl bromide, −78 °C to rt, 97%; (ii) *n*-BuLi, THF, −10 °C, then (*S*)-propylene oxide, −10 °C, 74%; b) (i) I_2_, NaHCO_3_, MeCN/H_2_O 2:1, 0 °C, 85%; (ii) Et_2_BOMe, NaBH_4_, THF, MeOH, −78 °C, 71%, dr 17:1; (iii) *t*-Bu_2_Si(OTf)_2_, pyridine, CH_2_Cl_2_, 0 °C, 85%; c) (i) O_3_, CH_2_Cl_2_, −78 °C, then PPh_3_, 76%; (ii) Ph_3_P=C(Me)CHO, benzene, reflux, 77%; d) (i) **109**, LDA, THF, −78 °C to rt, 70%, (*E*-Δ^4’,5’^/*Z*-Δ^4’,5’^ 4:1); (ii) LiOH, THF/H_2_O/MeOH 4:1:1, rt, 95% (*E*-Δ^4’,5’^/*Z*-Δ^4’,5’^/minor isomers 72:22:6).

An alternative, as yet unpublished approach to the mycolactone C fatty acid side chain was recently developed in our own laboratories [220]. The synthesis started with the allylation of 1,3-dithiane (**155**) with allyl bromide (Scheme 24). Deprotonation of the resulting allylated dithiane and quenching with (*S*)-propylene oxide ((*S*)-**38**) yielded (*S*)-configured alcohol **156**.

Unmasking the keto group with iodine under slightly basic conditions followed by a chelation-controlled 1,3-*syn* reduction with NaBH_4_ in the presence of Et_2_BOMe [221] provided a 1,3-diol that was converted into the cyclic di-*tert*-butylsilyl ether **157**. Cleavage of the double bond by ozonolysis followed by a two-carbon elongation via Wittig olefination with 2-(triphenylphosphoranylidene)propanal yielded aldehyde **158**, which was to be submitted to HWE reaction with phosphonate **109**. The HWE reaction, however, proved to be more difficult than for the analogous step in the synthesis of the mycolactone A/B side chain. After some experimentation, it was found that a two-fold excess of deprotonated phosphonate **109** was necessary to consume most (>80%) of the aldehyde **158**. Fortunately, the starting materials could be recovered, yielding 70% of the ethyl pentaenoate as an inseparable 4:1 mixture of the *E*-Δ^4’,5’^ and the *Z*-Δ^4’,5’^-isomers along with 4% of a minor isomer. The ethyl ester smoothly underwent saponification with LiOH to yield acid **159** in 95% yield as a 72:22:6 mixture of the *E*-Δ^4’,5’^-isomer, the *Z*-Δ^4’,5^-isomer and other minor isomers, respectively. This product was obtained from 1,3-dithiane (**155**) in 7 steps and 14% overall yield.

**Scheme 25:**
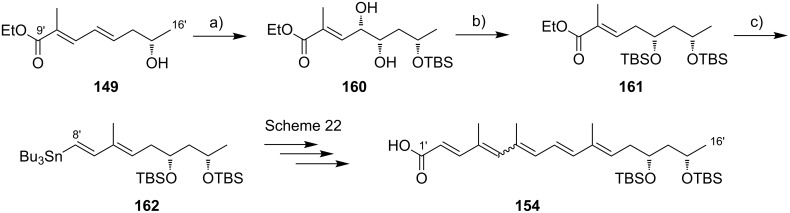
Blanchard’s synthesis of the mycolactone C pentaenoate side chain. Reagents and conditions: a) (i) TBSCl, imidazole, DMAP, DMF, rt, 93%; (ii) AD-mix α, K_2_OsO_4_·2H_2_O (2 mol %), MeSO_2_NH_2_, *t*-BuOH/H_2_O, 0 °C, 70%; b) (i) triphosgene, pyridine, CH_2_Cl_2_, 0 °C, 84%; (ii) Pd_2_(dba)_3_·CHCl_3_ (0.4 mol %), HCO_2_H, Et_3_N, THF, rt, 95%; (iii) TBSCl, imidazole, DMAP, DMF, rt, 87%; c) (i) DIBAL-H, CH_2_Cl_2_, 0 °C, 81%; (ii) MnO_2_, CH_2_Cl_2_, reflux, 93%; (iii) CrCl_2_, CHI_3_, THF, rt, 59%; (iv) *n-*BuLi, Et_2_O, −78 °C, then *n-*Bu_3_SnCl, −78 °C to rt.

Yet an alternative approach towards the mycolactone C side chain was reported by Blanchard and co-workers in the context of their work on C8-desmethylmycolactone analogs [92]. The synthesis relied on the same logic as their synthesis of the mycolactone A/B side chain (cf. Scheme 22). Briefly, intermediate **149** was protected and stereoselectively dihydroxylated with AD-mix α at the γ,δ-double bond (Scheme 25). The resulting diol **160** was converted into the corresponding cyclic carbonate, defunctionalized in the allylic position with triethylammonium formate/palladium(0) and TBS-protected to provide ester **161**. Transformation of **161** into vinylstannane **162** was achieved by reduction to the corresponding aldehyde followed by Takai-olefination [222], lithiation and quenching of the vinyllithium intermediate with tributyltin chloride. Intermediate **162** was further processed according to Scheme 22 to obtain the complete lower side chain acid **154** in 15 steps (longest linear sequence) and 0.8% overall yield from *trans*-hexadienal.

**Scheme 26:**
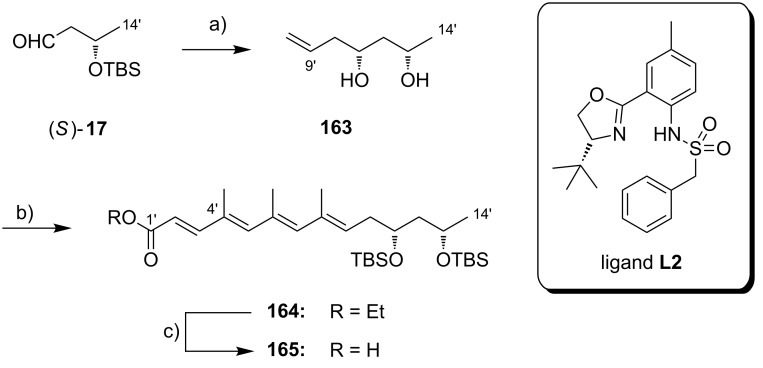
Kishi’s synthesis of the tetraenoate side chain of mycolactone F exemplified by enantiomer **165**. Reagents and conditions: a) (i) CrCl_3_·3THF, ligand **L2**, Mn, Et_3_N, THF, then 2,6-lutidine, allyl bromide, Cp_2_ZrCl_2_, rt, 83%, dr 14:1; (ii) TBAF, 84% after separation of the minor diastereomer; b) (i) TBSCl, imidazole, DMF, rt, quant.; (ii) O_3_, CH_2_Cl_2_, −78 °C, PPh_3_, 83%; (iii) (EtO)_2_P(O)CH(Me)COOEt, *n*-BuLi, THF, 0 °C; (iv) DIBAL-H, CH_2_Cl_2_, −78 °C; (v) MnO_2_, CH_2_Cl_2_, 72% (3 steps); (vi) (EtO)_2_P(O)CH(Me)COOEt, *n*-BuLi, THF, 0 °C; (vii) DIBAL-H, CH_2_Cl_2_, −78°C; (viii) MnO_2_, CH_2_Cl_2_, 62% (3 steps); (ix)–(xi) repeat steps vi–viii, 36% (3 steps); (xii) (EtO)_2_P(O)CHCOOEt, *n*-BuLi, THF, 0 °C, 90%; c) LiOH, THF/MeOH/H_2_O 4:1:1, rt, 89%.

**III.2.2.2. Synthesis of the tetraenoate side chain of mycolactone F:** Kishi and co-workers have also addressed the total synthesis of mycolactone F (**8**) [59], another mycolactone congener, whose gross structure had been inferred from mass spectrometry data, while the relative and absolute configuration of the lower side chain could not be assigned. The Kishi group assumed a *syn*-relationship of the 1,3-diol moiety in analogy to the structures that had been previously established for other mycolactone variants [40,43,53]. Consequently, only the two enantiomers with an *S*,*R* or *R*,*S*-configuration, respectively, at the C11’- and C13’-positions were to be prepared (exemplified by **163**, Scheme 26). Again, the two enantiomers of aldehyde **17** served as starting points for the syntheses, which were identical for both enantiomers. Aldehyde **17** was reacted with allyl bromide in an asymmetric variant of the Nozaki–Hiyama–Kishi coupling reaction [223–224] using ligand **L2**, which had previously been developed by the Kishi group [225]. Interestingly, a Cr/Zr/Mn system was used to promote coupling, which likely improves the overall efficiency of the Nozaki–Hiyama–Kishi reaction [226–227] compared to the Fe/Cr or Co/Cr-mediated variants described in Kishi’s initial report. Of note, a subsequent TBS ether cleavage was necessary to remove the minor diastereomer from the allylation step. Reprotection of the diol **163** as the bis-TBS ether and ozonolysis of the homoallylic double bond provided the starting aldehyde for four (almost identical) HWE elongation cycles, leading to ethyl tetraenoate **164**. Base-mediated saponification then smoothly furnished acid **165** in 15 linear steps and 7.8% overall yield from aldehyde (*S*)-**17**.

In contrast to the pentaenoate series, the predominant product (>98%) of ethyl tetraenoate **164** was the all-*E* isomer, which was stable under the ester hydrolysis conditions. However, **164** could be equilibrated to a 4:3:3 mixture with its *Z*-Δ^4’,5’^ and the *Z*-Δ^6’,7’^-isomers by irradiation at 300 nm. Of note, the two minor geometric isomers of ester **164** were also prepared separately via a similar route using the Ando modification of the HWE reaction [228] to construct the *Z*-double bonds.

**Scheme 27:**
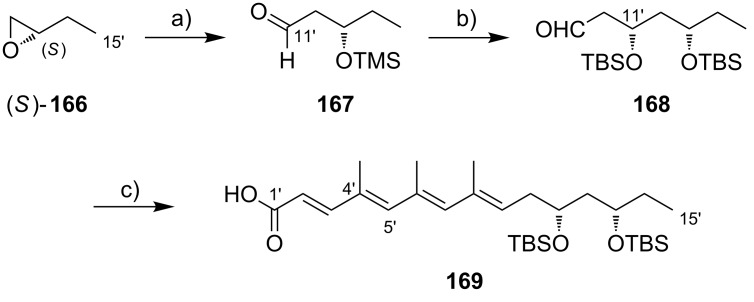
Kishi’s synthesis of the mycolactone E tetraenoate side chain. Reagents and conditions: a) (i) CH_2_=CHMgBr, CuI, Et_2_O, −30 °C to −20 °C; then TMSCl, DIPEA, −20 °C to 0 °C; (ii) OsO_4_, NMO, H_2_O, rt; (iii) Pb(OAc)_4_, benzene, rt, 67% (3 steps); b) (i) ligand L2*, CrBr_3_, Mn, Et_3_N, THF, 42 °C, then 2,6-lutidine, rt, then allyl bromide, aldehyde, Cp_2_ZrCl_2_, 0 °C; then 0.5 N HCl, rt, 55%, dr 95:5; (ii) TBSCl, imidazole, DMF, rt, 86%; (iii) OsO_4_, NMO, H_2_O, rt; (iv) Pb(OAc)_4_, benzene, rt, 93% (2 steps); c) (i) (EtO)_2_P(O)CH(Me)COOEt, *n*-BuLi, LiBr, THF, 0 °C, 95%; (ii) DIBAL-H, CH_2_Cl_2_, −78 °C, 89%; (iii) MnO_2_, CH_2_Cl_2_, rt, 94%; (iv)–(ix) 2× repeat steps i–iii, 40% (6 steps); (x) (EtO)_2_P(O)CHCOOEt, *n*-BuLi, THF, 0 °C to rt, 87%; (xi) LiOH, THF/MeOH/H_2_O 4:1:1, rt, 96%.

**III.2.2.3. Synthesis of the tetraenoate side chain of mycolactone E and its minor metabolite:** When Kishi and co-workers initiated their work on mycolactone E (**7**), two possible gross structures differing in the constitution of the polyunsaturated side chain (cf. Figure 2) had been proposed, again by the groups of Small [50] and Leadlay [56]. After re-examination of the available analytical data the Kishi group favored Leadlay’s structure, which only differed from the gross structure of mycolactone F (**8**) by the replacement of a methyl by an ethyl group at the terminal position of the polyunsaturated side chain [57]. Consequently, a similar synthesis strategy was chosen as for the mycolactone F fatty acid side chain. Although a *syn*-relationship of the 1,3-diol moiety was assumed, the absolute configuration was again unknown. Therefore, the Kishi group prepared both enantiomers of this tetraenoate. The synthesis was launched by a copper(I)-promoted regioselective opening of either enantiomer of 1,2-butylene oxide (**166**) with vinylmagnesium bromide**,** thus defining the stereochemistry at the C13’ position (exemplified in Scheme 27 by the synthesis of **169**).

Subsequent TMS protection of the resulting alcohol and oxidative cleavage of the double bond afforded aldehyde **167**which was subjected to the Cr/Zr/Mn-mediated Nozaki–Hiyama–Kishi coupling reaction, in analogy to the synthesis of the mycolactone F side chain (using a slightly modified version of ligand **L2** (not shown here)). After TMS cleavage and global TBS protection of the resulting diol, oxidative double bond cleavage delivered aldehyde **168**. The latter was then elaborated into tetraenoate **169** by the same sequence of transformations as in the synthesis of mycolactone F. **169** was obtained as the all-*E*-isomer in 18 linear steps and 7.9% overall yield starting from (*S*)-1,2-butylene oxide ((*S*)-**166**).

**Scheme 28:**
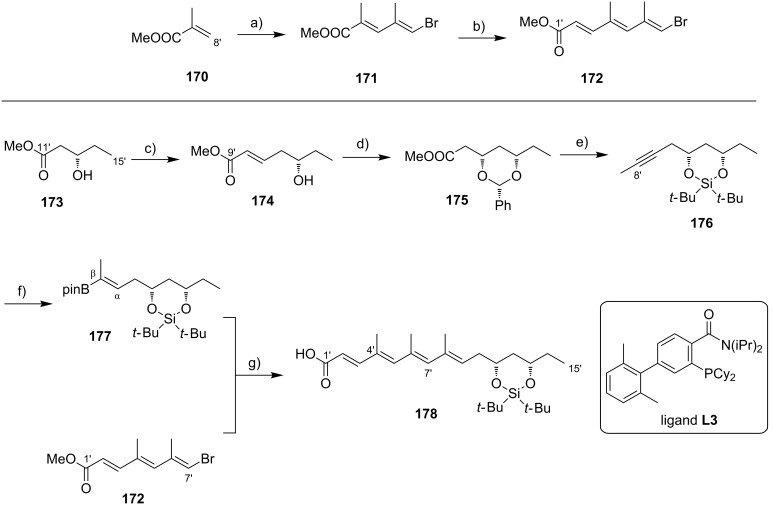
Wang and Dai’s synthesis of the mycolactone E tetraenoate side chain. Reagents and conditions: a) (i) Br_2_, CCl_4_, rt, then DBU, CCl_4_, rt; (ii) LiAlH_4_, CH_2_Cl_2_, 0 °C; (iii) MnO_2_, CH_2_Cl_2_, rt; (iv) Ph_3_P=C(Me)COOEt, CH_2_Cl_2_, 0 °C to rt, 63% (4 steps); b) (i) DIBAL-H, CH_2_Cl_2_, 0 °C; (ii) MnO_2_, CH_2_Cl_2_, rt; (iii) (MeO)_2_P(O)CH_2_COOMe, NaH, THF, −78 °C, 65% (3 steps); c) (i) TBSCl, imidazole, CH_2_Cl_2_, rt, 98%; (ii) DIBAL-H, CH_2_Cl_2_, 0 °C, 85%; (iii) DMP, CH_2_Cl_2_, NaHCO_3_, rt, 86%; (iv) (MeO)P(O)CH_2_COOMe, NaH, THF, −78 °C, 85%, 9:1 *E*/*Z*; (v) PPTS, MeOH, 50 °C, 85%; d) PhCHO, *t*-BuOK, THF, 0 °C, 65%; e) (i) H_2_, Pd(OH)_2_, MeOH, rt, 90%; (ii) *t*-Bu_2_Si(OTf)_2_, 2,6-lutidine, DMF, rt, 85%; (iii) DIBAL-H, CH_2_Cl_2_, −78 °C, 92%; (iv) CBr_4_, PPh_3_, Et_3_N, CH_2_Cl_2_, 93%; (v) *n*-BuLi, −78 °C, THF, then MeI, rt, 90%; f) B_2_(pin)_2_, CuCl (10 mol %), PCy_3_, *t*-BuONa, MeOH, toluene, rt, 75%, α:β 92.5:7.5; g) (i) Pd(OAc)_2_ (5 mol %), ligand **L3**, K_3_PO_4_, THF/H_2_O, 35 °C, 85%; (ii) LiOH, THF/MeOH/H_2_O 4:1:1, rt, 90%.

An alternative route to prepare the mycolactone E tetraenoate side chain acid was recently reported by Wang and Dai [229]. They aimed to provide a more convergent strategy that would combine a western triene fragment with an alkene bearing the chiral 1,3-diol moiety via Suzuki–Miyaura cross-coupling [230–231] thereby demonstrating the utility of their Aphos-Pd(OAc)_2_ catalyst system [232]. The synthesis of the trienyl bromide fragment **172** started from methyl methacrylate (**170**) that was transformed into the corresponding *E*-vinyl bromide via a bromination/elimination sequence (Scheme 28). LiAlH_4_ reduction to the alcohol, allylic oxidation with MnO_2_ and Wittig olefination of the ensuing aldehyde afforded dienyl bromide **171**. Another reduction/oxidation sequence followed by HWE olefination with trimethyl phosphonoacetate furnished trienyl bromide **172** in seven steps and 41% yield from methyl methacrylate. Only a single intermediate in this sequence required purification. The eastern fragment was accessed from methyl (*S*)*-*3-hydroxyvalerate ((*S*)-**173**), which was homologated to **174** in a four-step sequence involving a HWE olefination. The second hydroxy group was diastereoselectively introduced by intramolecular conjugate addition of the hemiacetal-derived alkoxide formed from **174** and benzaldehyde in the presence of potassium *tert-*butoxide [233]. After replacing the benzaldehyde acetal in **175** by a cyclic di*-tert*-butylsilyl ether, the selective reduction of the methyl ester to the corresponding aldehyde followed by a Corey–Fuchs alkynylation/methylation sequence furnished alkyne **176**. The stereo- and regioselective transformation of **176** into trisubstituted alkenyl boronate **177** was accomplished using a [Cu(I)PCy_3_]-catalyzed borylation [234]. The key Suzuki–Miyaura cross-coupling reaction was performed in 85% yield using the Aphos-Y ligand (**L3**) [232] under conditions that had been carefully optimized with a model substrate. The resulting methyl tetraenoate could be readily hydrolyzed to acid **178**. The latter was obtained in a longest linear sequence of 13 steps in 11% overall yield from (*S*)*-***173**.

**Scheme 29:**
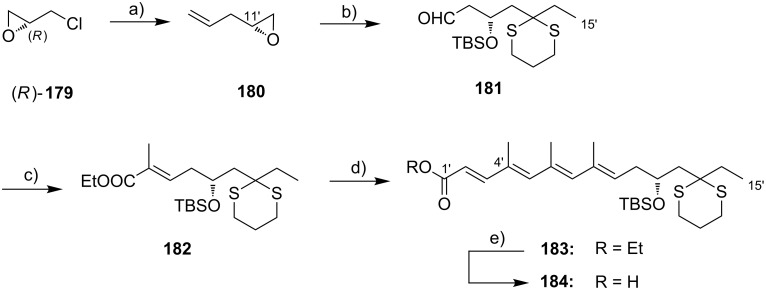
Kishi’s synthesis of the dithiane-protected tetraenoate side chain of the minor oxo-metabolite of mycolactone E. Reagents and conditions: a) (i) CH_2_=CHMgBr, cat. CuI, Et_2_O, −78 °C to −40 °C, 92%, ee >95% as determined by Mosher ester analysis [236]; (ii) KOH, distillation, 91%; b) (i) *t*-BuLi, 2-TBS-1,3-dithiane, Et_2_O, then EtI, HMPA, −78 °C to −25 °C, 64%; (ii) OsO_4_, K_3_Fe(CN)_6_, DABCO, MeSO_2_NH_2_, *t*-BuOH/H_2_O, 0 °C; (iii) Pb(OAc)_4_, benzene, 0 °C, 57% (two steps); c) (i) (EtO)_2_P(O)CH(Me)COOEt, *n*-BuLi, LiBr, MeCN, 0 °C to rt, 94%, *E*/*Z* 94:6; d) three HWE elongation cycles: (i) DIBAL-H, CH_2_Cl_2_, −78 °C; (ii) MnO_2_, CH_2_Cl_2_, rt; (iii) (EtO)_2_P(O)CH(Me)COOEt or (EtO)_2_P(O)CH_2_COOEt, *n*-BuLi, THF, 0 °C to rt, 72–95% over three steps, *E*/*Z* between 95:5 and 98:2; e) LiOH, THF/MeOH/H_2_O, 4:1:1 rt, quant.

When Kishi and co-workers set out to synthesize the minor oxo-metabolite of mycolactone E, its structure had not been unambiguously assigned [58] and the structural proposal [56] still needed to be confirmed by other means. Kishi and co-workers developed a synthesis relying on multicomponent anion relay chemistry [235] and iterative Horner–Wadsworth–Emmons elongation cycles. Starting from (*R*)-epichlorohydrin ((*R*)-**179**), epoxide opening with vinylmagnesium bromide in the presence of catalytic amounts of copper iodide followed by base-promoted intramolecular nucleophilic substitution of the ensuing chlorohydrin furnished epoxide **180** (Scheme 29). One-pot tandem alkylation of 2-TBS-1,3-dithiane with epoxide **180** and ethyl iodide exploiting an anion-relay mechanism, followed by oxidative cleavage of the terminal double bond gave aldehyde **181**, which was elaborated into α,β-unsaturated ester **182** by HWE chemistry.

Three subsequent reduction/oxidation/HWE-elongation cycles yielded ethyl tetraenoate **183** which was saponified to obtain acid **184**. The latter, which corresponds to the 1,3-dithiane and TBS-protected lower side chain of the mycolactone E minor metabolite, was obtained in 16 linear steps and 18% overall yield from (*R*)-epichlorohydrin ((*R*)-**179**).

**Scheme 30:**
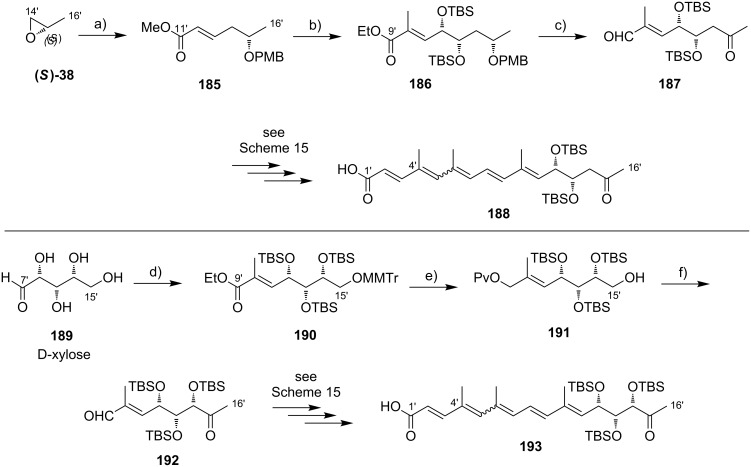
Kishi’s synthesis of the mycolactone S1 and S2 pentaenoate side chains. Reagents and conditions: a) (i) CH_2_=CHMgBr, CuI (10 mol %), Et_2_O, −30 °C to −20 °C; (ii) PMBBr, NaH, THF, DMF, 0 °C to rt, 87% (2 steps); (iii) OsO_4_ (1 mol %), NaIO_4_, 2,6-lutidine, dioxane, H_2_O, rt; (iv) Ph_3_P=CHCOOMe, toluene, 80 °C, 70% (2 steps); b) (i) AD mix-α, MeSO_2_NH_2_, dioxane, H_2_O, 0 °C, dr 8:1; (ii) TBSCl, AgNO_3_, pyridine, DMF, rt, 87% (2 steps); c) (i) DIBAL-H, CH_2_Cl_2_, −78 °C, 80% (major diastereomer); (ii) SO_3_∙pyridine, DIPEA, DMSO, CH_2_Cl_2_, rt, 95%; (iii) Ph_3_P=C(Me)COOEt, toluene, 90 °C; (iv) DIBAL-H, CH_2_Cl_2_, −78 °C, 78% (2 steps); (v) DDQ, CH_2_Cl_2_, H_2_O, 0 °C, 94%; (vi) DMP, NaHCO_3_, CH_2_Cl_2_, rt, 90%; d) (i) EtO_2_CCH(Br)Me, Zn, *n*-Bu_3_P, 1,4-dioxane, 100 °C, 56%, 3:1 *E*/*Z*; (ii) MMTrCl, pyridine, 0 °C, 70% (*E*-isomer); (iii) TBSCl, AgNO_3_, pyridine, DMF, rt, quant.; e) (i) DIBAL-H, CH_2_Cl_2_, −78 °C; (ii) PvCl, pyridine, DMAP, CH_2_Cl_2_, 0 °C, 98% (2 steps); (iii) HCO_2_H, Et_2_O, rt, 88%; f) (i) SO_3_∙pyridine, DIPEA, DMSO, CH_2_Cl_2_, rt, 95%; (ii) MeMgCl, Et_2_O, THF, 0 °C to rt, 93%; (iii) DMP, NaHCO_3_, CH_2_Cl_2_, rt, 84%.

**III.2.2.4. Synthesis of the pentaenoate side chains of mycolactones S1 and S2:** The synthesis of mycolactones S1 (**4**) and S2 (**5**) was reported by the Kishi group in 2012 [62]. After identification of those congeners from *M. ulcerans* subsp. *shinshuense* extracts by using their fluorogenic TLC method (vide supra), structural hypotheses were generated on the basis of (HR) MS/MS profiles. While mycolactone S1 (**4**) was assumed to be the C15’ keto analog of mycolactone A/B (**1a**,**b**), mycolactone S2 (**5**) was speculated to possess the C15’ keto group along with an additional hydroxy group at the C14’ position. In analogy to Kishi’s approach to the mycolactone E fatty acid side chain, the synthesis of the putative mycolactone S1 pentaenoate chain departed from (*S*)-propylene oxide ((*S*)*-***38**)**,** which was opened by copper(I)-mediated addition of vinylmagnesium bromide (Scheme 30). α,β-Unsaturated ester **185** was then obtained by PMB protection of the newly formed hydroxy group and subsequent Lemieux–Johnson oxidation [148] of the homoallylic double bond followed by a HWE olefination. Stereoselective introduction of the vicinal *syn*-diol by Sharpless dihydroxylation followed by TBS protection gave ester **186**. A subsequent reduction/oxidation/Wittig elongation cycle furnished the corresponding α,β-unsaturated ester. After PMB removal, several redox manipulations finally provided keto-aldehyde **187** in 12 steps and 28% overall yield from commercially available (*S*)*-***38**. Compound **187** was elaborated into the full length side chain acid **188** using the same strategy as in the synthesis of the mycolactone A/B side chain (see Scheme 15). In the case of mycolactone S2, both C14’-epimers were prepared since the local stereochemistry could not be deduced from the preliminary mass spectrometric analysis. The C14’ α-epimer, which is exemplified in Scheme 30, was accessed from D-xylose (**189**), which already incorporates the correctly configured C12’–C14’ stereotriad. Two-carbon elongation by zinc-mediated coupling with ethyl bromopropionate [237] followed by 4-methoxytrityl protection (MMTr) of the primary hydroxy group and subsequent TBS protection of the remaining alcohol groups furnished the α,β-unsaturated ester **190**. Reduction to the primary alcohol followed by protection with pivaloyl chloride and acid-mediated cleavage of the MMTr ether furnished **191**, bearing a free primary hydroxy group at the C15’ position. Swern oxidation to the corresponding aldehyde was succeeded by the addition of methylmagnesium chloride, with concurrent cleavage of the pivaloyl ester. The resulting diastereomeric mixture of diols was oxidized to key keto-aldehyde **192** again being processed according to the procedure presented in Scheme 15 to deliver pentaenoate **193** in 11 steps and 12% overall yield from **189**. The C14’ β-epimer was prepared from L-arabinose in similar yields using the same strategy.

#### III.3. Total synthesis of natural mycolactones

So far, total syntheses have been successfully completed for mycolactones A/B, C, E, (*dia*)-F, S1 and S2. All of the syntheses feature the same general endgame, including Yamaguchi-type esterification of the C5-hydroxy group with the respective, protected polyunsaturated side chain acid followed by protecting group removal. If a global TBS-protection strategy was employed, deprotection with TBAF as the fluoride source was performed in a single step. If the hydroxy groups at the core extension (upper side chain) were protected as a cyclopentylidene ketal (cf. structure **28**), initial removal of the side chain TBS groups with TBAF was followed by ketal cleavage under mildly acidic conditions to complete the synthesis.

**Scheme 31:**
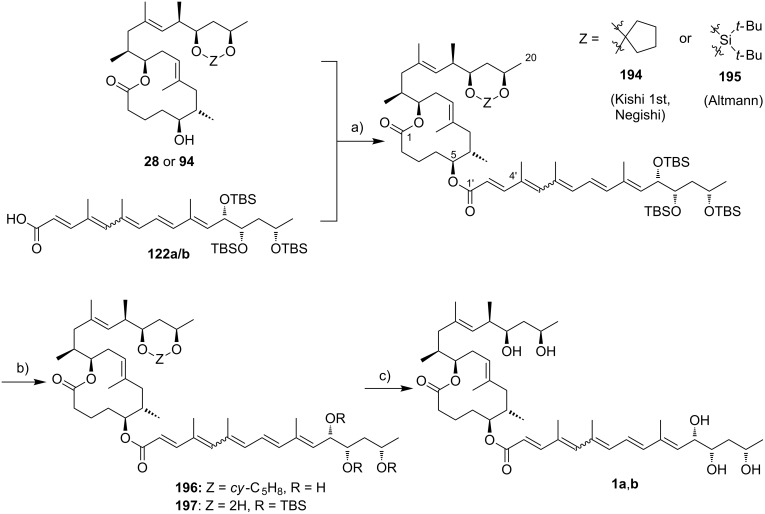
Kishi’s 1st generation and Altmann’s total synthesis of mycolactone A/B (**1a**,**b**) and Negishi’s selective synthesis of protected mycolactone A and B and their isomerization upon deprotection. Reagents and conditions: Kishi 1st generation approach*:* a) 2,4,6-trichlorobenzoyl chloride, DIPEA, DMAP, benzene, rt, 90%; b) TBAF, THF, rt, 81%; c) THF/HOAc/H_2_O, 2:2:1, rt, and the recovered starting material was recycled (once), 67% (*E*/*Z-*Δ^4’,5’^ 2:3). Negishi’s synthesis starting from *Z*-Δ^4’,5’^-**122**: a) 2,4,6-trichlorobenzoyl chloride, DIPEA, DMAP, benzene, rt, 6 h, 67%, >98% isomeric purity; b) TBAF, THF, rt, 71%, (*E*/*Z-*Δ^4’,5’^ ca. 1:4); c) THF/HOAc/H_2_O, 2:2:1, rt, 11 h and the recovered starting material was recycled (once), 59% (*E*/*Z-*Δ^4’,5’^ ca. 3:4). Negishi’s synthesis starting from *E-*Δ^4’,5’^*-***122***:* a) 2,4,6-trichlorobenzoyl chloride, DIPEA, DMAP, benzene, rt, 73%, >98% isomeric purity; b) TBAF, THF, rt, 70% (*E*/*Z-*Δ^4’,5’^ ca. 2:5); c) THF/HOAc/H_2_O, 2:2:1, rt, and the recovered starting material was recycled (once), 64% (*E*/*Z-*Δ^4’,5’^ ca. 4:5). Altmann’s approach*:* a) 2,4,6-trichlorobenzoyl chloride, DIPEA, DMAP, THF, rt, 89%; b) HF·pyridine, THF/pyridine 4:1, rt, 84%; c) TBAF, THF, rt, 85% (*E*/*Z-*Δ^4’,5’^ ca. 1:1, 10% minor isomers).

Specifically, Kishi’s 1st generation approach towards mycolactone A/B (**1a,b**) [43] relied on TBS protection of the lower side chain hydroxy groups, while the 1,3-diol at the core extension was protected as a cyclopentylidene ketal (Scheme 31). The same protecting group strategy was also part of Negishi’s projected individual syntheses of mycolactone A (**1a**) and mycolactone B (**1b**) [37], while in the mycolactone total syntheses from our own laboratory used a cyclic bis-*tert-*butylsilyl ether was used to mask the 1,3-diol motif at the core extension. In all cases, Yamaguchi esterification of the partially protected extended core structure proceeded smoothly to give the fully protected mycolactones **194** or **195**. While the polyunsaturated side chain acid in Kishi's case was a 2:3 *E*/*Z* mixture at the C4’–C5’ double bond, Negishi employed the pure *E-* and *Z-*Δ^4’,5’^ isomers, which had been obtained in separate syntheses (vide supra). The isomeric state of the side chain was maintained during the esterification reaction and in all three cases TBS deprotection with TBAF typically proceeded in good yield. However, despite the exclusion of light, partial isomerization of the C4’–C5’ double bond took place under these conditions, leading to isomeric product mixtures, even for the isomerically homogenous protected versions. Final removal of the cyclopentylidene ketal with acetic acid generally afforded the free mycolactones **1a**,**b** in moderate yield and further isomerization of the C4’–C5’ double bond was observed by Negishi. In summary, Kishi’s 1st generation approach employed a total of 20 steps for the longest linear sequence (from known **20**) and gave mycolactone A/B (**1a,b**) in 0.63% yield. Negishi’s total synthesis departed from (*R*)-methyl 3-hydroxybutyrate ((*R*)-**47**) and comprises 26 steps for the longest linear sequence. The mycolactones A/B were obtained and 2.8% overall yield, when using the *E-*Δ^4’,5’^*-*isomer of the lower side chain acid in the esterification step.

For our own total synthesis of mycolactone A/B (**1a,b**), the final deprotection involved first the cleavage of the bis-*tert-*butylsilyl ether in **195** with pyridine-buffered HF∙pyridine followed by TBAF-mediated removal of the TBS protecting groups from the lower side chain (Scheme 31) [178]. This two-step sequence furnished mycolactone A/B as a 1:1 mixture of the *E-*Δ^4’,5’^ and the *Z-*Δ^4’,5’^ isomer containing ca. 10% of minor isomers. The two-step procedure was required since extended treatment of **195** with buffered HF·pyridine, as it was required for TBS cleavage, caused partial decomposition, while TBAF alone did not efficiently remove the cyclic silyl-ether protecting group. More recently, however, we have found that the sequential addition of TBAF followed by an excess of ammonium fluoride allowed for efficient, one-pot global deprotection [111]. Overall, our synthesis comprises a longest linear sequence of 19 steps and produced the target structure in 13% overall yield according to [178]. The synthesis, thus, is significantly more efficient than either Kishi's 1st generation approach or the Negishi synthesis although recent unpublished optimizations (vide supra) were not considered.

**Scheme 32:**
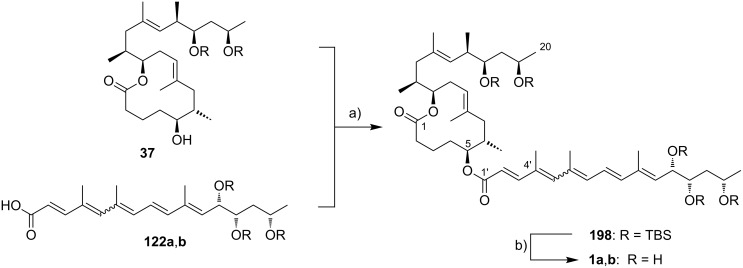
Kishi’s 2nd generation total synthesis of mycolactone A/B (**1a**,**b**). Reagents and conditions: a) 2,4,6-trichlorobenzoyl chloride, DIPEA, DMAP, benzene, rt, 90%; b) TBAF, THF, rt, 80% (*E*/*Z-*Δ^4’,5’^ 2:3).

In contrast to his first generation synthesis, Kishi’s 2nd generation approach to mycolactone A/B (**1a,b**) incorporated a global TBS-protection strategy that was also maintained in his 3rd generation approach towards the mycolactone core and in all syntheses of other mycolactone congeners (Scheme 32). Global TBS deprotection of **198** with TBAF furnished the typical mixture of mycolactones A and B.

Interestingly, and contrary to the observations by Negishi and co-workers, the Kishi group found *E*/*Z* isomerization of the C4’–C5’ double bond under their TBAF deprotection conditions to be less pronounced. Thus, deprotection of a chromatographically enriched mixture of **198** predominantly containing the *Z-*Δ^4’,5’^-isomer (10:1) yielded a 6:1 mixture of **1a** and **1b**, if light was carefully excluded. With a longest linear sequence of 21 steps and an overall yield of 8.9%, Kishi’s 2nd generation synthesis of mycolactone A/B represented a significant advance over his 1st generation approach.

Kishi’s syntheses of other natural mycolactones uniformly relied on the 2nd generation strategy developed for the synthesis of mycolactones A/B (**1a,b**) and will therefore not be discussed here in detail. Briefly, mycolactone C (**2**) was prepared in two steps from the partially TBS-protected mycolactone core **37** and the TBS-protected mycolactone C side chain **154** as an equimolar mixture of *Z-*Δ^4’,5’^ and *E-*Δ^4’,5’^-isomers in 76% yield [53]. The same two-step procedure gave mycolactones E (**6**) and *dia*-F (*dia*-**8**) in 62% and 56% yield, respectively [57,59]. Both, mycolactone E and mycolactone *dia*-F, were obtained as 100:4:4 mixtures of the all-*E*, the *Z-*Δ^4’,5’^ and the *Z-*Δ^6’,7’^ isomers, respectively. The minor metabolite of mycolactone E was prepared in a similar manner using side chain acid **184**; however, dithiane deprotection mediated by *N*-chlorosuccinimide and silver nitrate had to be performed prior to global TBAF-promoted silyl ether cleavage. The latter was relatively inefficient (44% yield) and product **7** was finally obtained in 3 steps and 35% yield [58]. Another minor modification of the strategy had to be made to prepare the two oxidized congeners mycolactones S1 (**4**) and S2 (**5**) [62]. While Yamaguchi esterification with the respective side chain acids uneventfully provided the protected mycolactones, TBAF-mediated deprotection resulted in a complex mixture of products. Ultimately, buffering the TBAF solution with imidazole hydrochloride cleanly furnished the desired products, although extended reaction times (5 d) were necessary. Mycolactones S1, S2-14’α and S2-14’β were obtained as the typical Δ^4’,5’^*E*/*Z* mixtures in 58%, 74% and 94% yield, respectively (over two steps). Finally, natural mycolactone S2 was proven to be equivalent to S2-14’α.

Our own approach towards mycolactone C (**2**) also relied on Yamaguchi esterification of the mycolactone core with the mycolactone C fatty acid side chain, both being protected as cyclic bis-*tert-*butylsilyl ethers. In contrast to the lower mycolactone A/B side chain, the mycolactone C pentaenoate chain was tolerant to pyridine-buffered HF∙pyridine, thus enabling smooth global deprotection. Mycolactone C (**2**) was obtained in 59% yield over two steps as a 66:27:7 mixture of isomers (*E-*Δ^4’,5’^/*Z-*Δ^6’,7’^/minor).

### IV. Synthesis of mycolactone analogs

#### IV.1. Modifications of the extended mycolactone core

**Scheme 33:**
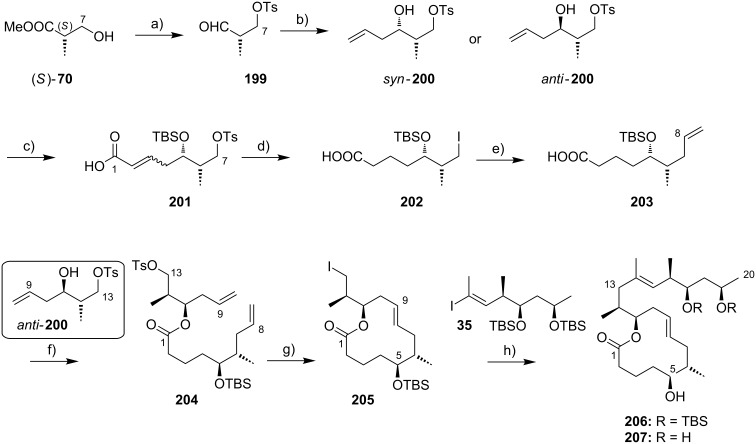
Blanchard’s synthesis of the 8-desmethylmycolactone core. Reagents and conditions: a) (i) TsCl, TEA, DMAP, CH**_2_**Cl_2,_ 0 °C to rt; (ii) DIBAL-H, toluene, −78 °C to rt, 87% (2 steps); (iii) TEMPO (10 mol %), PhI(OAc)_2_, CH_2_Cl_2,_ 10 °C to rt; b) (−)- or (+)-Ipc_2_Ballyl, Et_2_O, −78 °C, then NaBO_3_·4H_2_O, 70%, dr *>* 97:3 for *syn*-**200** and 76%, dr *>* 97:3 for *anti*-**200**; c) (i) TBSCl, imidazole, CH_2_Cl_2_, rt, 95%; (ii) acrylic acid, Grubbs II (3 mol %), CH_2_Cl_2_, 90 °C (µw); d) (i) H_2_, Pd(OH)_2_, EtOAc, 79% (2 steps); (ii) NaI, acetone, reflux, 90%; e) (i) vinylmagnesium bromide, FeCl_3_ (20 mol %), TMEDA, THF, 0 °C, 51%; f) *anti*-**200**, DCC, DMAP, CH_2_Cl_2_, 0 °C to rt, 82%; g) (i) Grubbs II (10 mol %), CH_2_Cl_2_, 90 °C (µw), 83%; (ii) NaI, acetone, reflux, 92%; h) (i) Li, naphthalene, ZnCl_2_, THF, rt, then **205**, benzene/DMF 15:1, rt, then **35**, Pd(PPh_3_)_4_ (13 mol %), LiCl, NMP, 55 °C, 63%; (ii) HF∙pyridine, pyridine, THF, 0 °C, 4 h (yields **206**) or 15 h (yields **207**), **206**: 42% or **207**: 81%.

In 2011, Blanchard and co-workers reported a synthesis of the extended C8-desmethylmycolactone core, which is strategically related to our own approach towards the analogous “natural” fragment (Scheme 33, cf. Scheme 7 and Scheme 8). The key steps in Blanchard's synthesis of the 8-desmethylmycolactone core include the closure of the macrolactone ring by RCM and the attachment of the C14−C20 core extension to the C13 atom via C(sp^2^)–C(sp^3^) cross-coupling. The synthesis started from (*S*)*-*Roche ester (*S*)-**70**, which was tosylated and converted into aldehyde **199**. The latter served as the substrate for a subsequent asymmetric Brown allylation that was performed either with (−)- or (+)-Ipc_2_B(allyl)borane to furnish *syn-* and *anti-***200**, respectively. The *syn*-diastereomer was TBS protected and subjected to cross metathesis with acrylic acid in methylene chloride under microwave heating using Grubbs 2nd generation catalyst. The double bond of the resulting acrylate **201** was reduced by hydrogenation in the presence of Pearlman’s catalyst and the tosylate was converted to the corresponding iodide under Finkelstein conditions. By applying Cossy’s iron-mediated C(sp^2^)–C(sp^3^) cross-coupling methodology [238], alkyl iodide **202** was fused with vinylmagnesium bromide to produce alkene **203**. Interestingly, no protection of the carboxylic acid moiety was required in the presence of an excess of vinylmagesium bromide. Acid **203** was activated with DCC and esterified with *anti*-**200** under Steglich conditions to produce diene **204**. This diene readily underwent RCM-mediated cyclization with Grubbs 2nd generation catalyst again in overheated methylene chloride. After cyclization, the C13 tosylate was transformed into the corresponding alkyl iodide **205** under Finkelstein conditions, which was then connected with known vinyl iodide **35** by Negishi cross-coupling.

Treatment of the coupling product with HF∙pyridine only led to cleavage of the C5-TBS ether (producing **206**), while global silyl ether cleavage to **207** occurred only after extended reaction times. The extended C8-desmethylmycolactone core was prepared in 14 steps (longest linear sequence) and 6.7% overall yield from (*S*)*-*Roche ester ((*S*)-**70**).

**Scheme 34:**
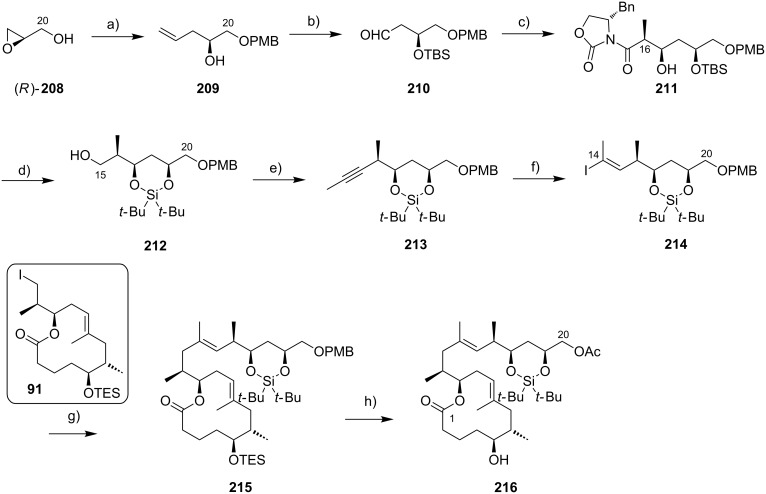
Altmann’s (partially unpublished) synthesis of the C20-hydroxylated mycolactone core. Reagents and conditions: a) (i) NaH, PMBCl, cat. TBAI, DMF, rt, 79%; (ii) vinylmagnesium bromide, CuI, THF, −20 °C, 97%; b) (i) TBSCl, imidazole, CH_2_Cl_2_, reflux, 97%; (ii) K_2_OsO_4_∙2H_2_O, NMO, acetone/H_2_O 9:1, rt; (iii) NaIO_4_, THF/H_2_O 4:3, rt, 98% (2 steps); c) (*S*)-4-benzyl-3-propionyloxazolidin-2-one, Et_2_BOTf, DIPEA, CH_2_Cl_2_, 0 °C; then **210**, −78 °C to 0 °C, 77%, de 20:1; d) (i) HF·pyridine, THF, rt, 98%; (ii) *t*-Bu_2_Si(OTf)_2_, pyridine, CH_2_Cl_2_, rt, 98%; (iii) NaBH_4_, THF/water 4:1, rt, 91%; e) (i) DMP, NaHCO_3_, CH_2_Cl_2_, rt, 92%; (ii) CBr_4_, PPh_3_, 0 °C, 96%; (iii) *n*-BuLi, THF, −78 °C, then MeI, −78 °C to rt, 92%; f) Cp_2_Zr(H)Cl, THF, 40 °C, then I_2_, 0 °C, 90%; g) **91**, *t*-BuLi, 9-MeO-9-BBN, Et_2_O, THF, −78 °C to rt, then **214**, Pd(dppf)Cl_2_ (10 mol %), AsPh_3_, Cs_2_CO_3_, DMF, H_2_O, rt, 98%; h) (i) DDQ, CH_2_Cl_2_/H_2_O 5:1, rt, 99%; (ii) Ac_2_O, DIPEA, DMAP, CH_2_Cl_2_, rt, quant.; (iii) THF/H_2_O/HOAc 3:1:1, rt, quant.

A modified version of the extended mycolactone core with a hydroxy tag at the C20 position was designed in our own group [90,111]. The additional hydroxy group enables the attachment of various residues for SAR and target elucidation studies. The synthesis started from commercially available (*R*)-glycidol ((*R*)-**208**), thus immediately setting the configuration of the C19 stereocenter (Scheme 34). PMB protection of the primary hydroxy group followed by regioselective copper(I)-mediated epoxide opening with vinylmagnesium bromide furnished homoallylic alcohol **209** that was TBS protected and subjected to oxidative double bond cleavage under Upjohn/Lemieux–Johnson conditions. The ensuing aldehyde **210** was subjected to an asymmetric Evans aldol addition to simultaneously establish the stereochemistry at the C16 and C17 positions in **211**.

Replacement of the TBS protecting group by a cyclic di-*tert-*butylsilyl ether blocking the 1,3-diol followed by reductive removal of the Evans auxiliary with NaBH_4_ then gave primary alcohol **212** that was transformed into the corresponding aldehyde with Dess–Martin periodinane. Application of the two-step Corey–Fuchs protocol and trapping of the alkynyllithium intermediate with methyl iodide provided alkyne **213**. Hydrozirconation followed by a zirconium–iodine exchange then furnished key vinyl iodide **214** (Gersbach, Gehringer, Bucher & Altmann, unpublished). C(sp^2^)–C(sp^3^) Suzuki coupling with the mycolactone core (**91**) proceeded smoothly in almost quantitative yield under optimized conditions (Gehringer & Altmann, unpublished). A replacement of the C20 PMB protecting group was necessary to enable an orthogonal deprotection of the C20 hydroxy group in the presence of the mycolactone lower side chain at a later stage [111]. Therefore, the PMB ether was cleaved with DDQ followed by DMAP-promoted acetylation of the liberated hydroxy group with acetic anhydride. Finally, cleavage of the C5-TES ether furnished the adequately protected modified mycolactone core **216** in 17 steps and 35% overall yield from (*R*)-glycidol ((*R*)-**208**).

**Scheme 35:**
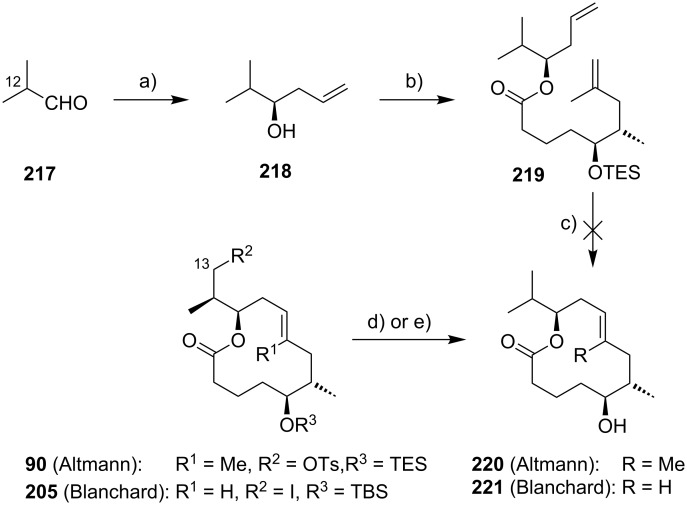
Altmann’s and Blanchard’s approaches towards the 11-isopropyl-8-desmethylmycolactone core. Reagents and conditions: **220**: a) AllylSnBu_3_, Ti(OiPr)_4_, (*S*)-(−)-1,1’-bi-2-naphthol, CH_2_Cl_2_, 4 Å molecular sieves, −78 °C to −18 °C, 47%, single isomer; b) **88**, EDCI, DMAP, CH_2_Cl_2_, rt, 71%; c) Grubbs II, Grubbs I or Hoveyda–Grubbs II catalyst; CH_2_Cl_2_ or toluene; d) (i) NaBH_4_, DMSO, 100 °C, 76%; (ii) HOAc/THF/H_2_O 2:2:1, rt, 98%. **221**: e) (i) Li, naphthalene, ZnCl_2_ THF, rt, then **205**, benzene/DMF 15:1, rt, then aq NH_4_Cl, 60%; (ii) HF∙pyridine, pyridine, 40 °C, 89%.

In order to assess the importance of the upper side chain for biological activity, we have also prepared simplified mycolactones lacking the C14–C20 part of the core extension [178]. In an initial attempt, we investigated the synthesis of the truncated extended core structure **220** by RCM of diene **219** (Scheme 35).

However, the latter proved to be resistant to ring closure under the conditions that had proven effective for the cyclization of **89** and any other of the conditions screened, including the use of different metathesis catalysts (Grubbs 1st generation [239], Grubbs 2nd generation [171], or Hoveyda–Grubbs 2nd generation [189]) and solvents (CH_2_Cl_2_ or toluene).

This observation is in line with results from the Burkart [173,240] and the Blanchard [182] groups, showing that the successful closure of the 12-membered ring is sensitive to subtle changes of the substituent at the C13 position. As an alternative to the RCM-based cyclization of **219**, lactone **220** could eventually be obtained in good yields by reduction of tosylate **90** with an excess of NaBH_4_ in DMSO at 100 °C followed by the removal of the TES protecting group under slightly acidic conditions. The synthesis of the 8-desmethyl analog of **220**, i.e., **221** has been reported by Blanchard and co-workers starting from iodide **205** [92]. An iodine–zinc exchange with Rieke zinc [241] followed by aqueous quenching smoothly reduced the C13 position and subsequent cleavage of the C5-TBS ether by pyridine-buffered HF∙pyridine uneventfully yielded the free alcohol **221**.

**Scheme 36:**
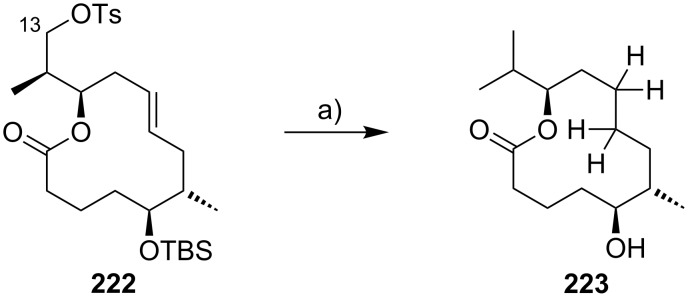
Blanchard’s synthesis of the saturated variant of the C11-isopropyl-8-desmethylmycolactone core. Reagents and conditions: a) (i) H_2_ (1 atm), Pd(OH)_2_, EtOAc, rt, 77%; (ii) NaI, acetone, reflux, 80%; (iii) Zn, LiCl, TMSCl, BrCH_2_CH_2_Br, THF, rt, then alkyl iodide, rt, then aq NH_4_Cl, 75%; (iv) HF∙pyridine, pyridine, THF, 0 °C to 40 °C, 59%.

Blanchard also prepared a saturated analog of **221** via tosylate **222** [242]. Hydrogenation of this intermediate over Pearlman’s catalyst, followed by Finkelstein iodination, metalation/protonation, and TBS-ether cleavage with buffered HF∙pyridine finally gave **223** in 27% overall yield from **222** (Scheme 36).

#### IV.2 Modifications of the lower mycolactone side chain

**Scheme 37:**
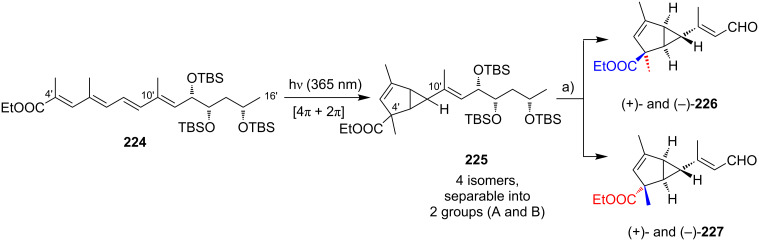
Structure elucidation of photo-mycolactones generated from tetraenoate **224**.

In a recent publication, the Kishi group reported the synthesis of non-natural mycolactones that they have termed photo-mycolactones [243]. This work was triggered by the fact that mycolactone A/B completely loses its activity against keratinocytes after 30 min of exposure to light [244]. Earlier findings of the Kishi group had already shown that synthetic mycolactone A/B upon light exposure was cleanly transformed into four closely related compounds that were denominated as photo-mycolactones A1, A2, B1 and B2 [244]. These compounds were isomeric to mycolactone A/B, but they could not be properly separated. Thus, the Kishi group started to study the photochemical behavior of the isolated, protected mycolactone A/B pentaenoate side chain and its tetraenoate analog. The photoproducts in the tetraenoate series (**225**) were separable into two groups (A and B) containing two compounds each (Scheme 37). Upon TBS deprotection, the two constituents of each group could be finally separated by HPLC on a chiral stationary phase. Oxidative diol cleavage of those products gave two matching pairs of levorotatory and dextrorotatory aldehydes ((+)-/(−)-**226** and (+)-/(−)-**227**, Scheme 37).

Strikingly, the synthesis of the tri-*p*-bromobenzoate variants of compound **225** furnished a crystalline product that was analyzed by X-ray crystallography. The crystallographic data showed the photoproducts to be bicyclo[3.1.0]cyclohexene derivatives, which was in line with the structural proposal that had already been derived by NMR spectroscopy. Based on further experiments and literature data, the Kishi group proposed a concerted [4π_s_ + 2π_a_] cycloaddition as the mechanism for the cyclization. In a seven-step sequence, they then converted the four photoproducts prepared from the tetraenoate series into their pentaenoate-derived analogs that were subsequently attached to the mycolactone core, to yield the complete photo-mycolactones. The synthetic photo-mycolactones were found to be identical with the compounds obtained by direct photocyclization of mycolactone A/B. Since all mycolactone side chains presented in the following section were attached to the mycolactone core and deprotected according to the same general methodologies as presented in the total synthesis section (i.e., Yamaguchi esterification with the C5-hydroxy group and subsequent silyl ether cleavage by different fluoride sources), these steps will not be discussed. The interested reader is referred to the cited literature.

**Scheme 38:**
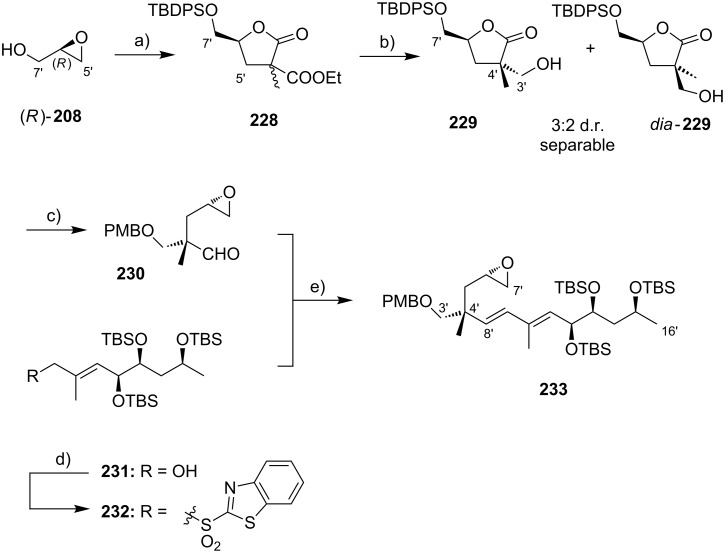
Kishi’s synthesis of the linear precursor of the photo-mycolactone B1 lower side chain. Reagents and conditions: a) (i) TBDPSCl, imidazole, CH_2_Cl_2_, rt; (ii) MeCH(CO_2_Et)_2_, LiHMDS, AlEt_3_, THF, −78 °C, then *p*-TsOH, 88%; b) (i) KOH, EtOH, H_2_O, 0 °C, 99%; (ii) ClCO_2_Et, TEA, THF, 0 °C, then NaBH_4_, iPrOH, rt, 38% (**229**) and 31% (*dia-***229**); c) (i) PMB-trichloroacetimidate, La(OTf)_3_, toluene, 0 °C to rt, 87%; (ii) LiBH_4_, MeOH, THF, 0 °C, 98%; (iii) TIPSCl, imidazole, DMF, rt, 93%; (iv) NaOH, MeOH, H_2_O, 60 °C, 69%; (v) TsCl, TEA, *n*-Bu_2_SnO, CH_2_Cl_2_, rt; (vi) K_2_CO_3_, MeOH, rt, 88% (2 steps); (vii) TBAF, THF, rt; (viii) TEMPO, NaClO_2_, KBr, NaHCO_3_, CH_2_Cl_2_, H_2_O, −10 °C, 86% (2 steps); d) (i) benzothiazole-2-thiol, PPh_3_, DIAD, THF, 0 °C to rt, 98%; (ii) H_2_O_2_, (NH_4_)_6_Mo_7_O_24_∙4H_2_O, EtOH, 0 °C to rt, 86%; e) LiHMDS, THF, −78 °C, 86%.

Following the structure elucidation of photo-mycolactones, the Kishi laboratory embarked on the total synthesis of these compounds [243]. The envisaged key step was an intramolecular LiTMP-promoted Hodgson cyclopropanation [245–246], that would transform a chiral 1,2-epoxy-5-ene into the desired bicyclo[3.1.0]cyclohexene skeleton in a stereocontrolled fashion. Starting from (*R*)-glycidol ((*R*)-**208**, exemplified in Scheme 38) or (*S*)-glycidol, TBDPS protection followed by base-promoted epoxide opening with diethyl methylmalonate and intramolecular transesterification furnished γ-lactone **228** as an inseparable mixture of diastereomers. After selective hydrolysis of the exocyclic ester group, an acid activation/reduction sequence furnished a 3:2 mixture of the diastereomeric primary alcohols **229** and *dia*-**229** that was separable by column chromatography. Similarly, (*S*)-**208** furnished the corresponding enantiomers *ent-***229** and *ent-dia-***229** and every single stereoisomer was processed separately. As an example, **229** was then elaborated into epoxyaldehyde **230** in an eight-step sequence that involved reductive opening of the lactone, selective tosylation of the primary hydroxy group and subsequent base-promoted epoxide formation as the key transformations. The aldehyde was converted into diene **233** by a one-pot Julia olefination [247–248] with **232**; the latter was easily prepared from known precursor **231** in a Mitsunobu reaction/*S*-oxidation sequence. Following the same route, all four stereoisomers varying in the configuration of C4’ and the C6’ were prepared.

**Scheme 39:**
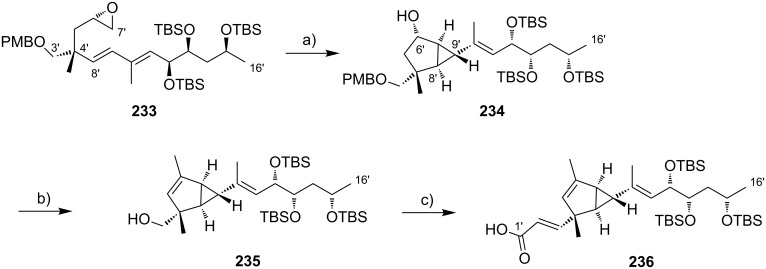
Kishi’s synthesis of the photo-mycolactone B1 lower side chain. Reagents and conditions: a) LiTMP, MTBE, −10 °C, 34%, single diastereomer; b) (i) TPAP, NMO, CH_2_Cl_2_, H_2_O, rt, 72%; (ii) LDA, 5-Cl-2-PyNTf_2_, THF, −78 °C; (iii) LiCuMe_2_, THF, −15 °C, 81% (2 steps); (iv) DDQ, CH_2_Cl_2_, *t*-BuOH, phosphate buffer pH 7.0, rt, 51%; c) (i) DMP, CH_2_Cl_2_, rt, 84%; (ii) Ph_3_P=CHCOOMe, toluene, 90 °C, 87%; (iii) NaOH, THF/MeOH/H_2_O 4:1:1, rt, 90%.

Epoxide **233** was then subjected to LiTMP-mediated Hodgson cyclopropanation, to deliver hydroxylated bicyclo[3.1.0]cyclohexane **234** with excellent stereoselectivity, but in relatively low yields; although most of the starting material could be recovered (Scheme 39). Of note, yields could be significantly increased (65% vs 34%) by replacing the PMB protecting group with a 2-methoxyethoxymethyl (MEM) group and changing the solvent to diethyl ether. The oxidation of **234** to the corresponding ketone under Ley–Griffith conditions [249] followed by enol triflate formation and subsequent coupling with lithium dimethylcopper (“Gilman reagent”) [250] introduced the C6’-methyl group. The cleavage of the PMB ether with DDQ then furnished primary alcohol **235** in 19 steps and 1.2% overall yield from (*R*)-glycidol ((*R*)-**208**). Alcohol **235** was processed to α,β-unsaturated acid **236** in a three-step oxidation/Wittig olefination/saponification sequence, according to Kishi’s first report on photo-mycolactones [244] (66% yield). The other three photo-mycolactone side chain acids were prepared in the same manner from *dia-***229**, *ent-***229** and *ent-dia-***229**.

**Scheme 40:**
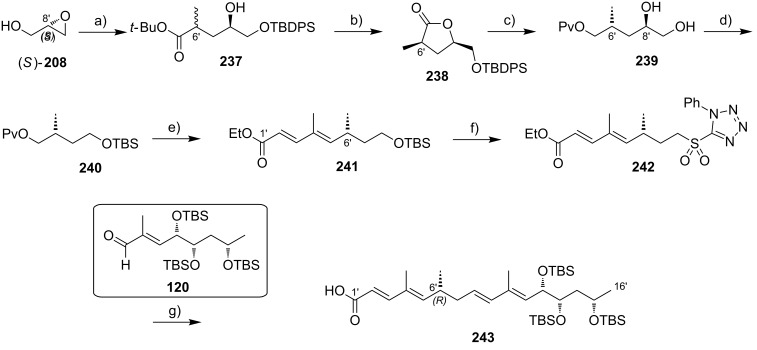
Kishi’s synthesis of a stabilized lower mycolactone side chain. Reagents and conditions: a) (i) TBDPSCl, imidazole, CH_2_Cl_2_, rt, 96%; (ii) MeCH_2_COO-*t*-Bu, LiHMDS, AlEt_3_, THF, −78 °C, 95%; b) (i) PTSA, CHCl_3_, reflux, 96%; (ii) LDA, THF, −78 °C, then 2,6-di-*tert*-butylphenol, −78 °C, dr 8–10:1, then recrystallization, 65%, dr 50–100:1; c) (i) LiBH_4_, THF, MeOH, 0 °C, 97%; (ii) PvCl, pyridine, CH_2_Cl_2_, 0 °C to rt, 90%; (iii) TBAF, THF, rt, 96%; d) (i) NaIO_4_, THF, H_2_O, 0 °C, 93%; (ii) NaBH_4_, MeOH, 0 °C, 96%; (iii) TBSCl, imidazole, CH_2_Cl_2_, rt, 94%; e) (i) DIBAL-H, CH_2_Cl_2_, −78 °C, 92%; (ii) SO_3_∙pyridine, DIPEA, DMSO, CH_2_Cl_2_, 0 °C to rt, 90%; (iii) Ph_3_P=C(Me)COOEt, CH_2_Cl_2_, rt, 90%; (iv) DIBAL-H, CH_2_Cl_2_, −78 °C, 94%; (v) MnO_2_, CH_2_Cl_2_, rt, 92%; (vi) (EtO)_2_P(O)CH_2_COOEt, *n*-BuLi, THF, 0 °C to rt, 93%; f) (i) PPTS, EtOH, rt, 90%; (ii) 1-phenyl-1*H*-tetrazole-5-thiol, DIAD, PPh_3_, THF, 0 °C, 94%; (iii) H_2_O_2_, (NH_4_)_6_Mo_7_O_24_∙4H_2_O, EtOH, 0 °C to rt, 90%; g) (i) KHMDS, THF, −78 °C, 90%; (ii) LiOH, THF/MeOH/H_2_O 4:1:1, rt, 92%. Using the same reaction sequence the C6’-(*S*) epimer of **242** was prepared from (*R*)*-*glycidol ((*R*)-**208**).

In their latest contribution to mycolactone chemistry, Kishi and co-workers reported mycolactone analogs with a partially saturated lower side chain [251]. Motivated by their studies on the photochemical behavior of mycolactones [243–244], they sought to stabilize the lower side chain by saturating the central double bond of the pentaene system. Since the saturation generates a stereocenter at C6’, a novel stereoselective synthesis strategy was required. This route is exemplified in Scheme 40 for the (*R*)-C6’ epimer **243**. Starting from (*S*)-glycidol ((*S*)-**208**), TBDPS protection and regioselective epoxide opening by the anion of *tert*-butyl propionate in the presence of AlEt_3_ furnished secondary alcohol **237** as an epimeric mixture at the C6’ position. Acid treatment then induced the formation of the corresponding five-membered lactone, which was deprotonated with LDA and re-protonated under kinetic control with 2,6-di-*tert*-butylphenol to provide lactone **238** in high diastereomeric purity after recrystallization.

Reductive lactone opening and selective protection of the ensuing primary hydroxy group as the pivalate followed by TBDPS cleavage afforded vicinal diol **239**, which was subjected to periodate-mediated diol cleavage. Reduction of the resulting aldehyde and protection with TBS chloride yielded orthogonally protected diol **240**, with the C6’-stereocenter in place. The pivaloyl group was removed reductively and two subsequent Wittig-elongation cycles gave the C1’–C8’ fragment **241**. Conversion into the corresponding 1-phenyl-1*H*-tetrazol-5-yl sulfone **242** was achieved in a three-step deprotection/Mitsunobu/oxidation sequence and **242** was then reacted with aldehyde **120** under Julia–Kocienski conditions. The ensuing full length C1’–C16’ fragment was saponified with lithium hydroxide to finally yield acid **243** in 21 linear steps and 15% overall yield. The C6’-(*S*)-epimer was prepared via the same route.

**Scheme 41:**
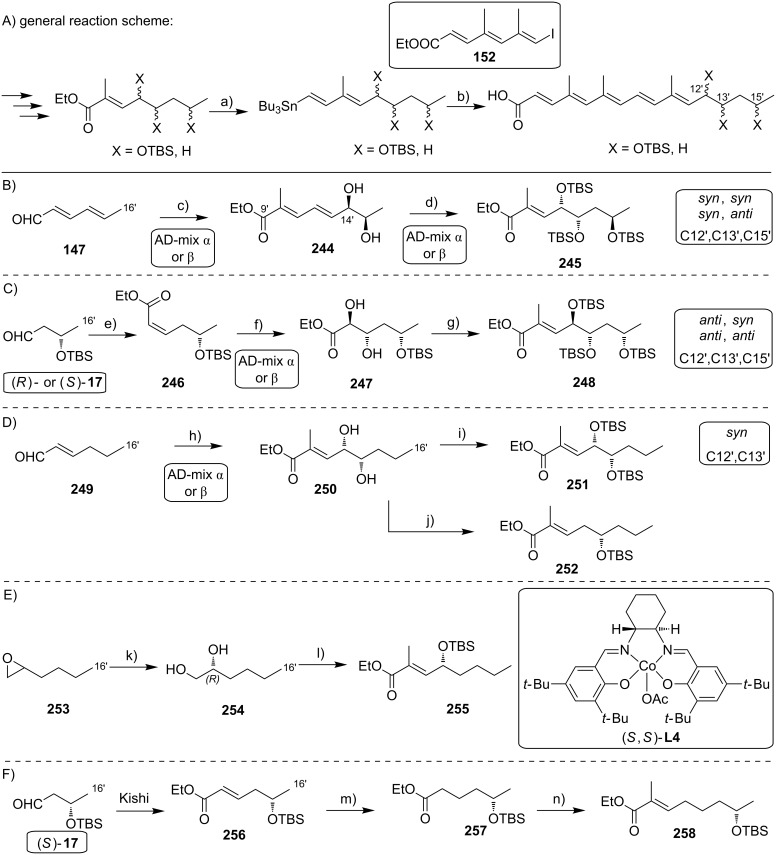
Blanchard’s variation of the C12’,C13’,C15’ stereocluster. Reagents and conditions: a) (i) DIBAL-H, CH_2_Cl_2_, 0 °C; (ii) MnO_2_, CH_2_Cl_2_, reflux; (iii) CrCl_2_, CHI_3_, THF, rt; (iv) *n*-BuLi, Bu_3_SnCl, Et_2_O, −78 °C; b) (i) CuTC, Ph_2_P(O)ONBu_4_, NMP, rt; (ii) LiOH, THF, MeOH, H_2_O, rt; c) (i) Ph_3_P=C(Me)COOEt, CH_2_Cl_2_, rt, 99%; (ii) AD-mix α, K_2_OsO_4_·2H_2_O (0.6 mol %), MeSO_2_NH_2_, *t*-BuOH, H_2_O, 0 °C, 70%; d) (i) triphosgene, pyridine, CH_2_Cl_2_, rt, 63%; (ii) Pd_2_(dba)_3_·CHCl_3_ (0.5 mol %), HCO_2_H, Et_3_N, THF, rt, 80%; (iii) TBSCl, imidazole, DMAP, DMF, rt, 83%; (iv) AD-mix α, K_2_OsO_4_·2H_2_O (2 mol %), MeSO_2_NH_2_, *t*-BuOH, H_2_O, 0 °C, 73%; (v) TBSCl, imidazole, DMAP, DMF, rt, 82%; e) (*o*-Tol)_2_P(O)CH_2_COOEt, NaI, NaH, THF, −78 °C, 75%; f) AD-mix α, K_2_OsO_4_·2H_2_O (0.6 mol %), MeSO_2_NH_2_, *t*-BuOH, H_2_O, 0 °C, 62%, dr 9:1; g) (i) TBSCl, imidazole, DMAP, DMF, rt, 63%; (ii) DIBAL-H, CH_2_Cl_2_, 0 °C, 73%; (iii) TEMPO, PhI(OAc)_2_, CH_2_Cl_2_, rt; (iv) Ph_3_P=C(Me)COOEt, ClCH_2_CH_2_Cl, 70 °C, 65% (2 steps); h) (i) Ph_3_P=C(Me)COOEt, CH_2_Cl_2_, rt, 98%; (ii) AD-mix α, K_2_OsO_4_·2H_2_O (0.6 mol %), MeSO_2_NH_2_, *t*-BuOH, H_2_O, 0 °C, 95%, ee >99%; i) TBSCl, imidazole, DMAP, DMF, rt, 97%; j) (i) triphosgene, pyridine, CH_2_Cl_2_, rt, 76%; (ii) Pd_2_(dba)_3_·CHCl_3_ (0.5 mol %), HCO_2_H, Et_3_N, THF, rt, 76%; (iii) TBSCl, imidazole, DMAP, DMF, rt, 98%; k) (*S*,*S*)-**L4** (0.05 mol %), AcOH (20 mol %), H_2_O, THF, 46%, er > 99:1; l) (i) TBSCl, imidazole, DMAP, DMF, rt, 98%; (ii) TBAF, THF, rt, 49%; (iii) TEMPO, PhI(OAc)_2_, CH_2_Cl_2_, rt; (iv) Ph_3_P=C(Me)COOEt, CH_2_Cl_2_, reflux, 79% (2 steps); m) H_2_ (1 atm), Pd(OH)_2_, rt, 83%; n) (i) DIBAL-H, CH_2_Cl_2_, 0 °C; (ii) TEMPO, PhI(OAc)_2_, CH_2_Cl_2_, rt; (iii) Ph_3_P=C(Me)COOEt, CH_2_Cl_2_, rt, 63% (3 steps).

The influence of the hydroxylation pattern at the lower side chain on the biological activity of C8-desmethylmycolactones has been thoroughly investigated by the Blanchard group. For those studies, they devised flexible strategies towards the lower mycolactone A/B and C side chains (cf. Scheme 23 and Scheme 25), which enabled the synthesis of several analogs differing in the number and configuration of the hydroxy-substituted carbons [92,242]. As illustrated by the general reaction scheme in Scheme 41A, all syntheses proceeded via the respective ethyl (*E*)-2-methyloct-2-enoate, which was transformed into the corresponding dienyl stannane in analogy to the syntheses shown in Scheme 22 and Scheme 25. CuTC-mediated Stille-type coupling then furnished the full length C1’–C16’ fragments. Different strategies were pursued to provide the hydroxylated ethyl (*E*)-2-methyloct-2-enoates (Scheme 41B–F). C12’,C13’,C15’-trihydroxylated variants with a *syn*,*syn*- or a *syn*,*anti*-configuration at the triol motif were prepared via the same dihydroxylation/partial defunctionalization approach as in Blanchard’s synthesis of the mycolactone A/B lower side chain (exemplified in Scheme 41B). By using different combinations of AD-mix α or AD-mix β in the first and the second dihydroxylation step, respectively, all four possible isomers of **245** with *syn*,*syn* or *syn*,*anti* stereochemistry were prepared. The corresponding *anti*,*anti*- or *anti*,*syn*-C12’,C13’,C15’-stereocluster was prepared from known aldehydes (*R*)- or (*S*)*-***17** by Ando–HWE reaction, furnishing, e.g., α,β-unsaturated ester **246** with a *Z-*configuration (Scheme 41C). The latter was dihydroxylated with AD-mix α, subsequent protection and two-carbon elongation by Wittig chemistry then furnished **248** with an *anti*,*syn*-arrangement of the three hydroxy groups. Furthermore, both enantiomers of the *syn*-diastereomer of the C15’-dehydroxy mycolactone A/B polyenoate chain were prepared (Scheme 41D). Starting from (*E*)-hex-2-enal (**249**) ethyl (2*E*,4*E*)-2-methylocta-2,4-dienoate was prepared by Wittig olefination and the γ,δ-double bond was selectively dihydroxylated, either with AD-mix α or AD-mix β, to obtain **250** or its enantiomer.

TBS protection then furnished *syn*-C12’,C13’-dihydroxylated intermediate **251**, while palladium-mediated allylic reduction of the corresponding carbonate and subsequent TBS protection gave the C13’-monohydroxylated analog **252** (only the (*S*)*-*enantiomer was prepared in this case). The (*R*)-C12’-monohydroxylated derivative was prepared from racemic 1-hexene oxide (**253**) by selective hydrolysis of the (*S*)-enantiomer using Jacobsen’s catalytic kinetic resolution protocol for terminal epoxides (Scheme 41E) [252]. TBS protection of both ensuing hydroxy groups followed by selective cleavage of the primary TBS ether gave the free primary alcohol, which was oxidized to the aldehyde stage and converted into key intermediate **255** by Wittig olefination. Finally, the (*S*)-C15’-hydroxylated derivative **258** was again prepared from aldehyde (*S*)*-***17** by Wittig two-carbon elongation using the Kishi procedure [43] and subsequent hydrogenolytic reduction of the α,β-unsaturated ester intermediate followed by another Wittig elongation cycle (Scheme 41F).

**Scheme 42:**
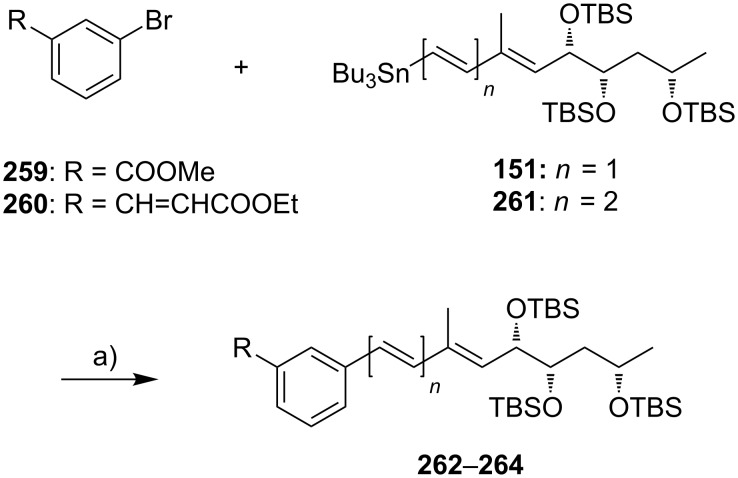
Blanchard’s synthesis of aromatic mycolactone polyenoate side chain analogs. Reagents and conditions: a) Pd(PPh_3_)_4_ (4 mol %), toluene, 110 °C, 86% (**262**: *n* = 1, R = –COOMe) or 92% (**263**: *n* = 1, R = –CH=CHCOOEt), or 81% (**264**: *n* = 2, R = –COOMe).

The Blanchard group has also reported the synthesis of mycolactone analogs with partially rigidized lower side chains, by incorporating phenyl moieties at different positions in the polyenoate chain (**262**–**264**). To this end, dienyl or trienyl stannanes **151** or **261**, possessing the hydroxylation pattern of natural mycolactone A/B, were coupled to *meta*-brominated benzoic acid (**259**) or cinnamic acid esters (**260**) under classical, palladium-based Stille conditions (Scheme 42). Yields were typically high and the ensuing esters were cleaved under the usual conditions.

#### IV.3. Fluorescent and biotinylated mycolactone analogs

**Scheme 43:**
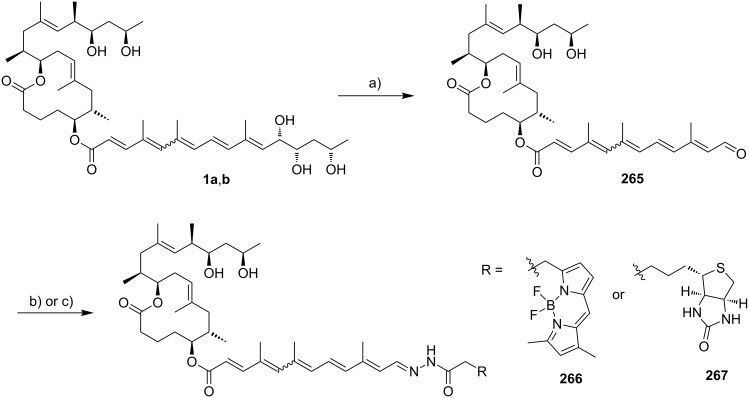
Small’s partial synthesis of a BODIPY-labeled mycolactone derivative and Demangel’s partial synthesis of a biotinylated mycolactone analog. Reagents and conditions: a) Small: HCl (0.1 M), HIO_4_ (0.01 M), EtOH, 37 °C; Demangel: NaIO_4_ (0.01 M), THF/H_2_O 1:1, rt; b) BODIPY hydrazide, CHCl_3_/MeOH 2:1, rt, then HPLC purification; c) biotin hydrazide, CHCl_3_/MeOH 2:1, DMSO, rt, HPLC purification.

Mycolactone analogs featuring a fluorescent BODIPY or a biotin label at the lower side chain have been prepared through semisynthesis by Small [91] and Demangel [93], respectively. Starting from natural mycolactone A/B (**1a**,**b**), both groups exploited a periodate-mediated cleavage of the 1,2-diol moiety in the polyunsaturated side chain to afford the extensively conjugated aldehyde **265** (Scheme 43). Condensation with BODIPY or biotin hydrazide furnished the labeled mycolactone analogs **266** and **267** (no yield given) that were purified by RP-HPLC.

**Scheme 44:**
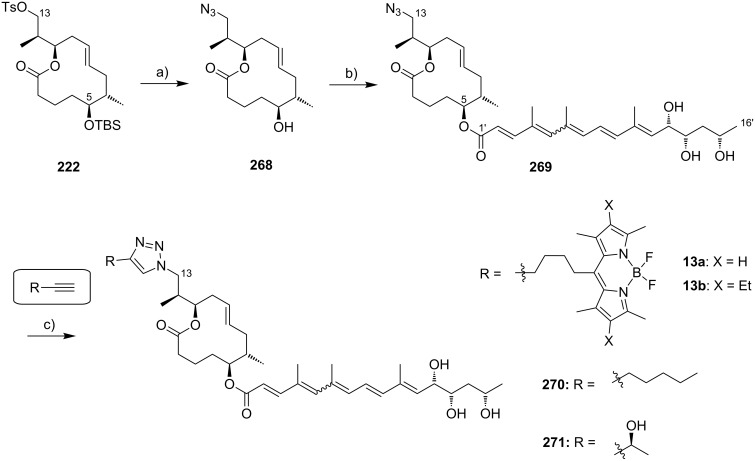
Blanchard’s synthesis of the BODIPY-labeled 8-desmethylmycolactones. Reagents and conditions: a) (i) NaN_3_, DMF, 75 °C, 79%; (ii) TBAF, THF, 74%; b) (i) **122a**,**b**, 2,4,6-trichlorobenzoyl chloride, DIPEA, DMAP, benzene, 85%; (ii) TBAF, THF, 93%; c) alkyne, Cu(OAc)_2_∙H_2_O, sodium ascorbate, *t*-BuOH/H_2_O 5:3, 60 °C (µw), 50% (**270**), 22% (**271**), 35% (**11a**) or 35% (**11b**), *E*-Δ^4’,5’^/*Z*-Δ^4’,5^ ca. 1:1.

C8-desmethylmycolactone analogs tagged with two different BODIPY fluorophores replacing part of the C12–C20 core extension have been disclosed by the Blanchard group (Scheme 44) [92,242]. In order to introduce the fluorescent dye, the tosylate **205** was converted into an azide as a handle for the introduction of various residues by copper-catalyzed Huisgen–Meldal–Sharpless azide–alkyne cycloaddition [253–255]. Subsequently, the lower side chain was introduced by Yamaguchi esterification and deprotected with TBAF, yielding triol **269**. Functionalized BODIPY dyes bearing terminal alkyne groups were prepared according to literature procedures [256] and were clicked on the azide-functionalized mycolactone core equipped with the fully deprotected lower side chain (**269**). This strategy furnished the green and red-fluorescent derivatives **13a** and **13b**, respectively. Non-fluorescent triazoles such as **270** and **271** were also prepared for SAR studies.

**Scheme 45:**
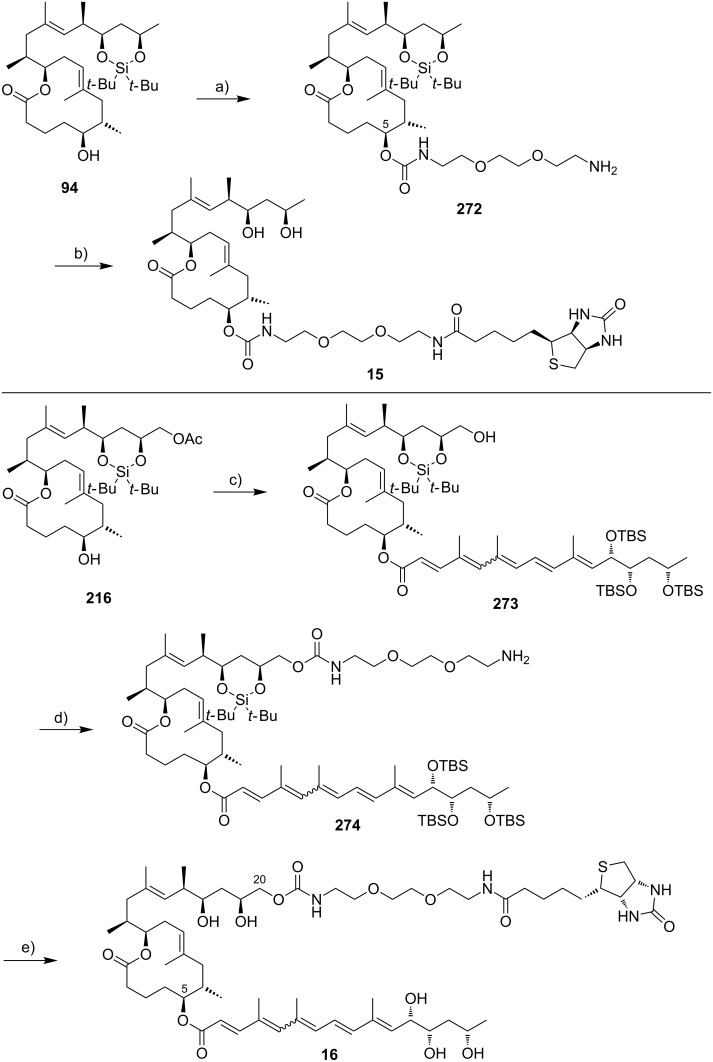
Altmann’s synthesis of biotinylated mycolactones. Reagents and conditions: a) (i) CDI, THF, rt, 2 d, then H_2_O, rt, 45 min, then 1,2-bis(2-aminoethoxy)ethane, rt, 1 d, 81%; b) (i) HF·pyridine, THF/pyridine 4:1, rt, 2 h, 96%; (ii) (+)-biotin, PyBOP, DIPEA, DMF, rt, 30 min, 54%; c) (i) **122a,b**, 2,4,6-trichlorobenzoyl chloride, DIPEA, DMAP, rt, 16 h, 88%; *E*-Δ^4’,5’^/*Z*-Δ^4’,5^’ 4.3:1; (ii) K_2_CO_3_, MeOH, rt, 3 h, 90%; *E*-Δ^4’,5’^/*Z*-Δ^4’,5’^ 4:1; d) (i) CDI, THF, rt, 5 h, then H_2_O, rt, 30 min, then 1,2-bis(2-aminoethoxy)ethane, rt, 90 min, 87%, *E*-Δ^4’,5’^/*Z*-Δ^4’,5’^ 3:1; e) (i) TBAF, THF, rt, 4 h, then NH_4_F, rt, 17 h, quant., *E*-Δ^4’,5^’/*Z*-Δ^4’,5’^ 3:1; (ii) (+)-biotin, DIPEA, PyBOP, DMF, rt, 30 min, 63% *E*-Δ^4’,5^*^’^*/*Z*-Δ^4’,5’^/other isomers 63:29:8.

Very recently, our own group reported two biotinylated mycolactone-derived probes, which were used to gain insight into mycolactones' molecular mechanism of action within the mTor pathway (cf. Figure 6) [111]. Probe **15** possessing a biotin-substituted triethylene glycol-derived linker as a replacement of the lower side chain was prepared starting from secondary alcohol **94**, which was reacted with CDI to give the respective imidazolyl carbamate (Scheme 45).

After quenching unreacted CDI with water, the addition of a large excess of 1,2-bis(2-aminoethoxy)ethane gave carbamate **272**. Cleavage of the cyclic silyl ether with pyridine-buffered HF∙pyridine followed by PyBOP-promoted acylation of the terminal amino group with biotin finally yielded **15**. Probe **16**, which has the biotin linked to C20 of the core extension via the same linker was prepared from secondary alcohol **216** bearing an acetoxy group at C20. Yamaguchi esterification of the C5-hydroxy group with the TBS-protected mycolactone A/B pentaenoate side chain acid and subsequent saponification of the C20-acetoxy group led to primary alcohol **273**. The linker was again introduced via the formation of an imidazolyl carbamate, which was reacted with 1,2-bis(2-aminoethoxy)ethane to give carbamate **274**. Global silyl ether deprotection was achieved by one-pot sequential treatment with TBAF and ammonium fluoride and the ensuing pentol was acylated with biotin at the terminal amino function of the linker using PyBOP as the coupling agent.

### V. Structure–activity relationship (SAR) studies

Although there have been numerous reports on the biological activity of mycolactones, systematic structure–activity relationship (SAR) studies are sparse. The only systematic assessment of analogs with a natural core was conducted by the groups of Altmann and Pluschke [90], while the Blanchard and the Demangel groups have investigated a diverse set of C8-desmethylmycolactones for cytopathogenic activity [92], (N)-WASP inhibition [242] and anti-inflammatory properties [257]. The mutual comparability of these studies is limited, however, since different cell lines (e.g., murine L929 fibroblasts, Jurkat T cells or human cancer cell lines) and readouts (e.g., cell rounding, cytokine production or flow cytometric parameters) were used. In some cases, no complete description of the experimental details is provided, thereby further complicating quantitative comparisons between the studies. Moreover, many studies rely on the determination of activity at a single concentration or give activity thresholds instead of providing IC_50_ or LC_50_ data. Due to the delayed kinetics of mycolactone action (see chapter I), the time point of data collection is of major importance and not consistent between studies. Most studies employed purified natural mycolactones as the standard and the results should thus be treated with care due to potential variations in the degree of purity of the material used. However, results obtained with synthetic mycolactones may also be biased, since mycolactones tend to be very sensitive to light exposure and might even decompose partially when stored at −20 °C for extended time periods [251,258]. Consequently, caution needs to be exercised when comparing results from different reports has to be handled with care.

#### V.1. Natural mycolactones

The cytopathogenic effect (CPE) of mycolactones was first described by Krieg and colleagues who fractionated cell cultures and tested the individual fractions on L929 mouse fibroblasts [29]. The first quantification of the CPE of natural mycolactone A/B (**1a**,**b**) was provided by Small and co-workers in their seminal work from 1999 [32]. According to their data, mycolactone A/B caused cell rounding in murine L929 fibroblasts after 24 h at concentrations as low as 25 pg/mL (34 pM); in addition, detachment of cells from the culture plate accompanied by a growth arrest was observed after 48 h [32]. Cell death via apoptosis was only observed at concentrations of 4 nM or above after 72 h (L929 and J774 cells) [82] and no pronounced effect of inhibitors like genistein (TK inhibitor [259]), PD150606 (calpain inhibitor [260]), mastoparan or and suramin T (G-protein inhibitors [261–262]) or wortmannin (PI3K inhibitor [263]) on cytopathogenicity could be detected [91]. Interestingly, concomitant treatment of cells with a caspase inhibitor prevented apoptosis, but not the cytopathogenic phenotype, indicating that apoptosis might be a secondary effect [82]. In a more recent detailed analysis of the biological activity of synthetic mycolactone A/B on L929 fibroblasts, Pluschke and co-workers observed a similar time-dependent phenotype as had been reported in this earlier work. Based on DNA staining with 4’,6’,-diamidino-2-phenylindole (DAPI) [264] and by using a fluorescent derivative of the selective F-actin-binding peptide phalloidin [265] they could also show that the morphological changes were accompanied by DNA fragmentation and depolymerization of the actin cytoskeleton [90,178]. Mycolactone concentrations ≥10 nM were found to be cytotoxic after 48 h and 72 h, respectively, while no effect was observed at 5 nM, independent of the duration of treatment. After exposure to mycolactone concentrations of 20 nM for 48 h, more than 90% of the cells displayed either apoptotic (A^+^/PI^−^) or necrotic (A^+^/PI^+^) properties as determined via flow cytometry after annexin-V-FITC (A) [266] and propidium iodide (PI) staining (see also Table 2) [267]. Furthermore, cellular metabolic activity was strongly inhibited by mycolactone A/B (IC_50_ = 5 nM) as determined by AlamarBlue^®^ (resazurin) staining/flow cytometry and a complete shutdown of proliferation was observed in L929 cells at concentrations of 81 nM [90]. Using a panel of 39 human tumor cell lines, the Kishi group found selective cytotoxicity of mycolactone A/B (LC_50_ = 89 nM) against human LOX-IMVI melanoma cells, while no other cell line was significantly affected below 10 µM (no experimental details were provided) [268]. The groups of Leadlay and Demangel further demonstrated that mycolactone A/B suppresses cytokine production in Jurkat T cells [63,93] and several other immune cell lines [257], with IC_50_ values in the low nanomolar range. In contrast, no antimicrobial activity of mycolactone A/B against *Streptococcus pneumoniae* (Gram positive), *Escherichia coli* (Gram negative), *Saccharomyces cerevisiae*, or *Dictyostelium discoideum* was detected [90].

Already in 2003, Small and co-workers recognized the importance of the hydroxy groups at the lower side chain of mycolactone A/B for activity and they concluded that the cytopathogenicity of mycolactones declines with decreasing polarity [47]. In the same study it was shown that mycolactone C (**2**), which was later shown to lack the C12’-hydroxy group [53], caused the typical cytopathogenic mycolactone A/B phenotype in murine L929 fibroblasts, albeit at much higher concentrations (8 × 10^5^-fold) [47]. Of note, this conclusion is based on a CPA of 0.01 ng/mL (0.014 nM) of mycolactone A/B. In subsequent studies from the same group the CPA of mycolactone A/B was reported as 1 ng/mL. Contrary to these earlier findings, Pluschke and co-workers showed by flow cytometry that synthetic mycolactone C (LC_50_ = 186 nM, IC_50_ = 122 nM, see Table 2) was only 16 times less cytopathogenic than synthetic mycolactone A/B [90], while Leadlay and co-workers found a significantly decreased suppression of phorbol 12-myristate-13-acetate (PMA)/ionomycin (IO)-stimulated IL-2 production in Jurkat T cells compared with mycolactone A/B [63]. Similarly to mycolactone C (**2**), natural mycolactone E (**6**) caused an identical cytopathogenic phenotype as mycolactone A/B at approximately 100-fold higher concentrations, when tested in the same L929 cell assay system (135 nM vs. 1.4 nM) [50]. Moreover, **6** showed a stronger suppression of PMA/IO-stimulated IL-2 production (EC_50_ ca. 130–270 nM) than mycolactone C (**2**), F (**8**), and G (**10**) [63]. For synthetic mycolactone E (**6**), a GI_50_ of approximately 15 nM was reported by Kishi and co-workers on L929 fibroblasts, but no details on the assay conditions were provided in the corresponding publication [58]. Under the same (unspecified) conditions, the synthetic minor C13’-oxo metabolite of mycolactone E (**7**) was shown to be equipotent (GI_50_ = 15 nM) with the parent compound [58]. Natural mycolactone F (**8**) also caused the typical cytopathogenic phenotype in L929 cells at 14 nM [55]; LC_50_ and IC_50_ values of 29 nM and 9 nM, respectively, have been reported for the synthetic compound (see Table 2) [90]. This potent activity is remarkable, considering that mycolactone F (**8**) has a shortened tetraenoate side chain with an inverted stereochemistry at the C11’–C13’ diol moiety. Similar to mycolactone A/B [35], natural mycolactone F (**8**), was found to cause necrosis in L929 fibroblasts at 20 µM after a 4 h treatment, while substantial apoptosis was detected after 24 h at 100-fold lower concentrations [55]. The suppression of stimulated IL-2 production caused by mycolactone F was slightly lower than for mycolactone E (**7**) [63]. Synthetic mycolactone *dia*-F (**9**) has been reported to possess a similar biological profile as mycolactone F (**8**), albeit with 1000-fold reduced potency; details on the effects of mycolactone *dia*-F (**9**) remain to be published [61]. Of note, neither mycolactone A/B nor mycolactones C, E, F or G caused detectable apoptosis in Jurkat T cells at 1.4 µM after 24 h [63]. Within this set of compounds, mycolactone G (**10**) was the weakest suppressor of IL-2 production with an EC_50_ above 700 nM [63]. Currently, no data on the biological activity of mycolactones D (**3**), S1 (**4**) and S2 (**5**) are available.

#### V.2. Synthetic and semisynthetic mycolactones with an unmodified core

**Figure 9:**
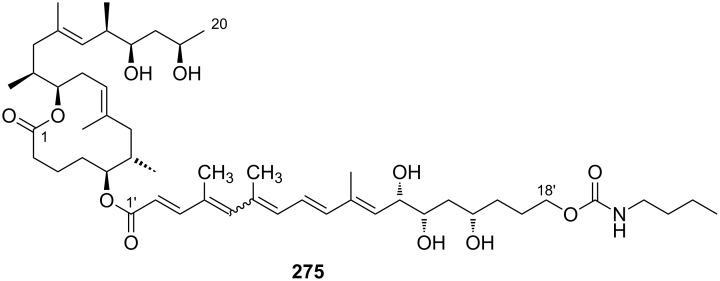
Kishi’s elongated *n*-butyl carbamoyl mycolactone A/B analog.

Early SAR data on chemically modified natural mycolactone A/B (**1a**,**b**) were reported by the Small group, including peracetylated and fully saturated analogs (obtained by exhaustive hydrogenation of double bonds). These modifications caused complete ablation of cytopathogenic activity [32]. In a later study, Small and Snyder, hypothesizing that the trihydroxy motif was simply an “inactive hydrophilic portion of the toxin”, reported the oxidative cleavage of the C12’,C13’ diol motif and used the resulting aldehyde to introduce a fluorescent BODIPY dye by means of hydrazone formation (vide supra). Interestingly, both the aldehyde **265** and the fluorescent derivative **266** (Scheme 43) maintained substantial cytopathogenic activity, which was only reduced by a factor 6–10 compared to the natural product [91]. The extended core lactone, obtained by base hydrolysis of natural mycolactone A/B, also induced a nearly identical cytopathogenic phenotype, but only at 10^6^-fold higher concentrations [47] (again, this conlusion was based on CPA for mycolactone A/B of 0.01 ng/mL). A C12’-biotinylated derivative from Demangel and co-workers, which was also obtained from aldehyde **265**, displayed only slightly decreased cytotoxicity on HeLa cells and suppressed induced IL-2 production in Jurkart T cells with comparable potency as natural mycolactone A/B [93]. In 2011, Kishi reported an isolated example of a synthetic mycolactone derivative with an elongated lower side chain (C1’–C18’) bearing a terminal *n*-butyl carbamoyl group (**275**, Figure 9) [268].

The compound was cytotoxic at 30 nM against L929 mouse fibroblasts, corresponding to a three-fold decrease relative to mycolactone A/B. This indicates that an extension of the lower side chain is well tolerated. Unfortunately, no details on assay conditions were provided in Kishi's paper.

The Kishi group has also reported that photo-mycolactones possess significantly reduced toxicity, but details were only reported for photo-mycolactone A1 (**276**); the latter was tested against five human and murine cell lines (Table 1). Due to the 100–1000-fold drop in activity, compared to mycolactone A/B, the detoxification of mycolactones by light was suggested, and the idea of stabilizing mycolactones by partial saturation of the conjugated double bond system evolved. Two such compounds, α-**277** and β-**277**, which differ from mycolactone A/B by the saturation of the C6’–C7’ double bond were reported by Kishi and co-workers in a very recent paper [251]. Indeed, both epimers exhibit significantly increased stability against light, heat, acid, and base, while preserving some cytotoxicity. The antiproliferative activity of these compounds was assessed against three human cancer lines and L929 mouse fibroblasts (Table 1).

**Table 1:** Antiproliferative activities of photo-mycolactone A1 and C6’–C7’ dihydromycolactones (IC_50_ or GI_50_ values [nM]).

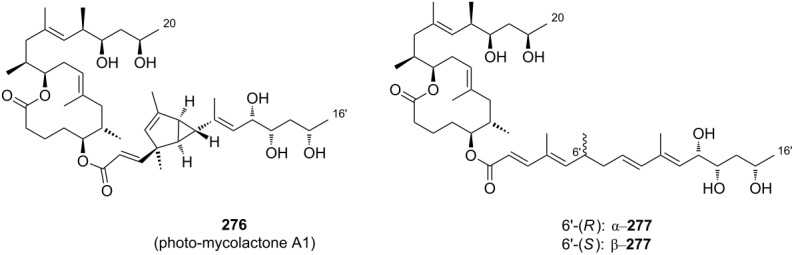						

Mycolactone	L929	HEK-293	LOX-IMVI^a^	A-549	SK-MEL-5^b^	SK-MEL-28^b^

**1a/b**	13^a,b^	3.2^a^/3.3^b^	6.9	0.77^a^/4.7^b^	12	4.5
**276**	2020^b^	2510^b^	–	3820^b^	3600	470
α-**277**	63^a^	83^a^	129	400^a^	–	–
β-**277**	53^a^	3.0^a^	29	77^a^	–	–-

^a^Cytotoxicity (IC_50_) according to [251]. ^b^Growth inhibition (GI_50_) according to [244].

Most notably, the β-epimer exhibited almost the same activity against human embryonic kidney (HEK) 293 cells as mycolactone A/B, but was significantly less potent against the other three cell lines. Generally, the cytotoxicity of the α-epimer was approximately 3–30-fold decreased compared to the β-epimer, with the exception of the mouse L929 fibroblasts cell line, where both epimers were almost equipotent.

In two studies on synthetic mycolactone analogs, Altmann, Pluschke, and co-workers reported on the effects of modifications at the lower side chain and the core extension while leaving the core structure unchanged (Table 2) [90,178]. Biological activity on L929 fibroblasts was evaluated by flow cytometry (A/PI and AlamarBlue^®^ staining) and fluorescence microscopy (DAPI and phalloidin staining as described above). For all compounds, except the C5–O sorbate ester **278**, concentrations required to induce cytotoxicity, reduction of metabolic activity, rearrangements in the actin cytoskeleton and the nuclear morphology were in the same range. Generally, a significant reduction in biological activity was observed if the lower side chain was truncated. For example, both, the C5–O acetyl-capped mycolactone core **279** and analog **280**, which incorporates a C1’–C16’ pentaenoate side chain lacking all three hydroxy groups showed little effects up to concentrations in the low micromolar range. Interestingly, analog **278** was only moderately cytotoxic (LC_50_ = 3426 nM), while being a potent inhibitor of metabolism (IC_50_ = 171 nM). The antiproliferative activity of **278** was significantly lower than for mycolactone A/B, but higher than for **279**, which did not show any measurable antiproliferative activity up to the highest concentration tested (5 μM).

**Table 2:** Biological activities of mycolactones A/B, C, F, and of mycolactone analogs.

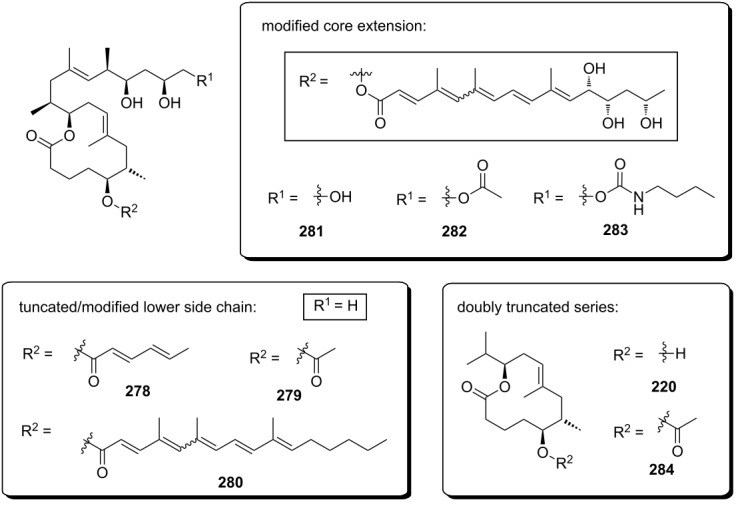			

Mycolactone	LC_50_ [nM]^a^	IC_50_ [nM]^b^	LC_50_/IC_50_

**1a/b**	12	5	2.4
**2**	186	122	1.5
**8**	29	9	3.2
**278**	3426	171	20
**279**	>>5000	>>5000	n.a.
**280**	4550	1439	3.2
**281**	15	5	3.0
**282**	45	20	2.3
**283**	50	16	3.1
**220**	inactive	n.d.	n.a.
**284**	inactive	n.d.	n.a.

^a^Cytotoxicity (LC_50_) determined after 48 h by flow cytometry employing annexin-V-FITC (A) and propidium iodide (PI) staining. ^b^Reduction of metabolic activity (IC_50_) analyzed by AlamarBlue^®^ staining. All experiments were carried out with L929 mouse fibroblasts.

Derivatives modified at the C20 position of the core extension were generally equipotent (**281**) or only slightly less active (**282**, **283**) than mycolactone A/B (**1a**,**b**). Since even an *n*-butyl carbamoyl substituent at C20 atom (**283**) was well tolerated, it can be assumed that this position is well suited for the introduction of tags enabling the deconvolution of mycolactones’ cellular fate and its targets. Simultaneous truncation of the core extension and the lower side chain was deleterious to activity; thus, both **220** (see also Scheme 35) and **284** were devoid of measurable cytopathogenic or apoptosis-inducing effects [178].

#### V.3. Synthetic mycolactones with a C8-desmethylmycolactone core

Extensive work on the SAR of C8-desmethylmycolactones was performed in a joint effort by the groups of Blanchard and Demangel. In an initial study, the Blanchard group prepared a series of seven C8-desmethylmycolactone derivatives for studying the effects of different substitution patterns at the C12’,C13’,C15’-stereocluster, as well as the removal of the C14–C20 part of the core extension and/or the lower side chain [92]. Cytopathogenicity was analyzed at 10 µM and 50 µM and the minimum concentration required to induce 90% cell rounding was determined for natural mycolactone A/B (**1a**,**b**, 40 nM) and the synthetic C8-desmethyl analog **285** (5000 nM). Although the computationally predicted 3D conformations of the C8-desmethyl and the unmodified mycolactone core were virtually identical [182], a 125-fold drop in cytopathogenic activity was observed when removing the C8-methyl group. Therefore, the comparison of activities between mycolactones possessing an unmodified and a C8-desmethyl core, respectively, is hardly conclusive and it cannot be ruled out that C8-desmethyl analogs engage a different set of targets in vivo. Consequently, those SAR are treated separately in this review.

For Blanchard’s first set of C8-desmethylmycolactones, significant changes in cytopathogenicity were observed when the C12’,C13’,C15’-stereocluster was modified (Table 3) [92].

**Table 3:** Cytopathogenic activities of C8-desmethylmycolactone analogs.

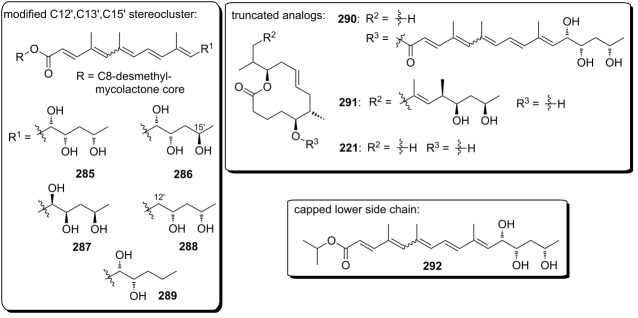		

Mycolactone	% Cell rounding at 10 µM^a^	% Cell rounding at 50 µM^a^

**1a/b**^b^	100	100
**285**^c^	100	100
**286**	100	100
**287**	10	100
**288**	49	100
**289**^d^	40	n.d.
**290**	53	100
**291**	27	100
**221**	10	20
**292**	5	15
**13a**^e^	90	n.d.

^a^Cytopathogenicity determined after 48 h as the number of rounded cells compared to the total number of cells. ^b^Minimum concentration required for 90% cell rounding after 24 h: 40 nM. ^c^ Minimum concentration required for 90% cell rounding: 5 µM. ^d^Data from [242] and [182]. ^e^No cell rounding was detectable at 0.5 µM, the concentration at which cellular uptake was assessed.

Inverting the stereochemistry at the C15’-hydroxy group (**286**) maintained full cytopathogenicity (100%) at both tested concentrations, while inversion of the configuration of the entire C12’,C13’,C15’-stereocluster (**287**) decreased cytopathogenic activity to 10% at 10 µM; at 50 µM full cytopathogenic activity was retained. Removal of the C12’-hydroxy group (**288**) also decreased cytopathogenicity, albeit to a lower extent (49% at 10 µM). Interestingly, the removal of the C15’-hydroxy group (**289**) had a higher impact on the cytopathogenic activity, which was reduced to 40% at 10 µM [182,242]. The truncation of the core extension (**290**) had a similar effect as the removal of the C12’-hydroxy group, leading to 53% cytopathogenicity at 10 µM, while maintaining full cytopathogenic activity at 50 µM. A slightly more pronounced drop in cytopathogenic activity was seen if the lower side chain was removed, while keeping the core extension (**291**, 27% at 10 µM and 100% at 50 µM). Removing both the core extension and the polyenoate side chain (**221**, see also Scheme 35) was detrimental to activity. Similarly, the isopropyl ester of the lower side chain acid **292** was virtually inactive. Interestingly, the click chemistry-derived fluorescent analog **13a** (see Scheme 44 and Figure 5) had a cytopathogenic activity of 90% at 10 µM, thus maintaining most of the cytopathogenicity of the parent compound **285**.

In a subsequent study, the Blanchard and Demangel groups investigated the binding of a series of 27 C8-desmethylmycolactone analogs to (N)-WASP [242]. Due to the amount of work presented in [242], not every single analog will be discussed here. Based on the experiments described in this paper, natural mycolactone A/B (**1a**,**b**) binds to N-WASP with an approximate *K*_d_ value of 170 nM, as estimated indirectly by measuring the dependence of the increase in the maximal rate of actin assembly on mycolactone concentration. The binding affinity (*K*_d_) of natural mycolactone A/B to the CR1 domain of WASP and the CR7 domain of N-WASP was reported to be 20 nM and 66 nM, respectively [93,182]. Binding of C8-desmethylmycolactone analogs was assessed by displacement of the C12’-biotinylated mycolactone A/B derivative **267** (see Scheme 43) from immobilized isolated (N)-WASP mycolactone binding domains (MBDs), as they had been defined previously [93]. Data are reported in [93] only for binding to the WASP-MBD, but comparable results were also obtained with the corresponding N-WASP domain (that is not shown in the paper). The IC_50_ value of mycolactone A/B in this displacement assay was 32 µM (Table 4) [242]. Compared to the 125-fold reduced cytopathogenicity of C8-desmethylmycolactone analog **285** [92], only a three-fold reduction in affinity was observed for the WASP-MBD (IC_50_ = 98 µM). Similarly, C8-desmethylmycolactone derivatives **286**, **287**, and **289**, with modifications in the C12’,C13’,C15’ stereocluster (for structures cf. Table 3) had IC_50_ values in the range between 30 µM and 70 µM. Thus, the influence of the stereochemistry and substitution pattern at the C12’, C13’, and C15’-positions on WASP affinity seems to be less pronounced than on cytopathogenicity. Similar observations were made with compounds from the series devoid of the larger part of the core extension. Interestingly, derivative **290**, which lacks the C14–C20 segment of the core extension, showed an IC_50_ of 22 µM and, thus, was more potent than mycolactone A/B. This observation is in conflict with the original cytopathogenicity data that have been reported for this compound [92], which showed a profound drop in activity upon removal of the core extension. Both, the truncated and the extended C8-desmethylmycolactone core (**291** and **221**, respectively) with a free C5-hydroxy group showed no displacement of the reporter under the conditions tested (IC_50_ > 1000 µM). In contrast, full cytopathogenic activity was observed for **291** at 50 µM (cf. Table 3) [92]. Among all analogs with a truncated core extension that have been investigated so far, the most potent representative was found to be **295**, which features an inverted configuration at the C13’ atom and which showed an IC_50_ of 10 µM in the displacement assay. The affinity of compound **293** (IC_50_ = 34 µM), which comprises the all-epi mycolactone A/B side chain differed only slightly from the natural stereoisomer **290** (IC_50_ = 22 µM) and a similar affinity was observed for the C15’-epimer **294** (IC_50_ = 44 µM). While the removal of the C15’-hydroxy group in analog **296**, with the natural stereochemistry for the two other hydroxy-bearing stereocenters remaining unchanged, was detrimental to binding (IC_50_ = 250 µM), the inversion of both the C12’ and the C13’-stereocenters (**297**, IC_50_ = 27 µM) completely rescued affinity.

**Table 4:** Biological activities of C8-desmethylmycolactone analogs on WASP.

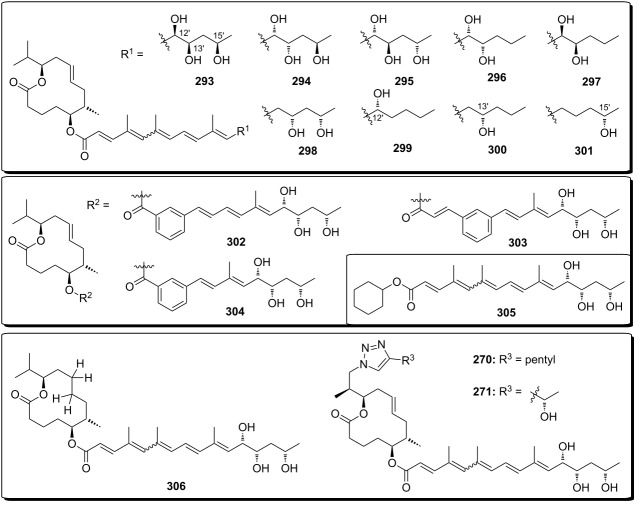			

Mycolactone	IC_50_ [µM]^a^	Mycolactone	IC_50_ [µM]^a^

**1 a/b**	32	**297**	27
**285**	98	**298**	28
**286**	41	**299**	350
**287**	33	**300**	23
**289**	70	**301**	75
**290**	22	**302**	60
**291**	>1000	**303**	35
**221**	>1000	**304**	70
**293**	34	**305**	135
**294**	44	**306**	65
**295**	10	**270**	35
**296**	250	**271**	170

^a^IC_50_ values were determined as the capacity to displace biotinylated mycolactone **267** (1 µM) from immobilized GST-fused WASP mycolactone binding domains (amino acids 200–313). Testing compounds were used in concentrations between 0 µM and 10 µM (intervals were not defined).

In contrast, analog **298**, possessing a C13’,C15’-dihydroxy substitution pattern with the natural configuration was highly active (IC_50_ = 28 µM) in spite of the missing C12’-hydroxy group. Intriguingly, compound **300** which bears a single hydroxy group at the C13’ position (IC_50_ = 23 µM) was even more potent than natural mycolactone A/B. The removal of both, the C13’ and the C15’-hydroxy groups (**299**), led to a strong erosion of affinity (IC_50_ = 350 µM), while removal of the C12’ and C13’ hydroxy groups in derivative **301** had a much lower impact (IC_50_ = 75 µM). With IC_50_ values between 35 µM and 70 µM, derivatives **302–304**, which incorporate a *meta*-substituted phenyl ring as a rigid diene bioisostere retained similar activity as natural mycolactone A/B. Quite remarkably, the cyclohexyl ester of the mycolactone A/B lower side chain (**305**) preserved significant activity. With an IC_50_ of 135 µM, an approximately four-fold reduction in affinity was observed compared to the parent compound **1a**,**b**. This observation stands in sharp contrast to the almost complete loss in cytopathogenic activity of the corresponding isopropyl ester **292** (cf. Table 3). Saturation of the C8–C9 double bond in the truncated 8-desmethylmycolactone core was well tolerated and the activity of compound **306** (IC_50_ = 65 µM) was only three-fold lower than for its C8–C9 unsaturated counterpart **290**. The effect of a 4-substituted 1,2,3-triazole moiety attached to the C13 position was strongly dependent on the nature of the substituent. While an unsubstituted pentyl chain was well tolerated (**270**, for structures see also Scheme 44, IC_50_ = 35 µM), an (*S*)-2-hydroxyethyl substituent caused a substantial drop in activity (**271**, see also Scheme 44, IC_50_ = 170 µM). Compound **290**, the closest structural analog of the natural toxin from the series lacking the C14–C20 part of the core extension, was analyzed for its capability to disrupt the (N)-WASP-VCA interaction. It was shown that **290** displaces the VCA domain from an immobilized GST-fused version of the WASP mycolactone binding domain in a dose dependent manner. IC_50_ values for both, mycolactone A/B and **290** were in the low micromolar range as determined by electrophoresis of the pulled down products. Similar observations were made for N-WASP (no data provided in [242]). The adhesion capacity of HeLa cells was also reduced by **290** albeit at much higher concentrations as with the natural toxin (16 µM vs 26 nM). Of note, the unsubstituted extended mycolactone core **291**, which is devoid of WASP binding affinity, did not alter cell adhesion at the same concentrations (no data shown in [242]).

Collectively, it can be concluded from these SAR studies that neither the core extension nor the C8-methyl group of mycolactone A/B are required for (N)-WASP binding, while the lower side chain is crucial. The impact of the stereochemistry at the C12’, the C13’, and the C15’-positions on (N)-WASP affinity is much less pronounced than would have been expected on the basis of earlier cytopathogenicity studies. In fact, the effects on WASP affinity are sometimes opposite to the changes in cytopathogenicity observed for the same modifications [59,90,92]. Even the removal of one or two hydroxy groups, including the C12’-hydroxy group, which had been found to be of crucial importance in other studies [47,50,63,92], was tolerated in certain cases. Likewise, the inclusion of a *meta*-substituted phenyl ring in the lower side chain and the replacement of the core extension by a 4-alkyl-substituted 1,2,3-triazole is tolerated. Overall, the SAR for WASP binding were relatively flat and significantly more pronounced effects of modifications causing relatively minor changes in WASP binding had been observed in previous studies using cellular readouts [47,63,90,92]. Since cytopathogenic activity and (N)-WASP binding hardly correlate and the concentrations of **290** used in confirmatory cellular experiments were very high, it can be debated to which extent the interaction with (N)-WASP contributes to the cellular mycolactone phenotypes. In this context, it is worth noting that the involvement of mycolactone-promoted WASP activation in the blockage of proinflammatory cytokine production has recently been questioned [89,111].

In their most recent contribution to the SAR of mycolactones, the groups of Blanchard and Demangel have dissected the immunosuppressive and cytotoxic properties of selected representatives from the set of C8-desmethylmycolactone analogs discussed above [257]. Notably, all of the molecules included in this recent study were devoid of the C14–C20 segment of the core extension. As shown in Table 5, immunosuppressive activity of mycolactone analogs was determined as the ability to block PMA/IO-induced IL-2 production in Jurkat T cells (expressed as IC_50_), while cytotoxicity was determined as the capacity to provoke detachment-induced cell death in HeLa cells (in %) after 48 h at a fixed concentration (16 µM). As expected, all variants were less active than natural mycolactone A/B, which killed >80% of the cells at 16 µM and suppressed IL-2 production with an IC_50_ of 40 nM (Table 5). Unsurprisingly, the free extended core structure with a truncated upper side chain (**221**) and the cyclohexyl ester of lower mycolactone A/B side chain acid (**305**) exhibited no IL-2 suppressive activity up to 4 µM, the maximum concentration tested.

**Table 5:** Cytotoxicity and immunosuppressive properties of C8-desmethylmycolactones.

Mycolactone	% Cytotoxicity at 16 µM^a,b^	IC_50_ [µM] (IL-2 production)^b,c^

**1a**,**b**^d^	85	0.040^e^
**285**	80	>4
**290**	50	1.5
**221**	10	>4
**296**	60	2.7
**297**	80	2.8
**298**	40	3.7
**299**	30	>4
**300**	43	>4
**301**	40	>4
**302**	57	3.2
**303**	70	1.7
**304**	65	>4
**305**	45	>4
**306**	80	1.7
**270**	80	1.7

^a^Cell viability in HeLa cells after 48 h at a mycolactone concentration of 16 µM. ^b^All numbers represent approximate values that have been manually extracted from a plot since no numerical data was given. ^c^Suppression of PMA/IO-induced IL-2 production in Jurkat T cells.^d^Natural mycolactone A/B was used in this study. ^e^Data from [87].

The SAR of the remaining compounds cannot be easily rationalized and there was no obvious correlation between IL-2 suppressive activity and WASP affinity. For example, C8-desmethylmycolactone A/B **285**, the structurally closest analog of the natural compound within this set, was inactive at 4 µM. In contrast, its close analog **290**, which lacks the C14–C20 segment of the core extension, was the most potent derivative with an IC_50_ of 1.5 µM. The effects of configurational changes at the C12’, the C13’, and the C15’ stereocenters were rather ambiguous and IC_50_ values (in the narrow range) between 1.5 µM and >4 µM were determined for different trihydroxylated derivatives. All derivatives bearing only a single hydroxy substituent at the lower side chain (**299**–**301**) were inactive at 4 µM, while all dihydroxylated derivatives (**296**–**298**) had IC_50_ values between 2.5 µM and 4 µM. Likewise, derivatives incorporating a phenyl residue as part of the lower side chain were found to display a range of potencies; while compound **303** was among the most immunosuppressive derivatives tested (IC_50_ ≈ 1.7 µM), **302** was only moderately active (IC_50_ ≈ 3.2 µM) and **304** belonged to the group of inactive analogs (IC_50_ > 4 µM). Most notably, derivative **306**, which features a saturated C8-desmethyl C1–C13 mycolactone core and 4-pentyltriazolyl-substituted derivative **270** were among the most potent suppressors of IL-2 production, both with IC_50_’s around 1.7 µM. At the same time, the latter compounds belong to the most cytotoxic analogs within this series (80% reduction in cell viability at 16 µM). In this context, it is worth mentioning that the IL-2 suppressive properties of all compounds investigated are significantly less pronounced than immunosuppressive effects of mycolactone E (**6**) or F (**8**) that have been determined under similar assay conditions by Leadlay and co-workers [63]. The comparability of the cytotoxicity data within the compound set is hampered by the fact that only a single concentration was tested. Despite these constraints, it is interesting to note that only a limited correlation could be observed between the immunosuppressive activity and cytotoxicity. For example, 8-desmethylmycolactone A/B (**285**) was relatively cytotoxic (≈80% reduction in cell viability at 16 µM), while being weakly immunosuppressive (IC_50_ > 4 µM). In contrast, truncated 8-desmethylmycolactone A/B **290** was less cytotoxic (≈50% reduction in cell viability at 16 µM), but a more potent immunosuppressant (IC_50_ = 1.5 µM). Similarly, no clear correlation between structure and activity was found. The only clear trend was that low cytotoxicity (<50% at 16 µM) was associated with the presence of a single hydroxy group at the lower side chain (**299**–**301**); this was also observed for ester **305** or the isolated (partially extended) C1–C13 core (**221**).

Despite the relatively flat SAR, derivative **290** was selected for further investigations, since it provided the best ratio between the suppression of IL-2 production and cytotoxicity. Compound **290** was tested for its capability to suppress stimulated TNFα, IL-2 or INF-γ production in polymorphonuclear neutrophils (PMN), monocyte-derived macrophages (MDM) and in CD4^+^ T cells. Cytotoxicity against MDM cells and primary human dermal fibroblasts (HDF) as well as AT_2_R binding in AT_2_R-transfected HEK cells were also assessed (Table 6). IC_50_ values for **290** were generally in the low micromolar range in all assays, while values in the low nanomolar range were typically observed for mycolactone A/B.

**Table 6:** Diverse biological activities of mycolactone A/B (**1a**,**b**) and **290** (IC_50_ [nM]).

Mycolactone	TNFα^a^	TNFα^b^	Cell viability^c^	IL-2^d^	INF-γ^e^	AT_2_R binding^f^

**1a**,**b**	13	12	18/6	12	7	9200
**290**	2000	3500	9000/12000	3900	1800	16000
IC_50_ ratio	162	285	514/1910	320	268	2

^a^Suppression of PMA-induced TNFα production in PMN cells. ^b^Suppression of LPS-induced TNFα production in MDM cells. ^c^Cell viability assessed by an MTT reduction assay after 72 h of incubation (MDM/HDF cells). ^d^Suppression of PMA/IO-induced IL-2 production in CD4^+^cells. ^e^Suppression of PMA/IO-induced IFN-γ production CD4^+^ cells. ^f^Competitive binding to human AT_2_R against 0.01 nM of the agonist radioligand [^125^I]-CGP42,112A (*K*_d_ = 0.01 nM) in transfected HEK cells.

A notable exception was AT_2_R binding, where mycolactone A/B and **290** showed similar potency (16 µM vs 9 µM, respectively) for displacing the peptidic agonist radioligand [^125^I]-CGP42,112A. Furthermore, while **290** was between 160- and 320-fold less potent than mycolactone A/B (**1a**,**b**) as an inhibitor of cytokine production, a roughly 500- and 2000-fold decreased toxicity against MDM and HDF cells, respectively, was observed, suggesting a slightly increased selectivity window for **290**. However, both mycolactone A/B and **290** were non-toxic to PMN and CD4^+^ cells at immunosuppressive concentrations. In a mouse model for PMA-induced chronic skin inflammation, injection of mycolactone A/B showed a marked reduction in inflammatory response at 0.5 mg/kg, while a less pronounced effect was observed for 5 mg/kg of **290**. In contrast, 5 mg/kg of **290** were similarly effective as 0.5 mg/kg of mycolactone A/B in relieving inflammatory pain. Both compounds had little effect on acute pain in a mouse model relying on formalin injection. A mouse model for rheumatoid arthritis also demonstrated a moderate anti-rheumatic effect of natural mycolactone A/B, while **290** was not tested in this model. The observation that the cytotoxicity of mycolactones can at least be partially dissociated from their immunosuppressive and pain-relieving properties is interesting. However, its significantly reduced potency and the still narrow range between desired and undesired activities in combination with the complexity of **290** will likely prevent its (preclinical) development.

## Conclusion

The complex structure of mycolactones has inspired many chemistry groups to develop elegant approaches towards the conserved extended mycolactone core structure and the variable lower side chain. Until now, all known natural mycolactones except mycolactone D were prepared by means of total synthesis and their structures have been validated. Moreover, the fascinating biology of mycolactones, which possess cytotoxic, immunosuppressive and analgesic properties, has stimulated the synthesis of modified analogs, that have been used to study structure–activity relationships and to decipher the molecular targets of these polyketides. Numerous natural mycolactones and synthetic derivatives have been tested for their biological activities in a variety of assay systems and fluorescent probes have unveiled the cellular localization of mycolactones. It has been shown that the effect of mycolactone exposure varies substantially between different cell lines and is highly dependent on the particular read-out employed. These observations may point to the involvement of several molecular targets in mycolactone bioactivity, in addition, those targets may have different expression levels and/or functions in different cell types. Four of these targets, namely (N)-WASP, the AT_2_R receptor, the Sec61 translocon and the mTOR signaling pathway have been identified to date. However, so far, only the effects on (N)-WASP have been addressed by systematic SAR studies. Generally, the activity of mycolactones is highly sensitive to even minor structural changes at certain hotspots. This is impressively highlighted by the 125-fold drop in cytopathogenic activity upon removal of a single methyl group in the C8 position of the mycolactone core. Likewise, subtle changes in the hydroxylation pattern and the stereochemistry of the C5–O-linked lower side chain can have a major (lowering) impact on biological activity. In contrast to the lower side chain, the core extension seems to be more amenable to biologically tolerated modifications. For example, extensions at the C20 position can be introduced without appreciable effects on cytotoxicity and even the almost complete removal of the core extension is tolerated with regard to AT_2_R receptor binding. The complex structure of mycolactones still leaves room for a plethora of structural modifications that will hopefully allow a further dissociation of the desired anti-inflammatory and pain-relieving properties from the pro-apoptotic effects considered responsible for Buruli ulcer pathology. To achieve this goal, further specific SAR studies on all known (and potential, unknown) molecular mycolactone targets would be highly desirable. The quest for a mycolactone-based therapy, however, is complicated by the enormous complexity even of simplified mycolactone analogs. Furthermore, the metabolically labile ester bond connecting the macrolactone core to the pharmacologically highly relevant lower side chain might hamper systemic application of such compounds and might necessitate bioisosteric replacement. However, even if endeavors towards mycolactone-derived therapeutics remain futile, the detailed knowledge on the molecular mycolactone targets, the underlying pathways and how these are linked to biological effects might stimulate the search for novel, drug-like molecules modulating those networks, thereby fueling the drug pipeline.

## Acknowlegements

The authors are grateful to Lukas Leu, Barbara Stoessel, Jennifer Mueller, Simon Glauser and Adriana Edenharter for proofreading. Moreover, we want to thank Dr. Philipp Gersbach, Dr. Claudio Bomio, Dr. Jun Li, Amina Salihovic and Pascal Bucher for their contributions to the (unpublished) syntheses of mycolactones presented in this review.
